# Ethoscopes: An open platform for high-throughput *ethomics*

**DOI:** 10.1371/journal.pbio.2003026

**Published:** 2017-10-19

**Authors:** Quentin Geissmann, Luis Garcia Rodriguez, Esteban J. Beckwith, Alice S. French, Arian R. Jamasb, Giorgio F. Gilestro

**Affiliations:** 1 Department of Life Sciences, Imperial College London, London, United Kingdom; 2 Polygonal Tree Limited, London, United Kingdom

## Abstract

Here, we present the use of ethoscopes, which are machines for high-throughput analysis of behavior in *Drosophila* and other animals. Ethoscopes provide a software and hardware solution that is reproducible and easily scalable. They perform, in real-time, tracking and profiling of behavior by using a supervised machine learning algorithm, are able to deliver behaviorally triggered stimuli to flies in a feedback-loop mode, and are highly customizable and open source. Ethoscopes can be built easily by using 3D printing technology and rely on Raspberry Pi microcomputers and Arduino boards to provide affordable and flexible hardware. All software and construction specifications are available at http://lab.gilest.ro/ethoscope.

This Community Page is part of the Cool Tools Series.

## Introduction

Understanding how behavior is coordinated by the brain is one of the ultimate goals of neuroscience. In particular, much of modern neurobiology focuses on finding the genes and the neuronal circuits underlying simple and complex behaviors alike, aiming to describe and eventually understand how the brain processes sensory inputs into motor outputs. For many years, starting from Seymour Benzer’s seminal work [1], the fruit fly *Drosophila melanogaster* has been considered one of the model organisms of choice to dissect the genetics of behavior. In the past decade, *Drosophila* has also emerged as an excellent model for studying not only the genes but the neuronal circuitry of behavior too: the combination of a rapidly delineating connectome together with an unrivalled repertoire of genetic tools has established *D*. *melanogaster* as one of the most promising animal models to study neuronal circuits. Optogenetics, thermogenetics, a genome-wide collection of RNA interference (RNAi) lines, and a plethora of crafted and carefully described GAL4 lines, constitute a robust arsenal for neurobiologists interested in studying the neuronal circuitry underpinning behavior. The limiting factor for ethomics—the high-throughput approach to behavioral studies—is therefore not the availability of genetic tools, but rather the access to an objective, reproducible, and scalable system to detect and classify behavior. Historically, *Drosophila* neuroscientists have often shown a high degree of ingenuity in devising paradigms and creating apparatus able to capture relatively simple behaviors in a high-throughput fashion, usually driven by the desire to perform genetic screens. Analysis of phototaxis [2], geotaxis [3], response to ethanol inebriation [4,5], olfactory learning and habituation [6,7], and biology of circadian rhythms [8] are all successful examples of clever paradigms that have allowed high-throughput screenings of specific behaviors. More recently, ad hoc solutions featuring computational approaches have also been introduced: some specifically dedicated to a subset of behaviors, such as sleep [9–11] or feeding [12,13], and others designed to be more versatile [14–17]. Although computer-assisted analysis of behavior has the potential to revolutionize the field, adoption and throughput of currently available techniques are limited by several factors. Predominantly, the requirement for a nonstandardized hardware setup, which often bears problems of cost, footprint, and scalability. Typically, most systems consist of a centralized setup in which 1 or several cameras record high-resolution videos that are then processed, in real-time [9–11] or offline [14,16,18,19], by a central, powerful workstation. To lower entrance barriers to machine analysis of behavior, we developed the ethoscope platform. In devising its architecture—decentralised and modular—we took inspiration from the commercially available Drosophila Activity Monitors (DAMs, TriKinetics Inc., Waltham, Massachusetts), machines that are used routinely by *Drosophila* neuroscientists to study circadian rhythms and sleep. In particular, one of the most successful features of DAMs that we aimed to imitate is the ability to run dozens of experiments simultaneously, gathering data in real-time from thousands of flies at once, using a device that follows a “plug-and-play” approach. Here, we describe the philosophy and technical vision underlying ethoscopes. We provide some examples of raw and processed data that users will be able to acquire and offer some proof-of-principle examples of how ethoscopes can be used for feedback-loop experiments.

## Results

**Fig 1:**
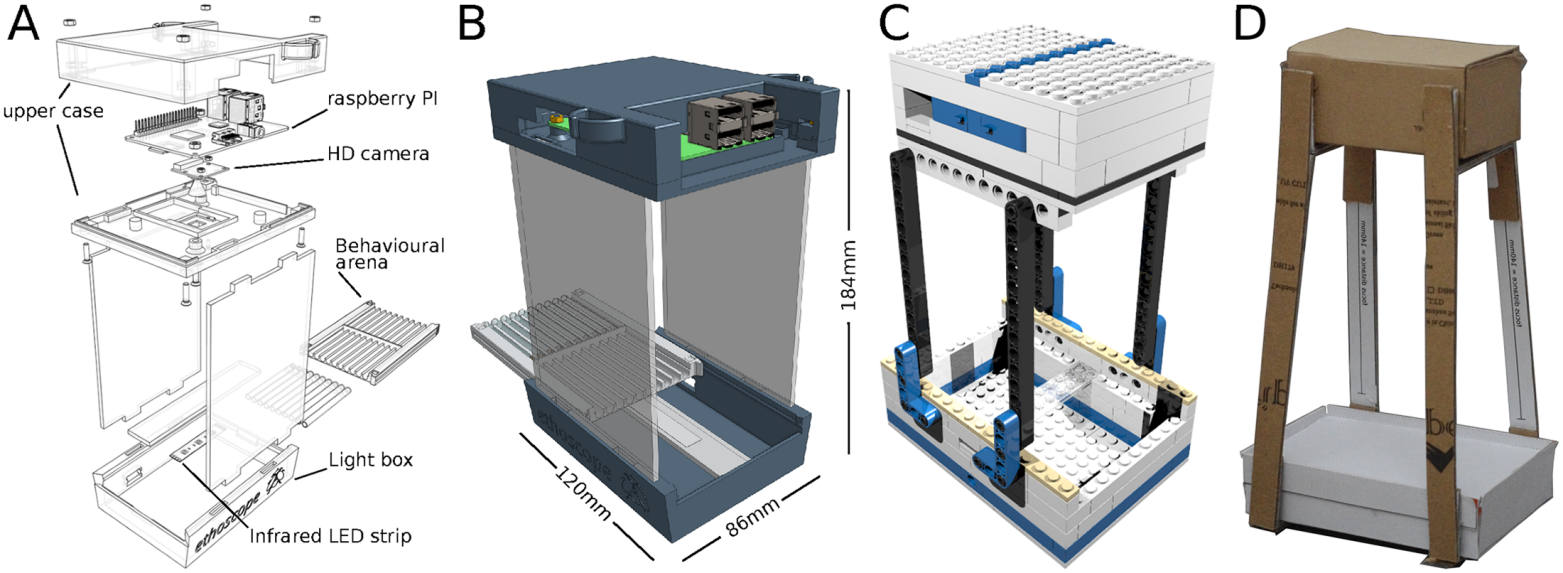
The ethoscope. (A) Exploded drawing of an archetypal ethoscope. The machine is composed of 2 main parts: an upper case housing the rPi and its camera, and a lower case providing diffused infrared light illumination and support for the experimental arena. The 2 cases are separated by spacers maintaining a fixed focal distance (140 mm for rPi camera 1.0). (B) A rendered drawing of the assembled model, showing the actual size without cables. The presence of USB and connection cables will slightly increase the total size (cables not shown for simplicity). The arena slides in place through guides and locks into position. A webGL interactive 3D model is available as S1 Fig. (C) The LEGOscope, a version of the ethoscope built using LEGO bricks. A detailed instruction manual is provided in S1 Text. (D) The PAPERscope, a paper and cardboard version of the ethoscope, best assembled using 220 gsm paper and 1 mm gray board. Blueprints are provided in S2 Text. In all cases, ethoscopes must be powered with a 5 V DC input using a common USB micro cable either connected to the main or to a portable power-pack. DC, direct current; HD, high-definition; LED, light-emitting diode; rPi, Raspberry Pi; USB, universal serial bus.

An ethoscope is a self-contained machine able to either record or detect in real-time the activity of fruit flies (and potentially other animals) using computerised video-tracking. It relies on an independent small single-board computer, Raspberry Pi (rPi) [20], and a high-definition camera (rPi camera [20]) to capture and process infrared-illuminated video up to a resolution of 1,920 x 1,080 pixels, at 30 frames per second (FPS, Fig 1A). Ethoscopes are assembled in a 3D-printed chassis and, with cables, they have an approximate footprint of 10 x 13 x 19 cm (Fig 1B and S1 Fig). Although we recommend a 3D-printed assembly for research-grade use, we also provide detailed instruction to build a fully functional ethoscope out of LEGO bricks (Fig 1C, LEGOscope in S1 Text) or out of folded cardboard (Fig 1D, PAPERscope in S2 Text). These latter 2 options are particularly well suited for the purpose of education and outreach. In all cases, assembly of ethoscopes requires little technical skill. The technical drawings required to 3D print and assemble an ethoscope, along with its software (Python code on a Linux instance) are released under the open source general public license version 3 and are freely available on the ethoscope website (https://lab.gilest.ro/ethoscope). A current version of the user manual, including building instruction, is also provided here as S3 Text, while current snapshots of stereolithography (STL) and image files are also made available on Zenodo [21]. The combination of consumer-grade electronics, 3D printing and free open source software results in a total cost of about €100 for each machine. Software is provided as source on a Git repository and as self-contained images that can be written either on secure digital (SD) cards to fit inside each rPi, or on a CD to work as the controlling unit (“the node”). Limited cost, combined with each ethoscope relying on its own computing power, allows for easy scaling of the entire platform.

**Fig 2:**
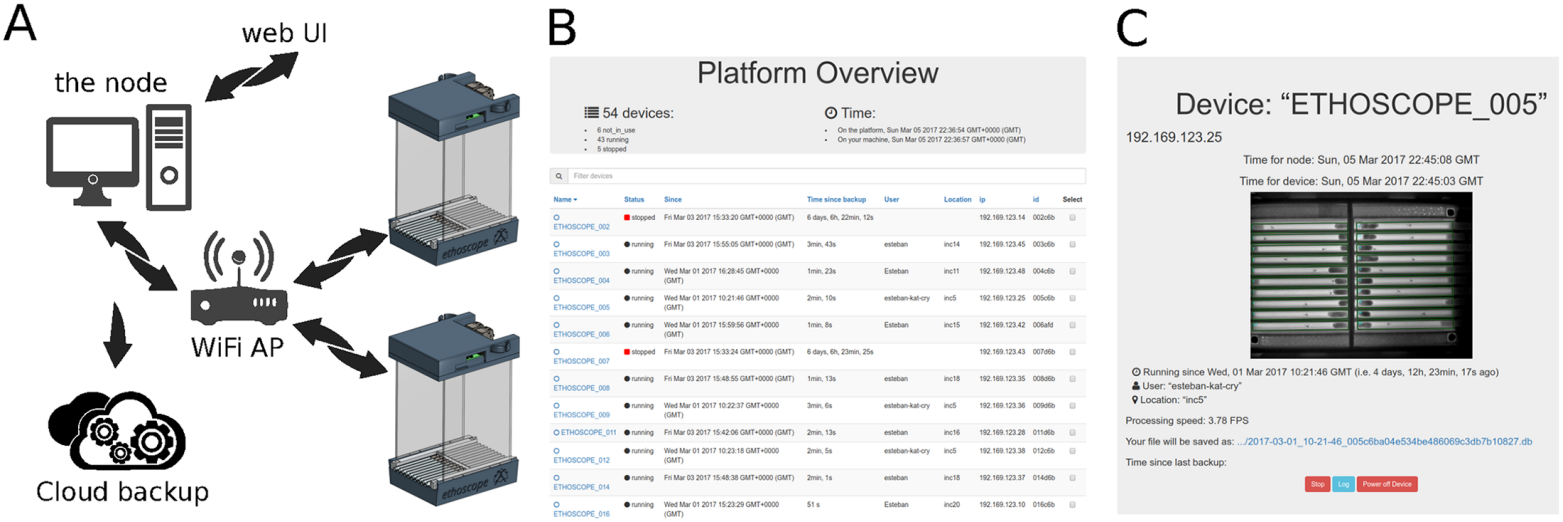
The ethoscope platform. (A) A diagram of the typical setup. Ethoscopes, powered through a USB adapter, are connected in an intranet mesh through an AP or a Wi-Fi router. A computer in the network acts as the node, receiving data from ethoscopes and serving a web-UI through which ethoscopes can be controlled, either locally or remotely. (B) Screenshot of the homepage of the web-UI, showing a list of running machines and some associated experimental metadata (e.g., username and location). (C) Screenshot of an ethoscope control page on the web-UI, providing metadata about the experiment and a real-time updated snapshot from the ethoscope point of view. AP, access point; GMT, Greenwich mean time; FPS, frames per second; USB, universal serial bus; web UI, web-based user interface.

In a typical usage scenario, several ethoscopes are placed in a climate-controlled chamber. Each ethoscope is powered through a universal serial bus (USB) cable and communicates via Wi-Fi to a local network, uploading data to a desktop computer acting as the data collecting station (the node in Fig 2A). Through the same network, ethoscopes can be remotely commanded using a graphical web interface (Fig 2B and 2C and S1 Movie). If the node is connected to the Internet, the entire platform will receive automatic software updates from the upstream Git repository. Because each ethoscope operates independently, there is no theoretical limit to the number of machines that can be used concurrently. In fact, the ability to run dozens of ethoscopes simultaneously is one of the crowning features of the system. However, rPis are quad-core microcomputers that generate considerable heat under heavy computing load. For this reason, the use of a climate-controlled chamber is a strict requirement and remains the greatest limitation of the platform at present. In our laboratory, we run up to 70 ethoscopes at once—analyzing 1,400 flies—spread across 20 commercial wine coolers modified to be used as temperature-controlled chambers (details of the modifications are available upon request). Besides being a good solution for multiuser environments, the use of many small climate chambers, rather than a few with greater capacity, also allows for more flexibility in designing and running experiments; for instance, by running different cohorts at different temperatures for thermogenetic manipulation, or by running different time zones in the same room. Importantly, provided animals have access to fresh food, the platform is able to run experiments for weeks. Ethoscopes connected to the network will periodically transfer data to the node acting as a local storage server, ensuring experimental duration is not limited by the storage capabilities of the rPi.

**Fig 3:**
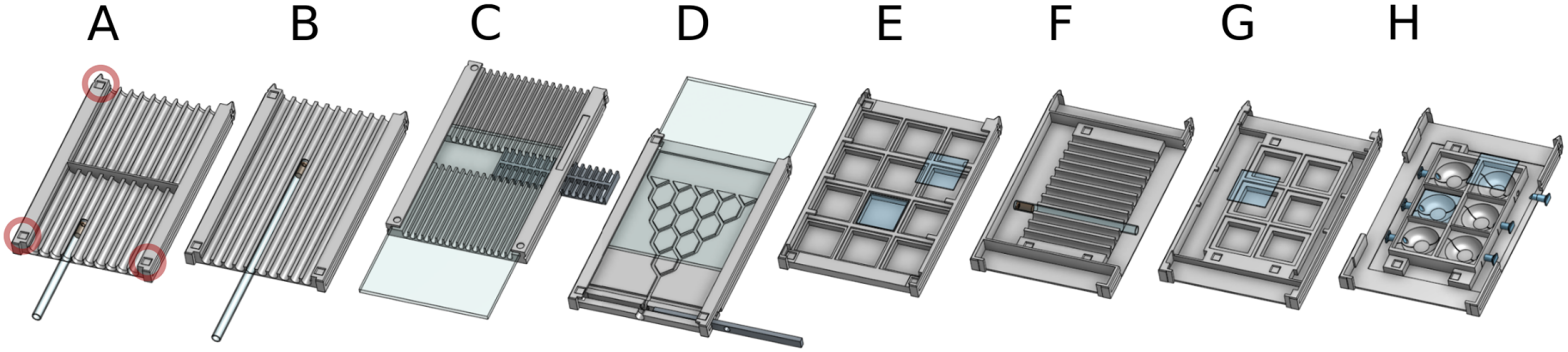
Versatility of use with custom behavioral arenas. (A-H) Examples of 8 different behavioral arenas whose files for 3D printing are available on the ethoscope website. (A) Sleep arena. Most commonly used arena in our laboratory for sleep studies, lodging 20 individual tubes. (B) Long tubes arena. It houses 13-cm tubes and can be used for odor delivery studies or, more generally, for behaviors requiring more space. (C) Food bullet arena. Animals are placed directly on the arena and food can be replaced by pushing in a new bullet [11]. It does not require glass tubes and can be used for quick administration of chemicals in the food. (D) Decision making arena. It can be used to study simple decision making behaviors, adapted from Hirsch [3]. (E) Square wells arena. It can be used for courtship assay or to record activity in a bidimensional environment. (F, G) Conceptually analogous to A and I, but designed to work in high-resolution (full-HD) settings. (H) Round wells arena, modelled following specifications from Simon and Dickinson [22]. Note that all arenas are marked with 3 visible reference points (indicated by a red circle in A) that are used by the ethoscope to automatically define regions of interest for tracking. HD, high-definition.

The experimental flies are loaded into a behavioral arena that slides and locks inside the lower part of the ethoscope chassis (Fig 1A). Like the rest of the machine, arenas are 3D printed and their design depends on the nature of the experiment. Some examples of arenas inspired by commonly used behavioral paradigms are provided in Fig 3 and span arenas adopted for long-term experiments that may last for weeks, such as sleep or longevity analysis (Fig 3A–3C and 3F), or short-term assays such as decision-making (Fig 3D) and courtship (Fig 3E, 3G and 3H). All arenas feature 3 fixed recognition marks on the corners (red circles on Fig 3A) that are used by ethoscopes to automatically align and register the regions of interest for tracking. When starting an experiment, the experimenter can decide whether the activity of the animals should be tracked in real-time or whether the ethoscope should record a video to be analyzed offline, with the ethoscope software or with other software, such as the C-trax/JAABA suite [14,15], CADABRA [17], or idTracker [16]. In real-time tracking mode, ethoscopes will detect and record the position and angle of each animal with a variable frame rate that fluctuates between 1 and 4 FPS, depending on the computing load (e.g., the number of flies to be tracked and the number of regions of interest; see S4 Text for technical details of real-time tracking and its performance).

**Fig 4:**
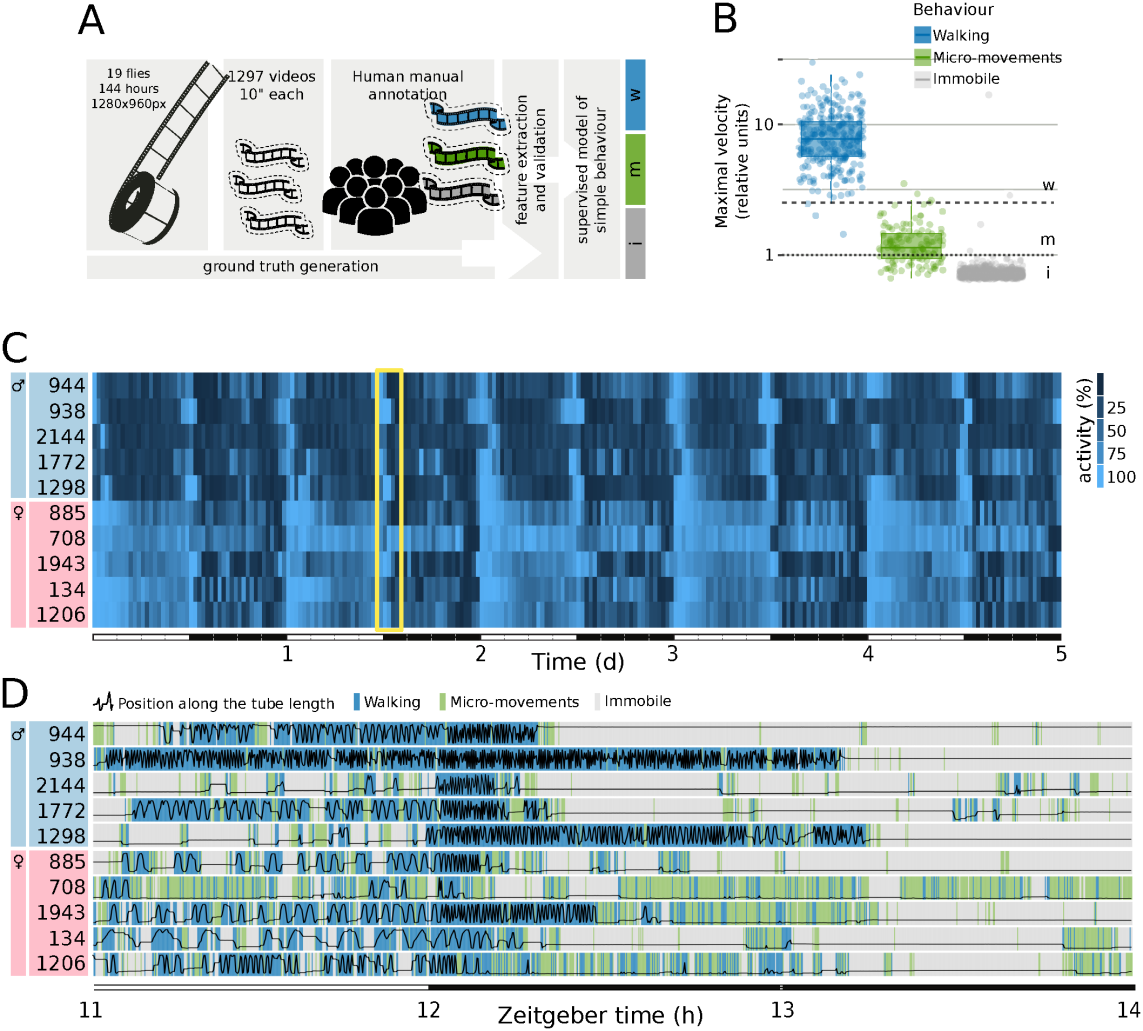
Tracking and validation of behavioral classification. (A) To build a statistical model of activity, we used ethoscopes to record offline 2,736 hours of video (144 hours x 19 flies) at resolution of 1,280 x 960 pixels and frame rate of 25 FPS. Video fragments of the duration of 10 seconds were sampled every hour for all 19 animals and scored by at least 3 experienced fly researchers in a randomized order. Consensual annotations—where majority of scorers agreed—were kept, resulting in a ground truth of 1,297 video fragments (116 ambiguous annotations were excluded by using this latter criteria). Scorers manually annotated both the position of the animal in the tube and the perceived behavioral state (i.e., immobile, micromoving, or walking). Ethoscope video tracking was run independently on the whole video down-sampled between 1 and 5 FPS, all realistic frame rates for real-time analysis. (B) Distribution of corrected maximal velocity (relative unit, see S4 Text) for each behavior, showing the thresholds used to detect movement (1: dotted line) and walking (2, 5: dashed line). (C) Five days’ recording of activity of 10 representative flies: 5 males (cyan boxes) and 5 females (rose boxes). Flies were kept in a regime of constant climate in a 12 hour:12 hour light-dark cycle (as indicated by the lower bar alternating white and black). The yellow frame highlights the 3-hour window shown in D. (D) Detailed activity for the same individuals shown in C, during a 3-hour window spanning a light to dark transition. The black line shows the position of the animals from the food end to other extremity of the tube (bottom to top). The background colors highlight the behavioral features as detected in real-time by the ethoscope, with a definition of 10 seconds per pixel (same legend as B). FPS, frames per second, px, pixel.

The ethoscope software is modular in design, meaning many components can be replaced or adapted as needed. The tracking module is one that the end users may want to adapt to their needs ultimately. Currently, we provide the following 2 tracking options: an adaptive background subtraction model (default option, S4 Text) and an experimental tracking module based on haar-cascades [23], which is suitable for tracking multiple animals in the same region of interest without maintaining their identities. To validate the accuracy of the default tracking mode, we asked 3 experienced fly researchers to manually annotate the position of the flies in 1,413 still frames extracted from 2,736 hours of recorded videos. We then compared the manually annotated positions to the coordinates of the fly centroids as detected by the ethoscope tracking software, and found a strong degree of overlap, with a median discrepancy of 300 μm, corresponding to a tenth of a fly body length. In no cases (0/1,413 frames), did the error exceed one body length (2.5 mm). To enrich the capabilities of ethoscopes, we also implemented a real-time behavioral annotator. We created a ground-truth of 1,297 videos, each lasting 10 seconds and each manually annotated by at least 3 experienced fly researchers (Fig 4A, annotation labels were: “walking,” “micro-movement,” or “immobile”). Random forest variable importance [24] was used to screen for predictors of movement in a supervised manner and the 2 highest-ranking features—maximal velocity and cumulative walked distance—were selected for further analysis. Conveniently, maximal velocity alone appeared to serve as a faithful predictor of behavior (Fig 4B) allowing for real-time dissection of basic behavior. Therefore, not only can ethoscopes reliably annotate the position of flies but they can also detect when an animal is immobile, performing a micromovement (such as grooming, eating, or egg laying), or walking, with an accuracy of 94.3% for micromovement detection and 99.0% for walking detection. As proof of principle, we show a low resolution (5 days with a definition of 30 minutes, Fig 4C) and a high resolution (3 hours with a definition of 10 seconds, Fig 4D) activity plot for 10 individual animals (5 young males and 5 young females, between 4 and 9 days old).

**Fig 5:**
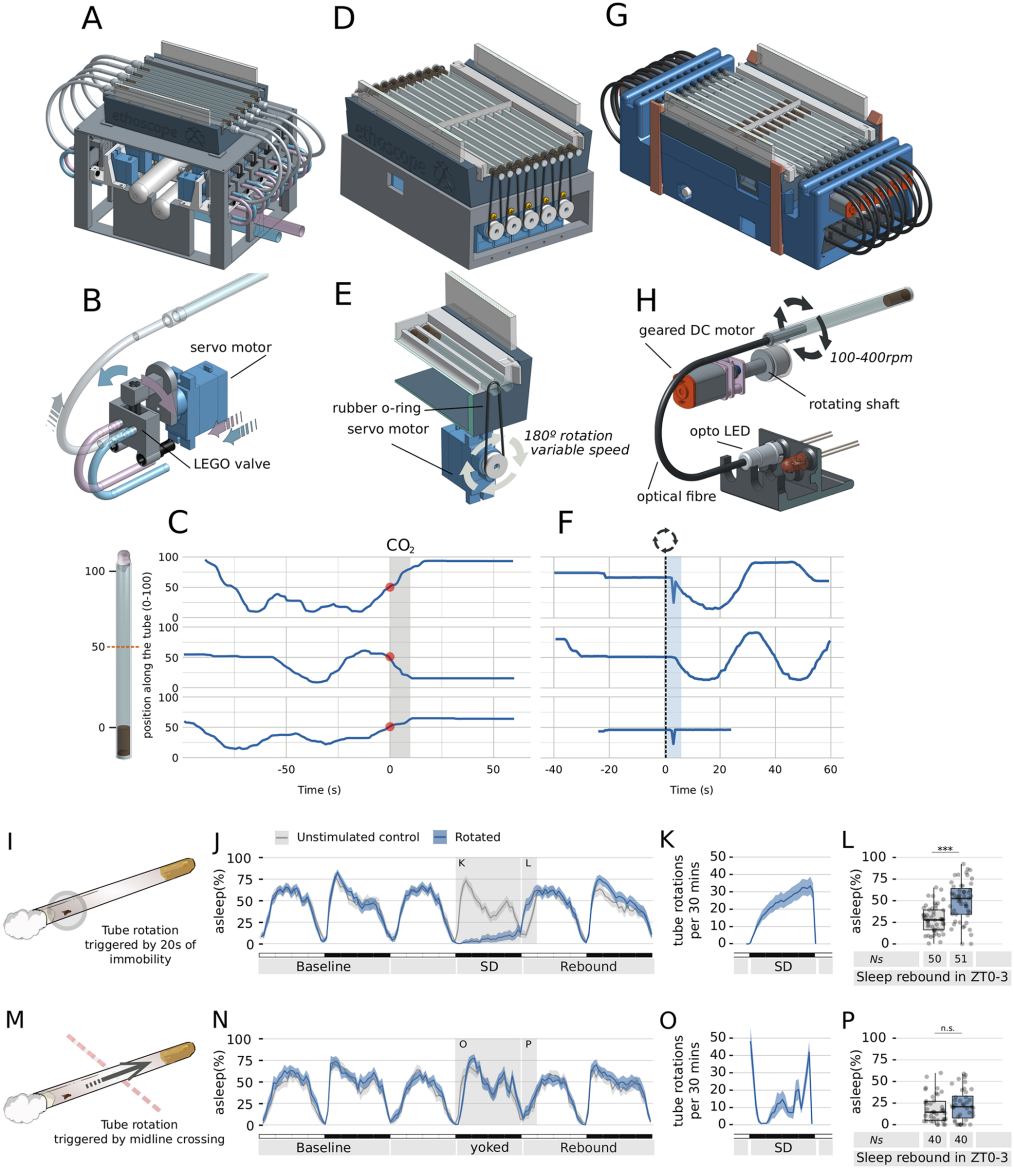
Versatility of use with behavioral feedback-loop modules. (A) Diagram and (B) detail of the AGO-delivery module. Two independent flows (blue and purple in the drawing) are fed into the module using external sources. The module features 10 LEGO pneumatic valves, each independently controlled through a servo motor. The motor switches the air source on the valve, selecting which source will be relayed to the tube containing the fly. Available positions are blue source, purple source, and closed. (C) Representative response of 3 flies subjected to CO_2_ administration using the AGO module. CO_2_ release lasts 5 seconds (grey bar) and it is triggered by midline crossing (red dot). The blue line indicates the fly position in the tube over the 150 second period. (D) Model and (E) detail of the rotational module. The module employs a servo motor to turn the tube hosting the fly. The direction, speed, duration, and angle of the rotation can be modulated to change the quality of the stimulus. (F) Representative response of 3 flies upon stimulation using the rotational module shown in (D, E). Rotation of the tube is triggered by 20 consecutive seconds of immobility (dashed line) and is followed by 5 seconds of masking, during which tracking is suspended to avoid motion artefacts (cyan area). The bottom panel shows traces of a dead fly. (G) Model of the optomotor module able to simultaneously stimulate single flies with rotational motion and light. (H) Detailed view of the optomotor minimal unit. Light is directed into the tube using optical fiber. S2 Movie shows the optomotor module in action. (I-P) The servo module employed for a sleep deprivation experiment. Flies shown in grey are unstimulated mock controls, never experiencing tube rotations. Flies shown in light blue experience rotation either after 20 seconds of inactivity (I-L) or after midline crossing (M-P). (J, N) Sleep profile of flies along 3 days in conditions of 12 hour:12 hour, light and dark cycles. Gray shadings indicated the stimulation period and the following sleep rebound period. (K, O) Number of tube rotations delivered during the 12-hour stimulation period. (L, P) Quantification of sleep rebound during the first 3 hours of the day following the stimulation. AGO, air/gas/odor; DC, direct current; LED, light-emitting diode; rpm, revolutions per minute; SD, secure digital; ZT, Zeitgeber time.

The ability to operate in real-time offers a crucial feature: delivering animal-specific feedback-loop stimuli following a predefined behavioral trigger. Interfering with the behavior of an animal through external stimuli is an important tool for neuroscientists. In principle, feedback loops can be used for multiple purposes, such as to reinforce learning, to interrupt sleep, to stimulate or silence circuits using optogenetics, to study operant conditioning, etc. Systems operating feedback-loop stimuli on fruit flies have been proposed previously and have already proved to be instrumental, but are not easily compatible with a high-throughput approach and are focused on very specific usage [25,26]. Ethoscopes can be extended with modules that seamlessly connect with the machine and react in real-time to trigger an action whenever a condition is satisfied. Fig 5 demonstrates 3 examples of such modules: an air/gas/odor (AGO) delivery module (Fig 5A and 5B), a rotational module (Fig 5D and 5E), and an “optomotor” module combining optogenetic stimulation and motor disturbance (Fig 5G and 5H). All modules plug into the bottom part of the machine and are configured through the main graphical web-interface, in which the experimenter can set the trigger conditions that will activate the stimulus and schedule a time window for their function (S1 Movie). A trigger can be a combinatorial ensemble of position, time, and behavior (e.g., “micromovement for at least 20 seconds within 5 mm from the food” or “immobile for at least 5 minutes anywhere”). As proof of principle, we provide representative evidence of how individual flies react to the following 3 different stimuli: a 5 second delivery of CO_2_, triggered by crossing the midline of the tube (Fig 5C); a 2 second fast rotation of the tube (60°/0.12 seconds), triggered by 20 seconds of immobility (Fig 5F); a 5 second optostimulation on moon-walker [27] receptive flies, manually or automatically triggered (S2 Movie). We also provide a case test for using the rotation module as a sleep deprivation device (Fig 5I–5P). To this date, scientists studying sleep in flies have the option of performing mechanical sleep deprivation by placing animals on an orbital shaker [28], a rotating device [29], or a vibrating platform [10]. In all cases, the resulting mechanical stimulation of the animals is independent of their actual activity, so that the stimulus is delivered unspecifically to all individuals at the same time (i.e., to some while asleep and to others while awake). Using this module, we can rotate single tubes—hence, individual animals—only when a fly is immobile (e.g., after 20 consecutive seconds of immobility, Fig 5I–5L) or, in the yoked control, only when a fly is actually walking but not eating or grooming (e.g., after midline crossing, Fig 5M–5P). A conceptually identical paradigm was originally introduced in the 1980s [30], and it is still considered one of the best controlled paradigms for chronic sleep deprivation of rodents. As shown in Fig 5, all flies were subjected to an analogous number of tube rotations (548 ± 342 for experimental sleep deprivation; 383 ± 173 for yoked control; mean ± SD; temporal pattern shown in Fig 5K and 5O), but only the experimental sleep deprivation led to a sleep rebound after the treatment (Fig 5L and 5P), thus confirming that sleep rebound is indeed a specific countereffect of sleep deprivation [31]. For sleep scientists, the possibility to precisely interrupt the sleep of flies may be a crucial tool to differentiate the effects of mere sleep deprivation from the effects of stress, 2 confounded phenomena [29]. On the ethoscope website, we provide detailed instruction on how to build all 3 modules in conjunction with a description of the API needed to interface any new custom module to the ethoscope platform.

## Discussion

Ethoscopes emerge from the maker culture and combine 3 important innovations of the last few decades—3D printing, small single-board computers, and machine learning—into a novel tool for behavioral researchers. They were designed to be easy to build, inexpensive, and compatible with high-throughput research. Accessibility and high-throughput design are certainly 2 important features of the platform, but we anticipate that the combination of those 2 with the ability to create custom feedback-loop experiments will make ethoscopes particularly useful for the community. Creating feedback-loop based experiments is something that *Drosophila* neuroscientists have been doing for decades with great ingenuity and success [25, 32–34]. However, these generally require ad hoc equipment and provide limited procedural throughput. Ethoscopes build upon this tradition, but offer a modular platform that may simplify this procedure and favor wide adoption.

The philosophy of distributed microcomputing is one of the strongest features of the ethoscope platform—in terms of affordability and scalability—but at the same time it constitutes its current greatest weakness: relatively limited computational power. In their current form, ethoscopes rely on rPis and work best when sporting rPi version 3 (rPi 3), their most powerful hardware. In principle, however, any microcomputer platform able to connect to a camera would work, and it is possible that future versions may take advantage of commercial development to improve computational power and, ultimately, performance. As of now, real-time tracking is limited to a temporal resolution of 1–4 Hz. Whenever greater temporal resolution is needed, the offline tracking mode transforms ethoscopes into remotely controlled video cameras and allows users to acquire video files at up to 90 FPS to be analyzed at a later stage with another software of choice. If greater spatial resolution is needed, it is possible to expand the rPi cameras with lenses featuring an M12 mount. The possibility of coupling rPi cameras to lenses has been demonstrated recently by the FlyPi project, a tool very similar in philosophy but different in scope [35]. The fruit fly community has produced excellent software for automatic recognition of complex behaviors [14, 16, 36] with the demonstrated potential of revolutionizing the field [37]. Ethoscopes can contribute and assist to this end by facilitating scalability.

We anticipate that one of the most interesting developments of the platform may be the growing variety of feedback loop modules. Here, we offered 3 examples of such modules that can be used to expand ethoscopes’ abilities. We expect and encourage users to build modules based on their own needs, increasing the available range of modules. For instance, scientists studying feeding behavior may want to create a new module able to simultaneously record access to food by using either the expresso [13] or flyPAD[12] technologies. Collimated high-power light-emitting diodes (LEDs) coupled to small optical filters could also be used to create a module for visualisation of immunofluorescence in real time [35]. Another possible future improvement may derive from the announced introduction of machine-learning dedicated chips (tensor processing units [TPUs]), which are currently being developed by tech giants such as Google, Microsoft, and NVidia. It is likely that future versions of microcomputers will possess some form of TPUs, and that may allow for a more powerful discrimination between behaviors in real time.

Another possible use of ethoscopes is the adaptation of the platform to detect behavior of other animals. Clearly, adapting ethoscopes to work with other small insects similar to *Drosophila* should be an easy task; tracking behavior of even smaller animals may be possible using lenses, and modified illumination techniques such as frustrated total internal reflection [FTIR] for tracking the behavior of larvae or worms [38–40].

## Methods

### Model design and 3D printing

All parts were designed using the web SaaS onshape (http://www.onshape.com). All components were printed using Ultimakers 2+ (Ultimaker, Geldermailsen, Netherlands), with 2.85-mm PLA filament (RS 832–0273). The STL to gCode translation was achieved using the Ultimaker software, Cura (https://github.com/Ultimaker/Cura).

### LEGOscope

The LEGOscope brochure was created using the LEOCad software (http://www.leocad.org/). Please note that LEGO is a trademark of the LEGO group, which is not involved with ethoscopes in any way.

### Electronics

Electronic components were obtained through RS Components, UK and Farnell, UK. A complete up-to-date bill of materials is available on the ethoscope website and in S3 Text.

### Data analysis and statistics

All data analysis was performed in R [41], using the Rethomics R package (https://github.com/gilestrolab/rethomics), and statistical analysis (Fig 5L and 5P) consisted of pairwise Wilcoxon rank sum test (i.e., Mann-Whitney U test). For the sleep plots (Fig 5J, 5K, 5N and 5O), bootstrap resampling with 5,000 replicates was performed in order to generate a 95% confidence interval [42] (shadowed ribbons around the mean in the figures). “N” indicates the total number of flies overall in the experiments. Statistics were performed on aggregated data. Outliers were never excluded. Flies that died during the course of the experiment were excluded from all analysis. Traces and plots were generated in R, using ggplot2 [43]. For all the boxplots, the bottom and top of the box (hinges) show the first and third quartiles, respectively. The horizontal line inside the box is the second quartile (median). Tuckey's rule (the default) was used to draw the whiskers (vertical lines): the whiskers extend to last extreme values within ±1.5 IQR from the hinges, where IQR is Q3−Q1.

## Supporting information

S1 Fig Interactive 3D rendering of the assembled ethoscope—Requires a web graphics library capable browser (e.g., Google Chrome). (HTML)

S1 Text Instruction booklet for building a LEGOscope. (PDF)

S2 Text Instruction booklet for building a PAPERscope. (PDF)

S3 Text User manual and instruction manual for the ethoscope. (PDF)

S4 Text Technical description of the tracking algorithm. (PDF)

S1 Movie An overview of how the ethoscope platform works. (MP4)

S2 Movie The optogenetics component of the optomotor module in action. Moonwalking flies (VT50660-Gal4:: UAS-CsChrimson) are illuminated for 5 to 7 seconds using a red LED (630 nm) through an optical fiber. Illumination is either manually triggered (first part of the video), or triggered by the fly position. LED, light-emitting diode.
(MP4)

## Abbreviations

AGO: air/gas/odor
AP: access point
DAM: Drosophilia Activity Monitor
DC: direct current
FPS: frames per second
FTIR: frustrated total internal reflection
GMT: Greenwich mean time
HD: high-definition
LED: light-emitting diode
px: pixel
RNAi: RNA interference
rPi 3: raspberry Pi, third version
rPi: raspberry Pi
rpm: revolutions per minute
SD: secure digital
STL: stereolithography
TPU: tensor processing unit
USB: universal serial bus
web-UI: web-based user interface
ZT: Zeitgeber time
