# Supplementary material for: Ethoscopes: An open platform for high-throughput ethomics
Source: PLoS Biol. 2017 Oct 19;15(10):e2003026. doi: 10.1371/journal.pbio.2003026 (PMC5648103; doi:10.1371/journal.pbio.2003026)
Supplement: S1 Fig — (HTML) [file pbio.2003026.s001.html]

# Loading Please wait...
