# Supplementary material for: Ethoscopes: An open platform for high-throughput ethomics
Source: PLoS Biol. 2017 Oct 19;15(10):e2003026. doi: 10.1371/journal.pbio.2003026 (PMC5648103; doi:10.1371/journal.pbio.2003026)
Supplement: S1 Text — (PDF) [file pbio.2003026.s002.pdf]

ETHOSCOPE

HOW TO BUILD AN ETHOSCOPE OUT OF LEGO BRICKS

5 - 99

lab.gilest.ro/ethoscope

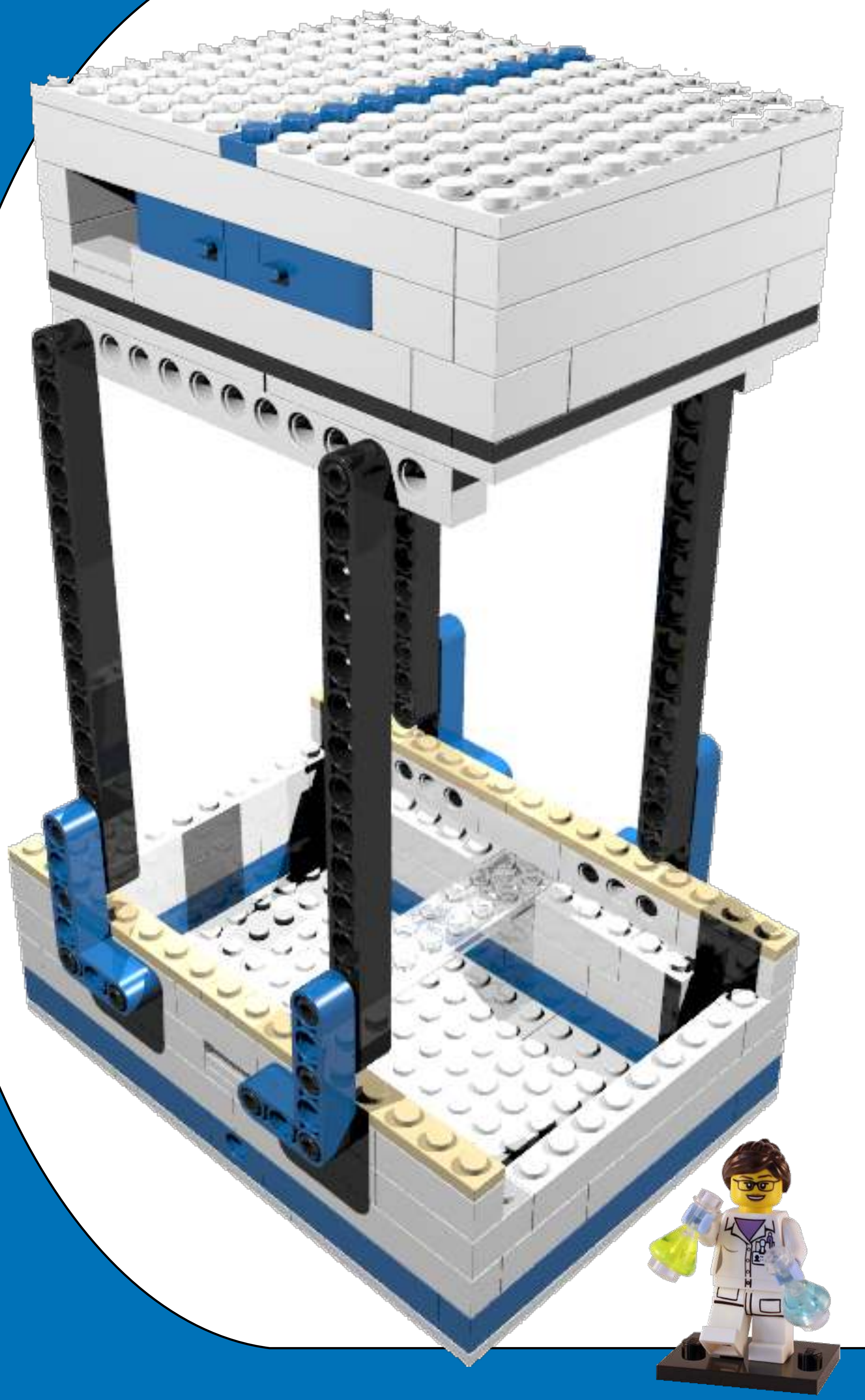

1

1

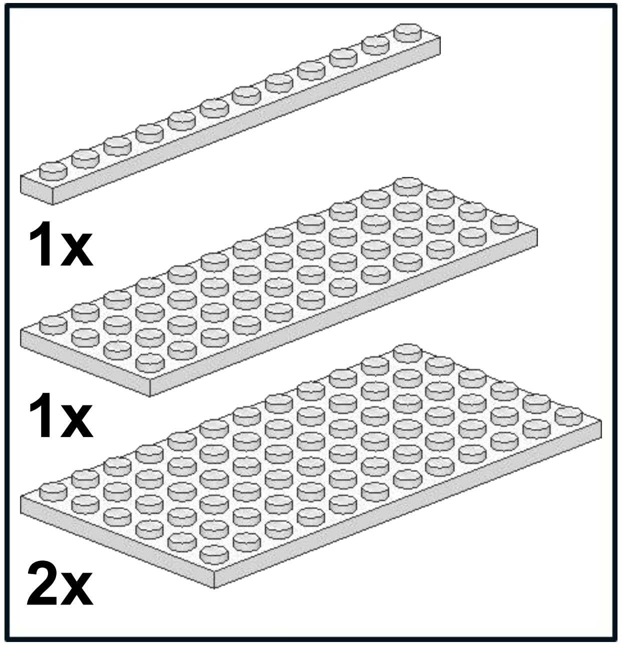

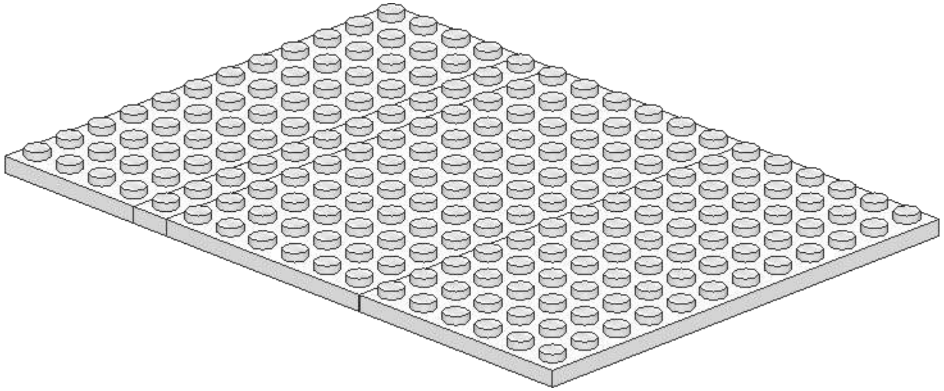

2

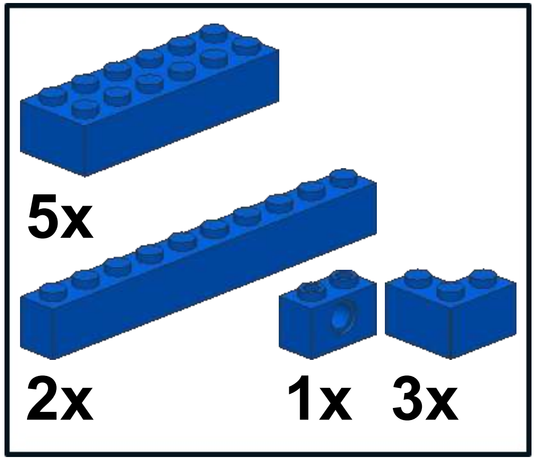

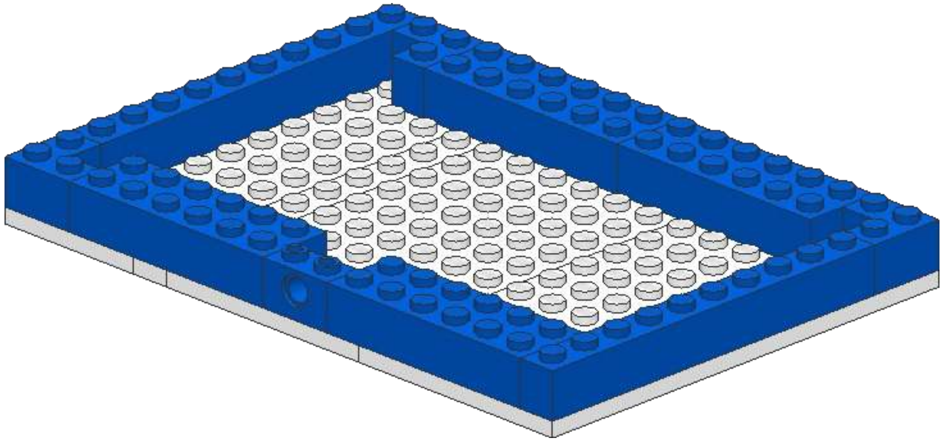

3

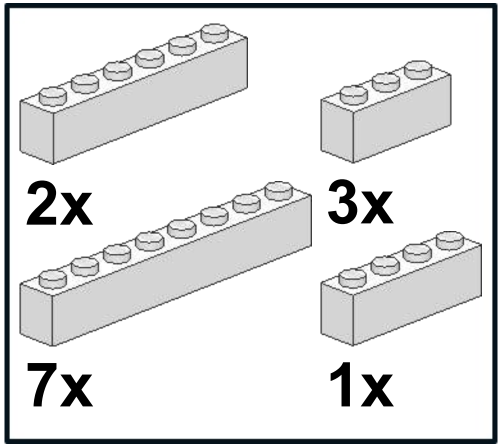

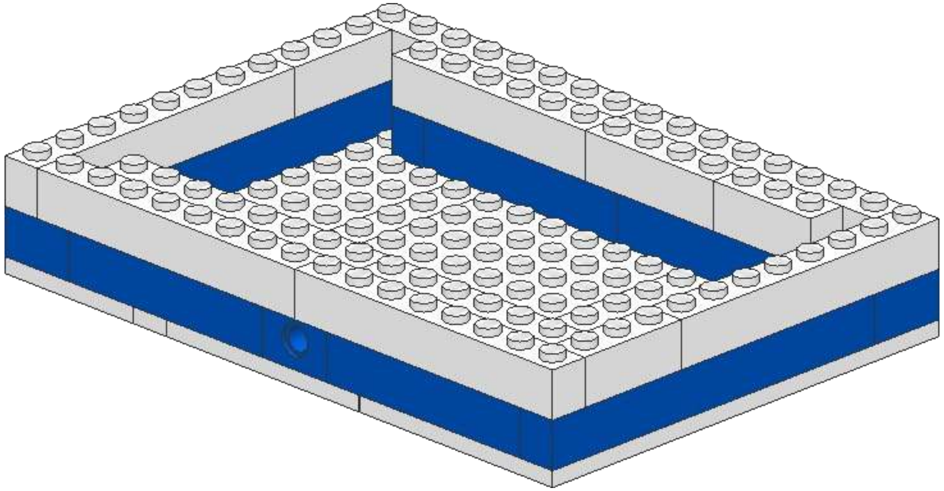

4

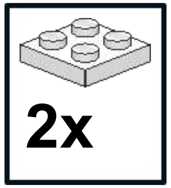

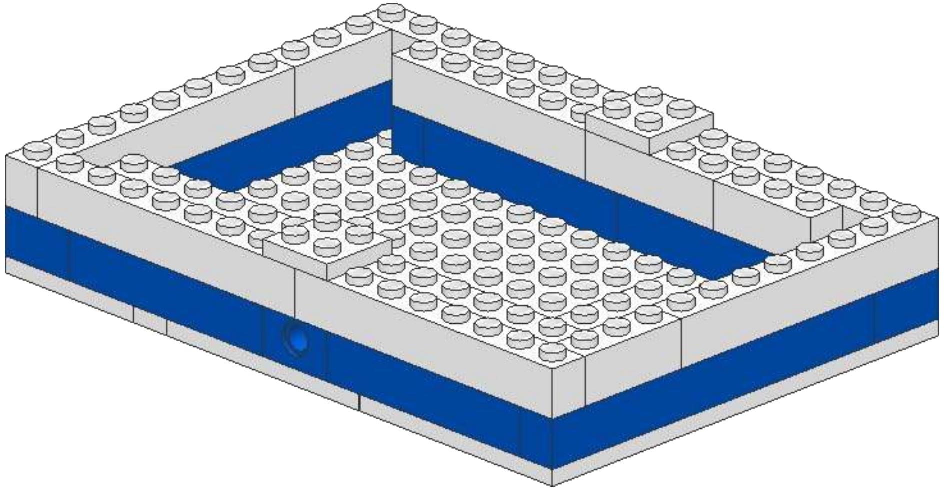

5

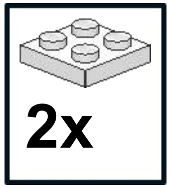

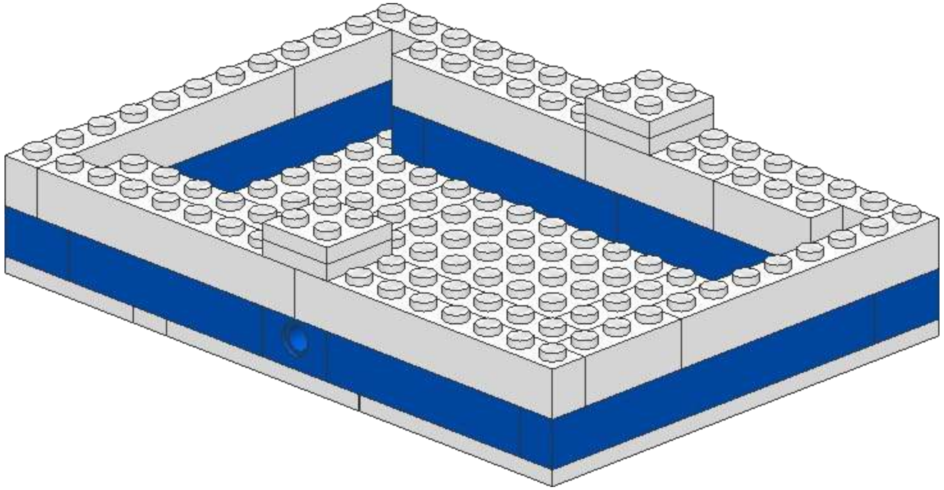

6

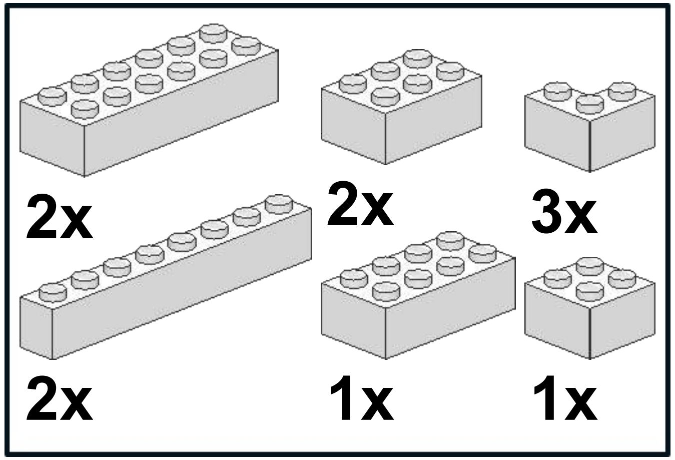

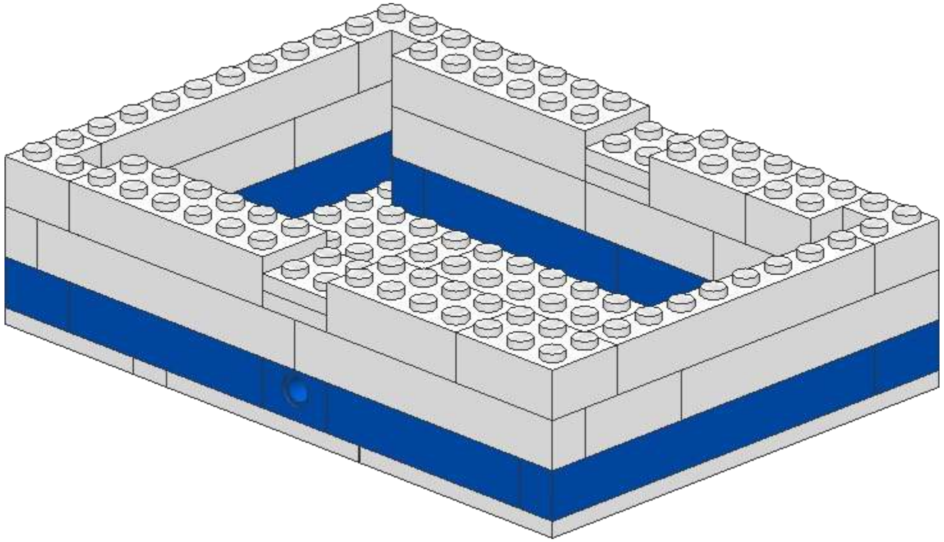

7

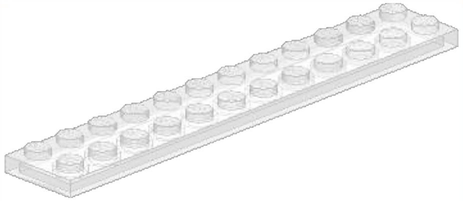

1x

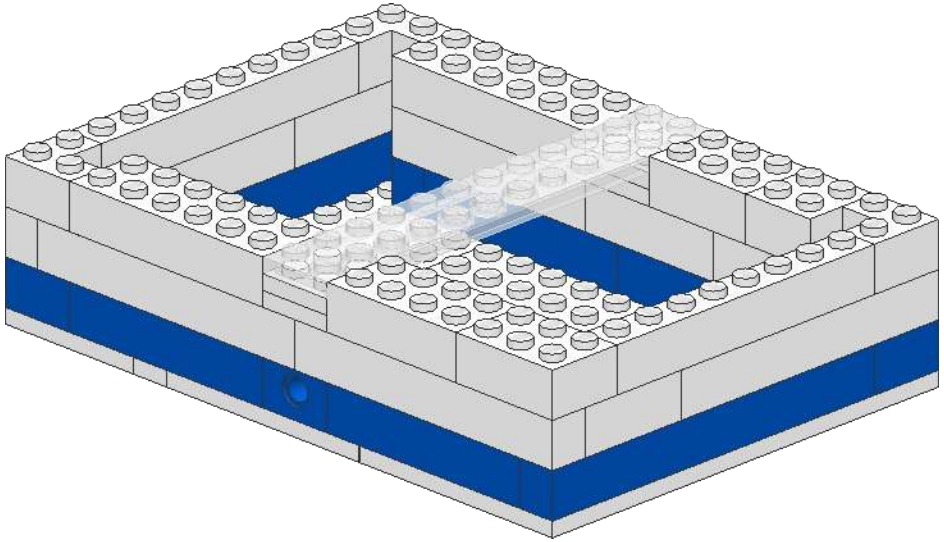

8

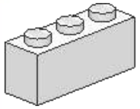

6x

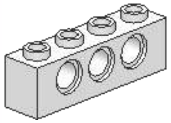

4x

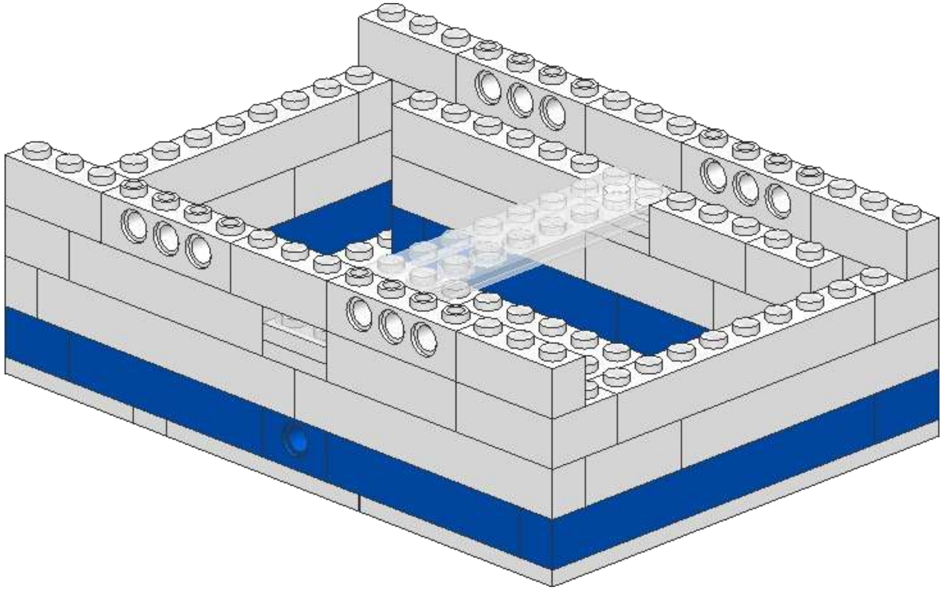

9

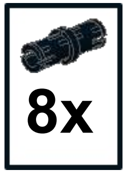

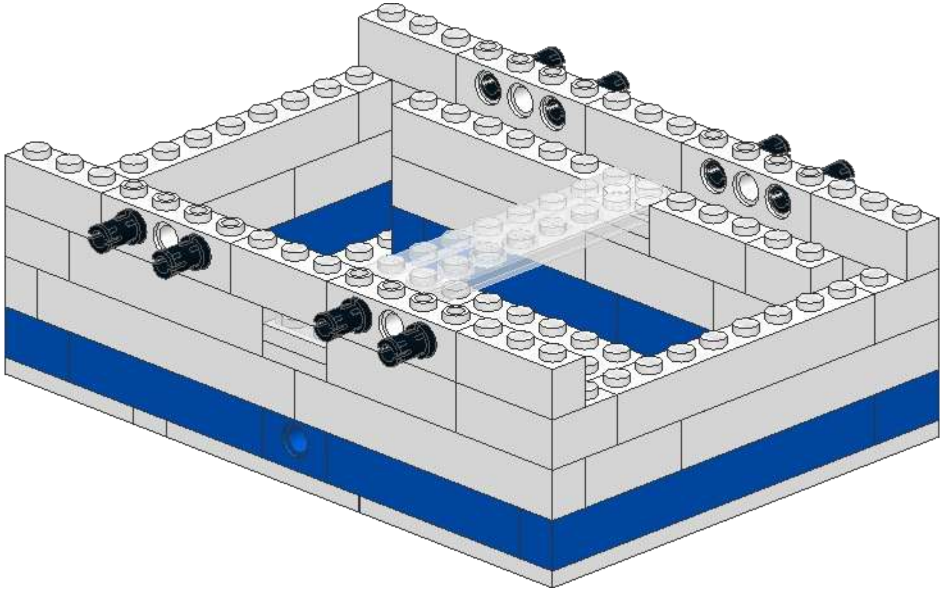

10

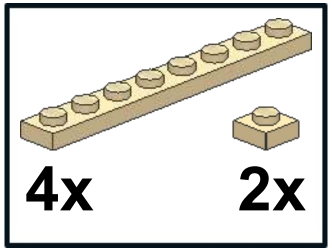

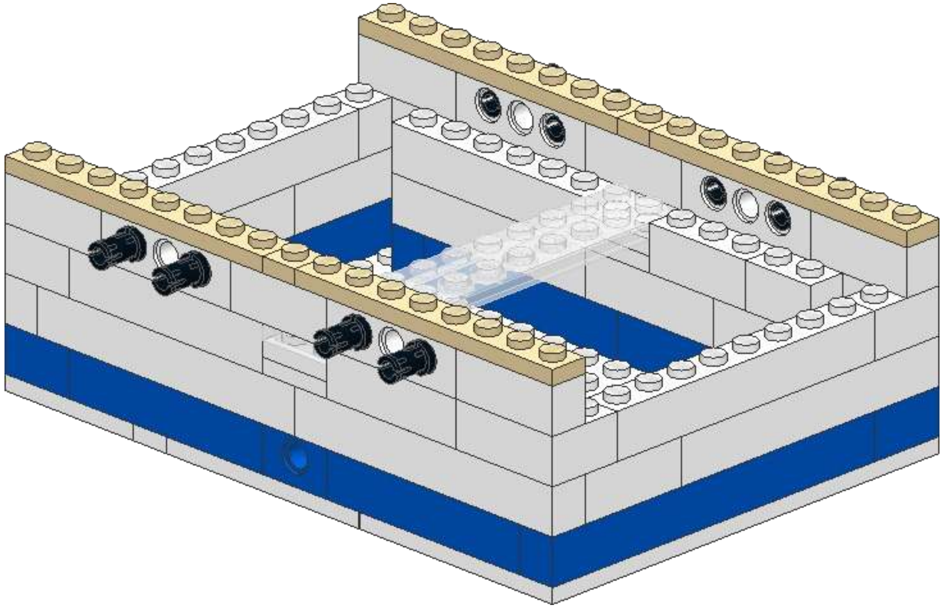

11

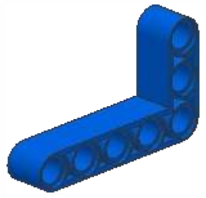
4x

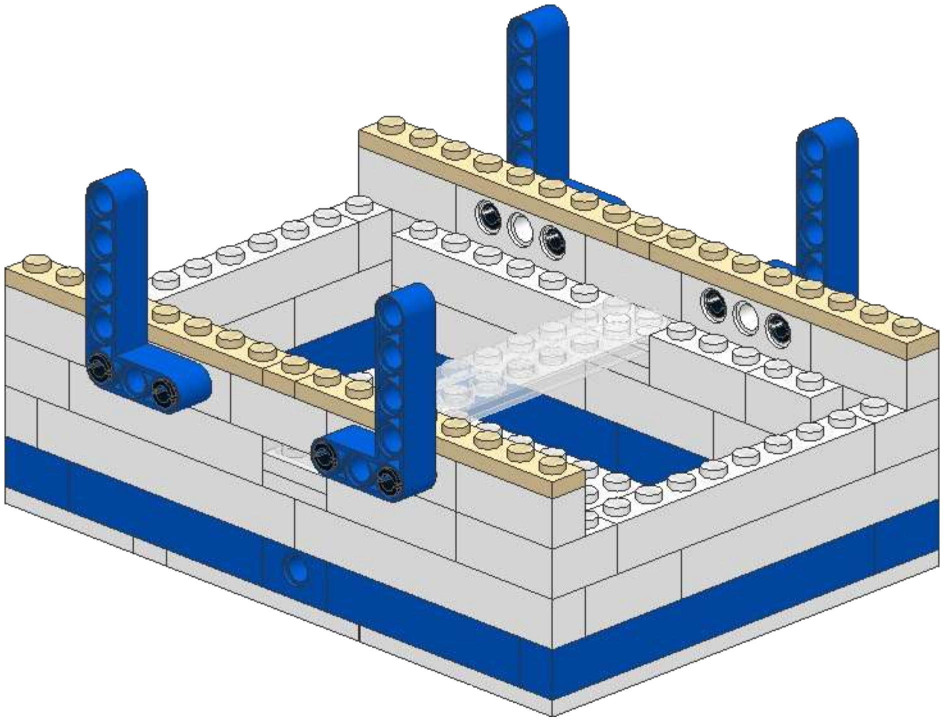

12

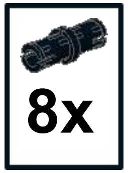

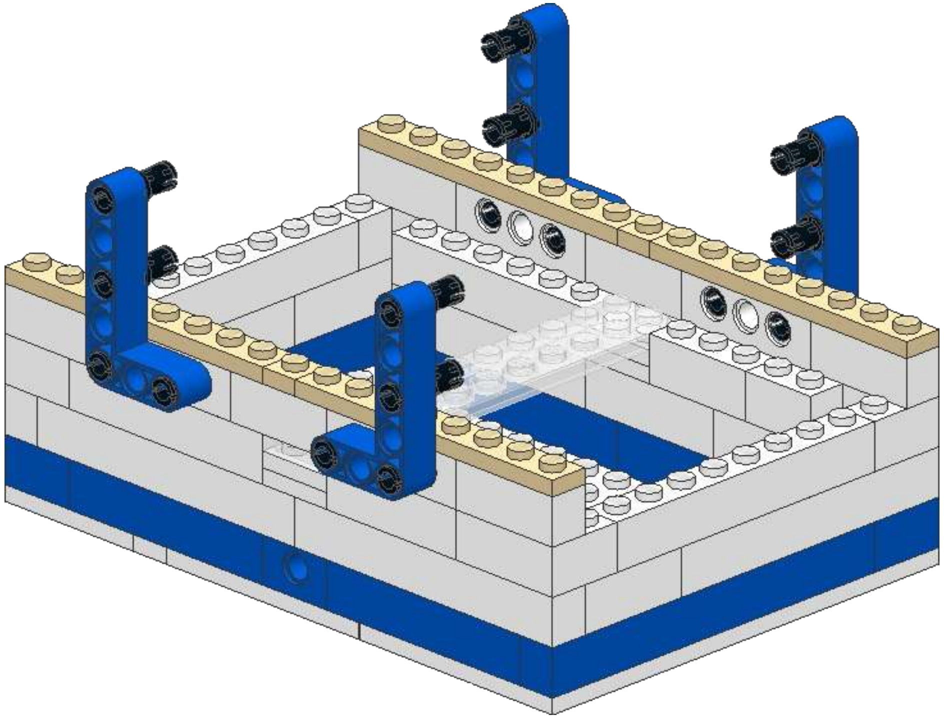

13

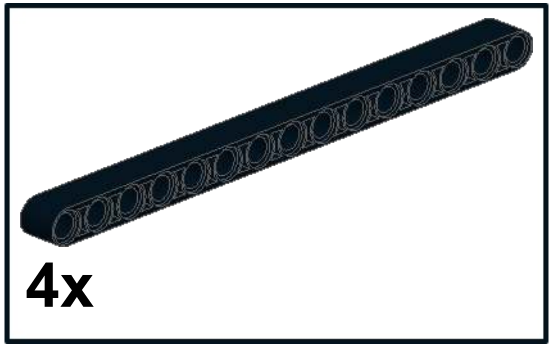

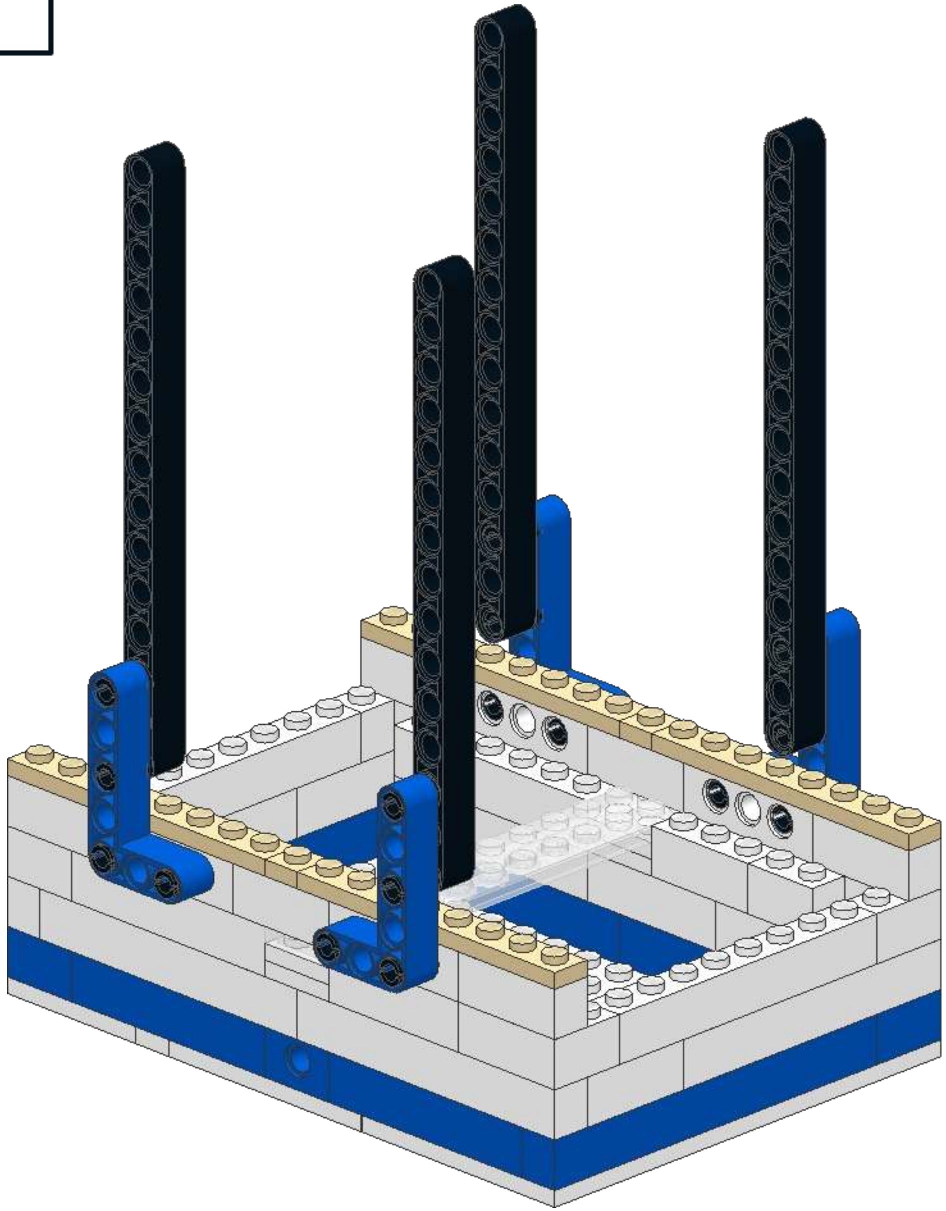

14

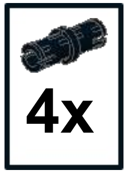

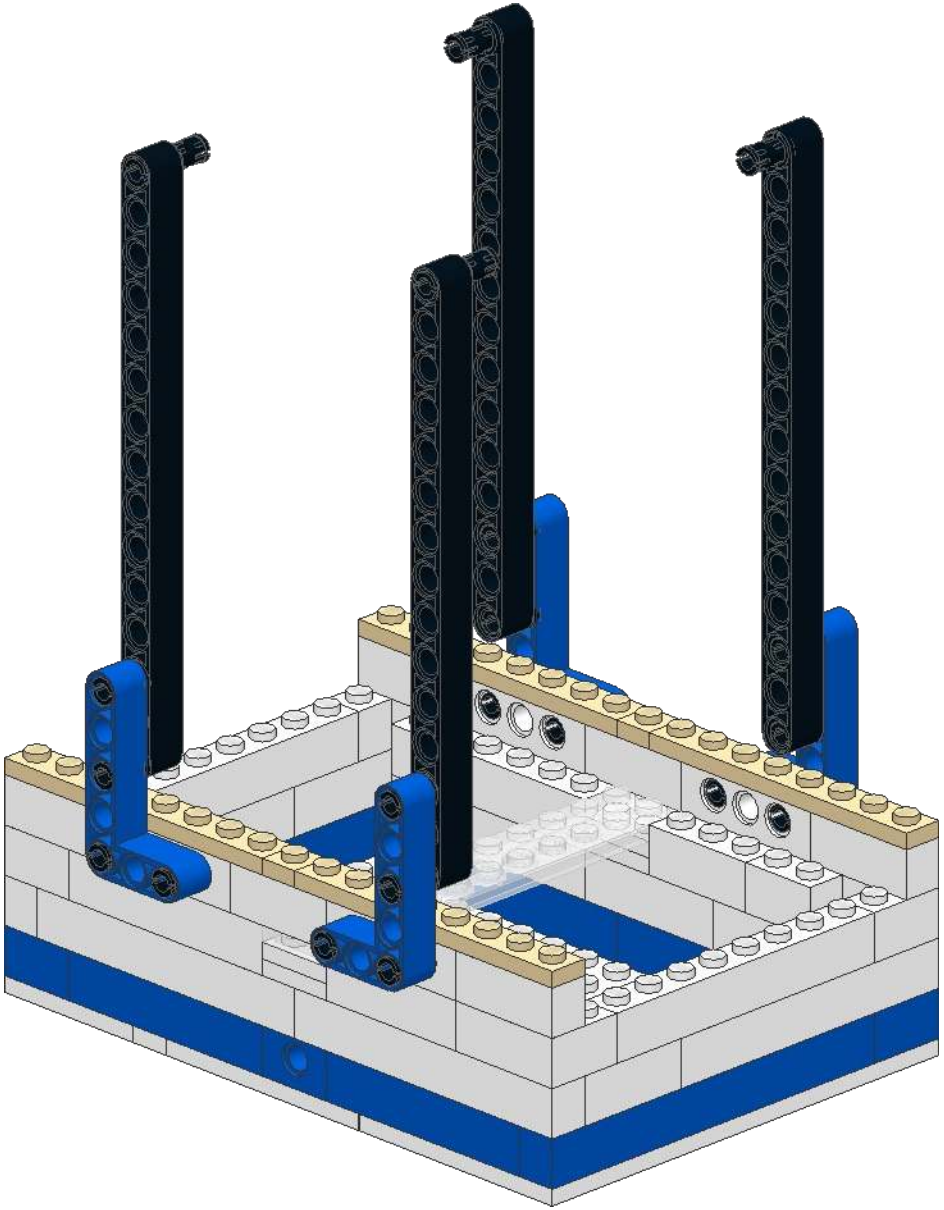

1

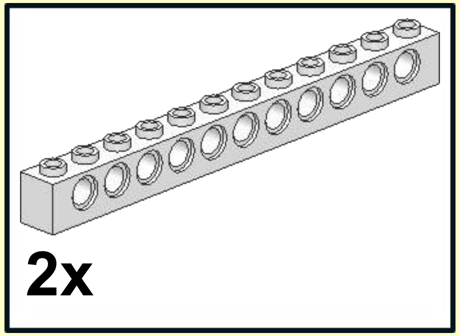

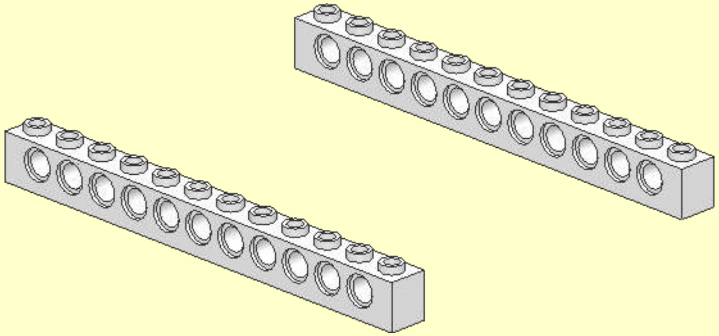

2

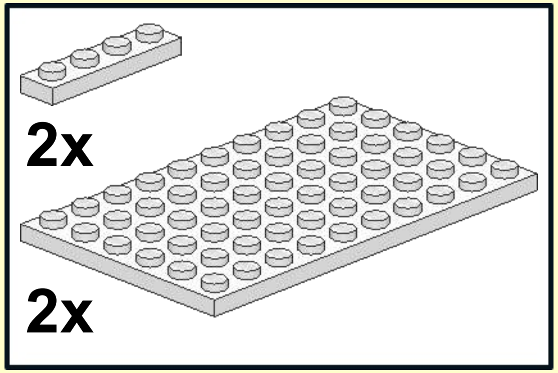

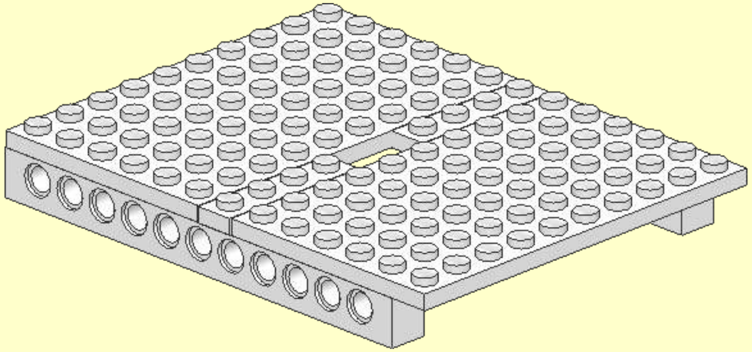

3

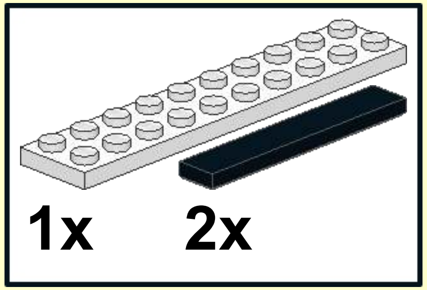

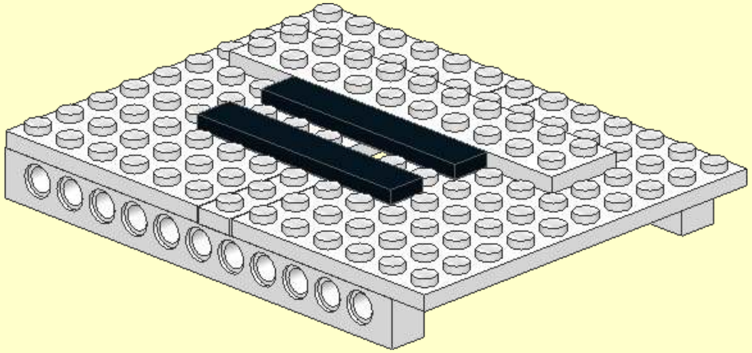

4

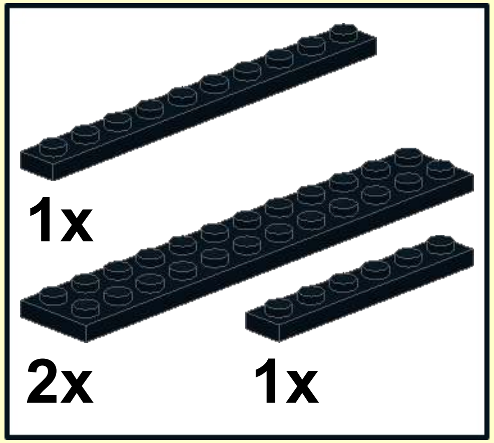

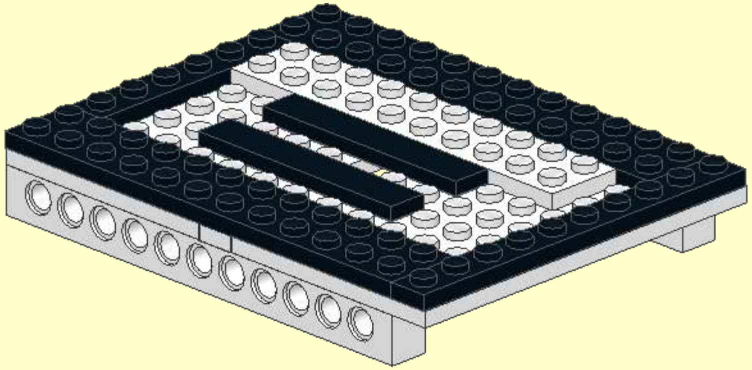

5

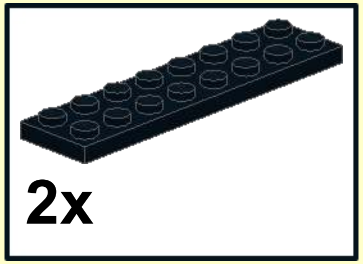

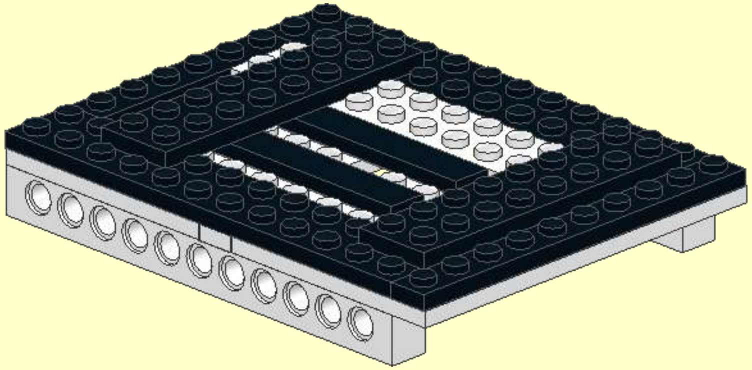

6

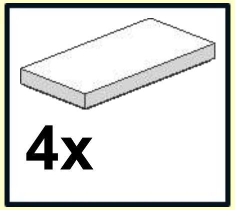

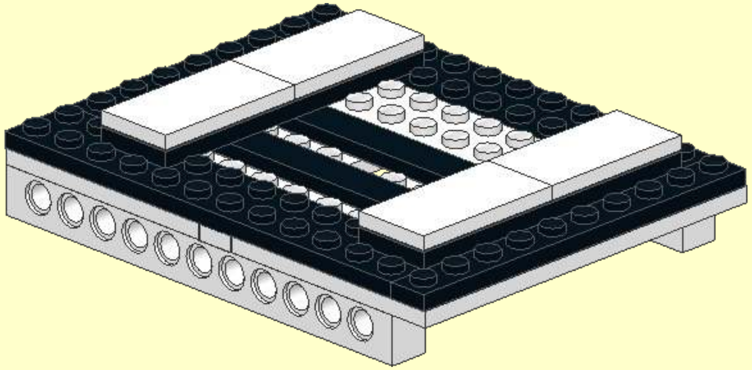

7

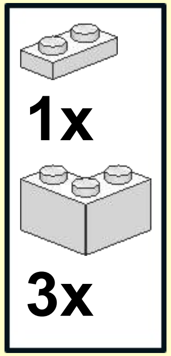

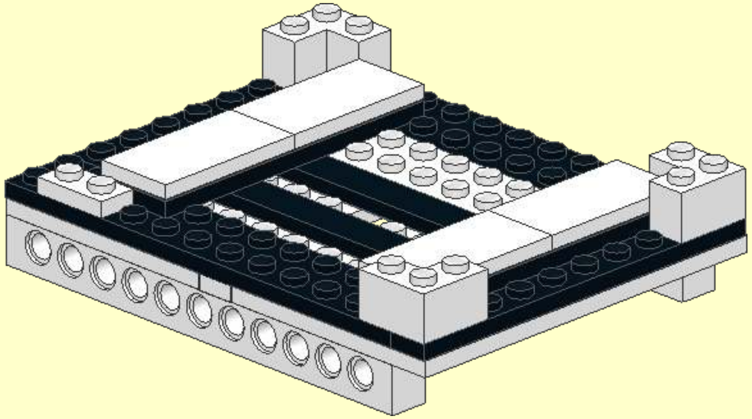

8

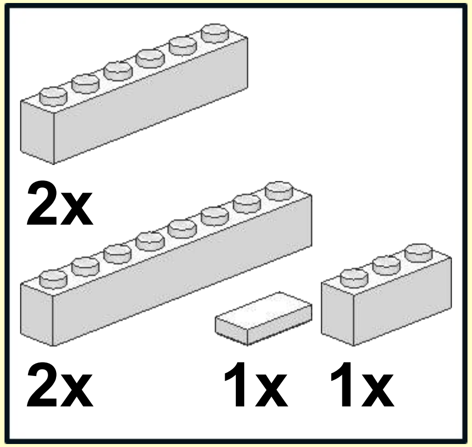

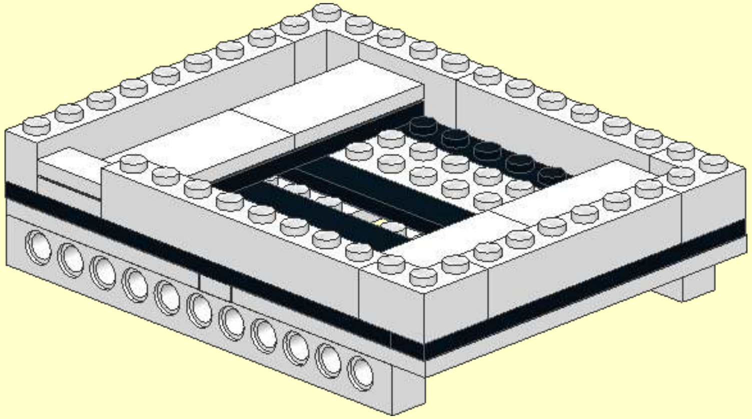

9

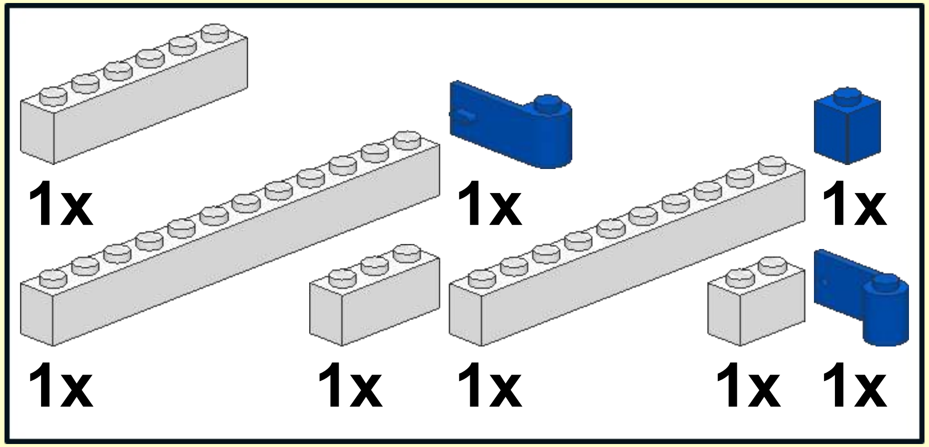

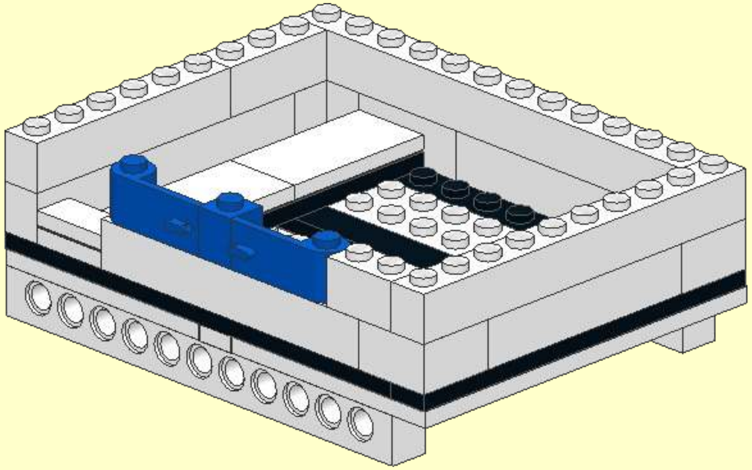

10

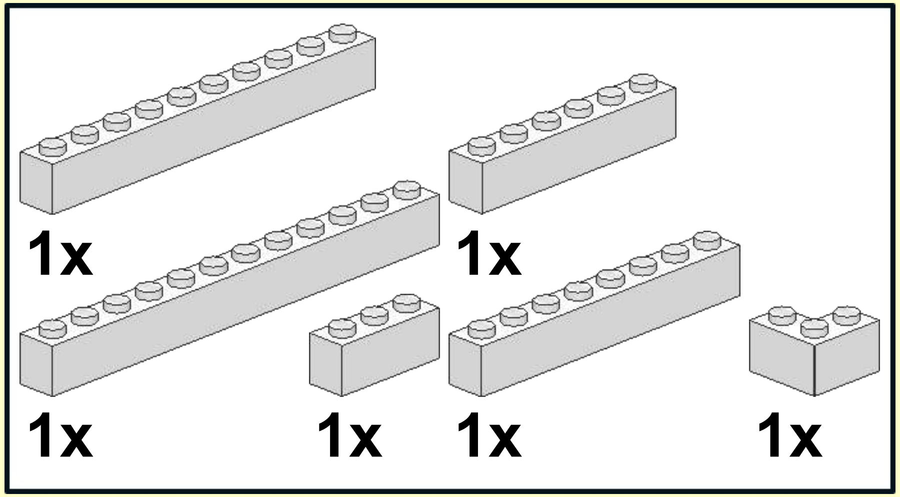

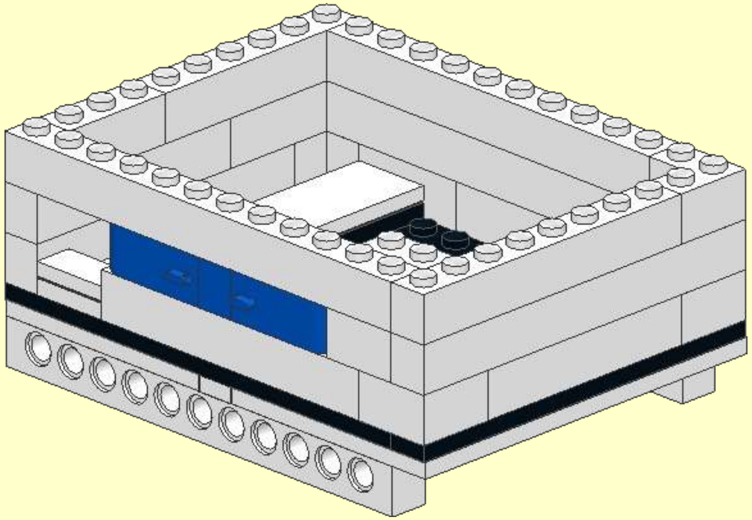

11

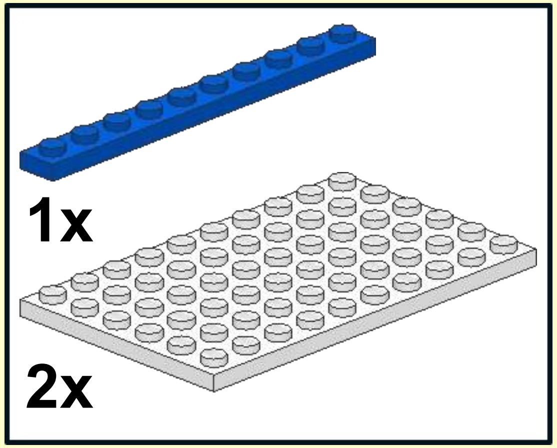

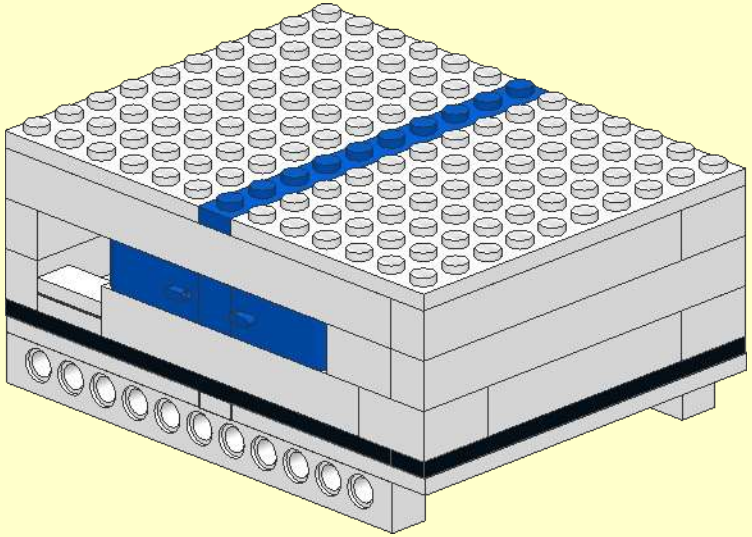

15

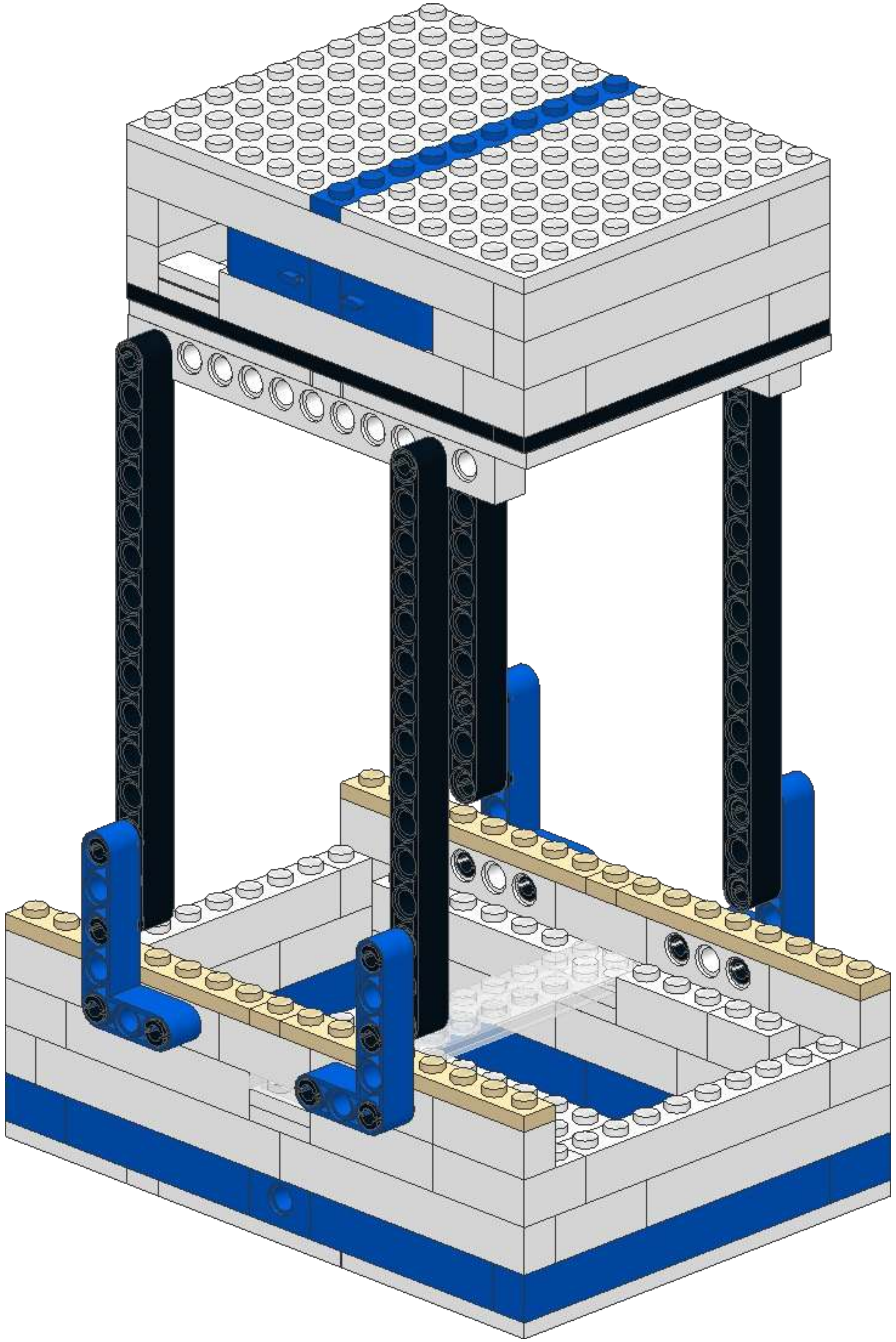

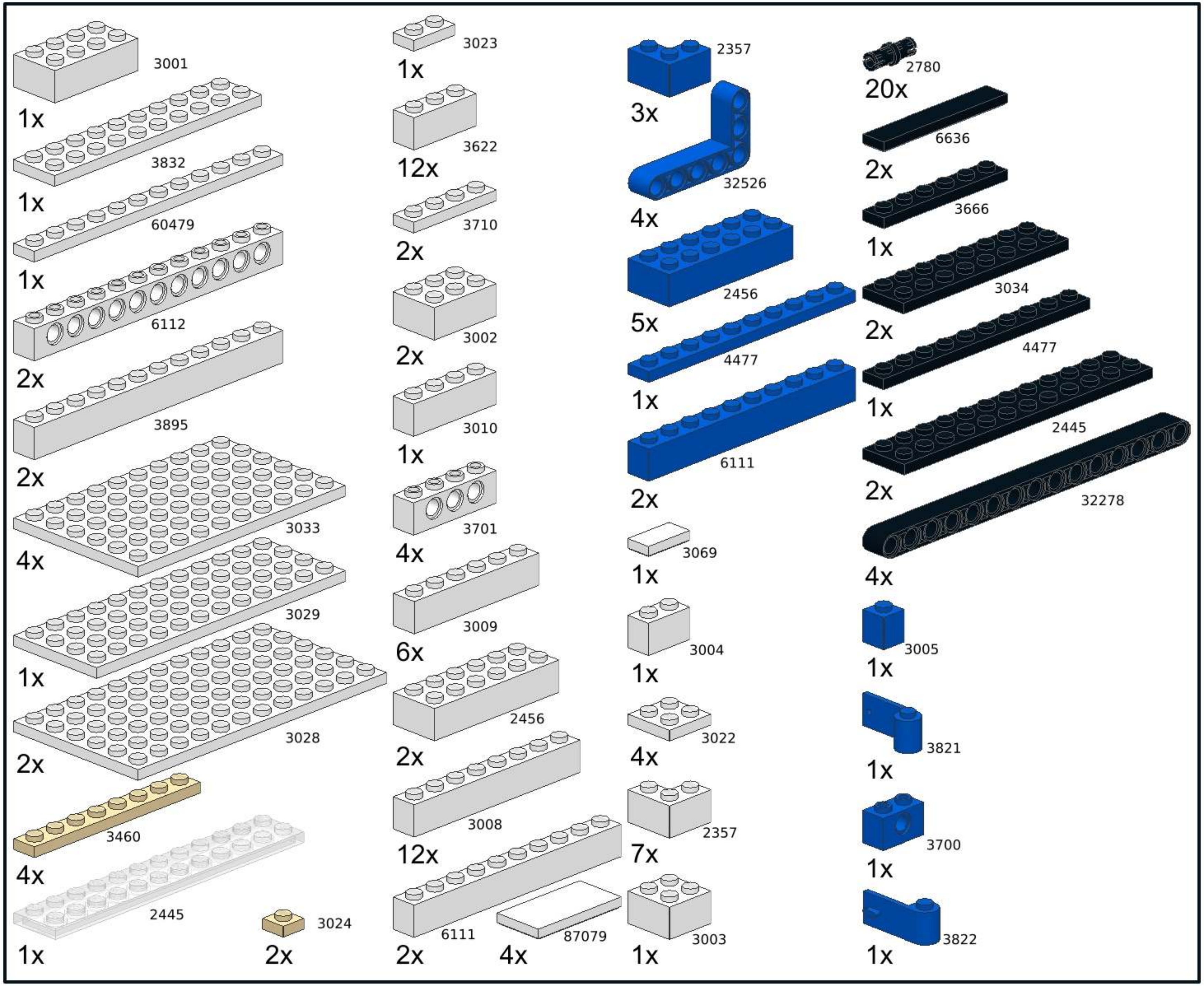
3001
1x
3832
1x
60479
1x
6112
2x
3895
2x
3033
4x
3029
1x
3028
2x
3460
4x
2445
1x
3024
2x
3023
1x
3622
12x
3710
2x
3002
2x
3010
1x
3701
4x
3009
6x
2456
2x
3008
12x
6111
2x
87079
4x
2357
3x
32526
4x
2456
5x
4477
1x
6111
2x
3069
1x
3004
1x
3022
4x
2357
7x
3003
1x
2780
20x
6636
2x
3666
1x
3034
2x
4477
1x
2445
2x
32278
4x
3005
1x
3821
1x
3700
1x
3822
1x

LEGO, the Minifigure and the bricks
shown in this brochure are trademarks
of the LEGO Group, which does not
sponsor, authorize or endorse this work.
The use of LEGO bricks in this document
is based on a fair-play spirit and
non commercial basis.

https://www.lego.com/en-us/legal/legal-notice/fair-play
