# Supplementary material for: Ethoscopes: An open platform for high-throughput ethomics
Source: PLoS Biol. 2017 Oct 19;15(10):e2003026. doi: 10.1371/journal.pbio.2003026 (PMC5648103; doi:10.1371/journal.pbio.2003026)
Supplement: S2 Text — (PDF) [file pbio.2003026.s003.pdf]

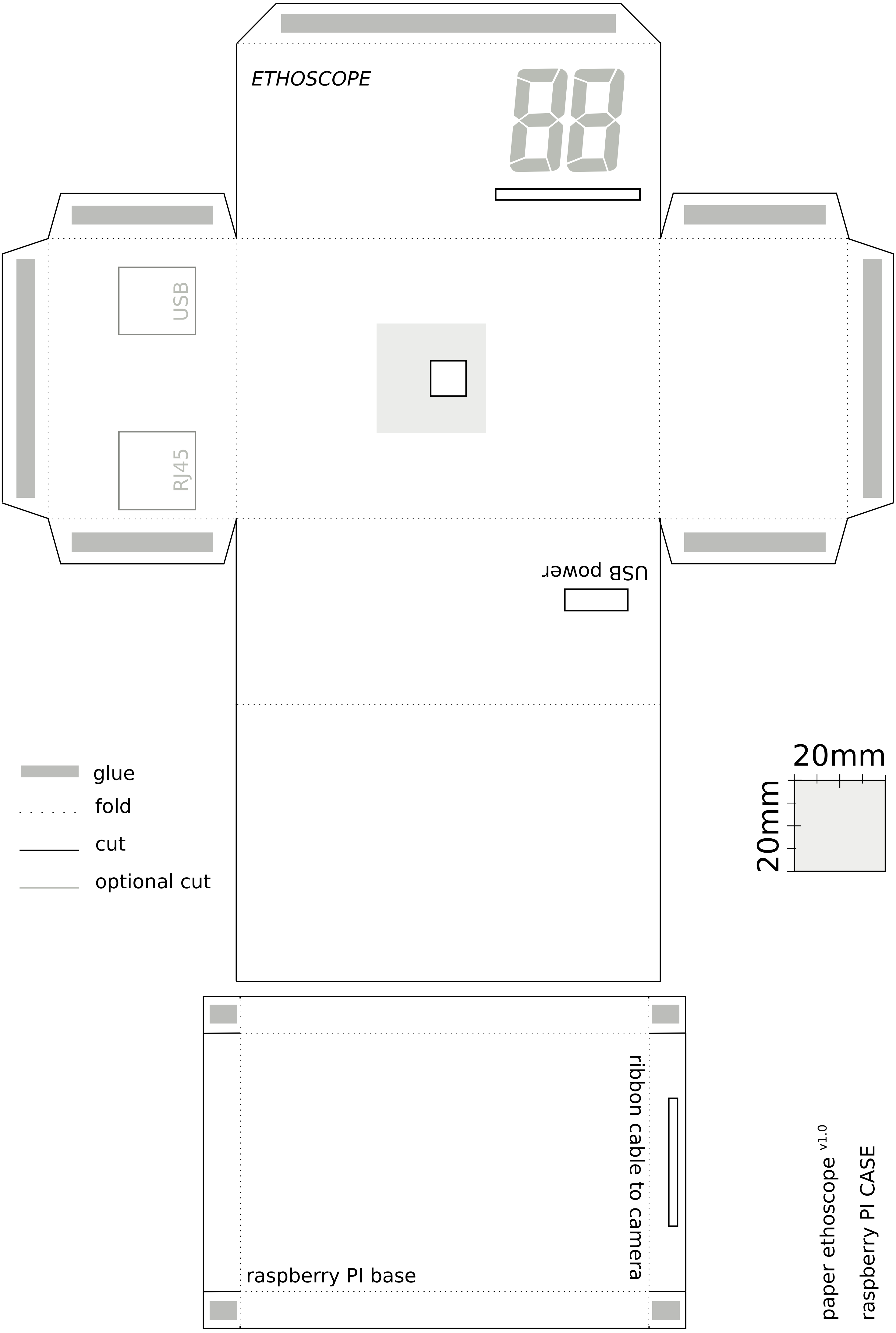
ETHOSCOPE
USB
RJ45
USB power
glue
fold
cut
optional cut
20mm
20mm
ribbon cable to camera
raspberry PI base
paper ethoscope v1.0
raspberry PI CASE

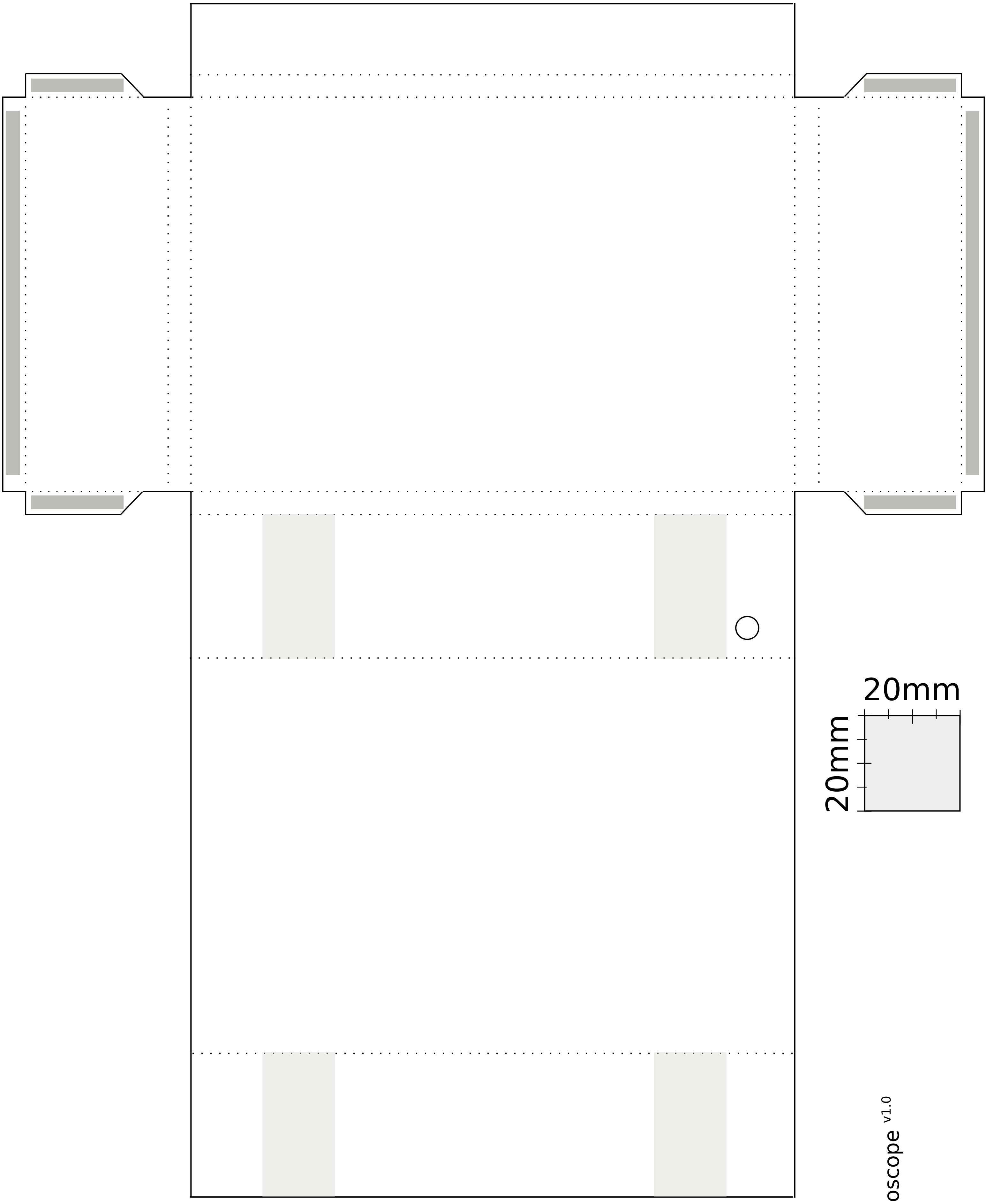

hole for IR power

guide for spacers

paper ethoscope v1.0

lightbox

1 | focus distance = 140mm | B | A

2 | focus distance = 140mm | D | C

3 | focus distance = 140mm | F | E

4 | focus distance = 140mm | H | G

Align with bottom of arena

Align with camera lens

3 | 4

1 | 2

D | K | B

C | K | A

E | M | G

F | M | H

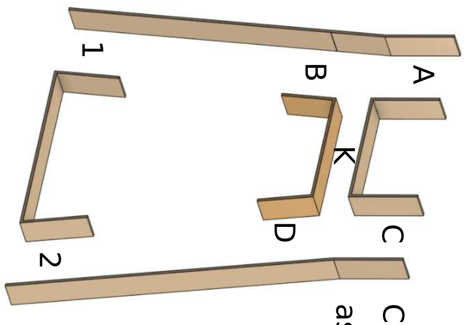

Cut on cardboard,
assemble using hotglue

20mm

20mm

paper ethoscope v1.0

spacers
