# Supplementary material for: Ethoscopes: An open platform for high-throughput ethomics
Source: PLoS Biol. 2017 Oct 19;15(10):e2003026. doi: 10.1371/journal.pbio.2003026 (PMC5648103; doi:10.1371/journal.pbio.2003026)
Supplement: S3 Text — (PDF) [file pbio.2003026.s004.pdf]

Table of Contents

Introduction 1.1
Building and Installation 1.2
    Ethoscope 1.2.1
    Arenas 1.2.2
    Router 1.2.3
    Node 1.2.4
    Ethoscope OS from Scratch 1.2.5
    Modules 1.2.6
        Optomotor 1.2.6.1
Using Ethoscopes 1.3
    Tracking Experiment 1.3.1
    Time in the Platform 1.3.2
    Error Messages 1.3.3
    Modules 1.3.4
        Scheduler 1.3.4.1
Administration and Maintenance 1.4
    System Update 1.4.1
    Restarting services 1.4.2
    Backing up Data 1.4.3
    Fixing Devices 1.4.4
Ethoscope API 1.5
Analysing the data with Rethomics 1.6

Ethoscopes are tools developed in the Gilestro lab to perform high throughput behavioural experiments. They are individual video tracking units based on the raspberry pi microcomputer.
Many ethoscopes are generally used concurrently within the same platform. The documentation herein is intended as a technical user manual for:

- People who wish to build and set up ethoscopes in their lab (from scratch)
- Experimenters who use them in the lab
- Maintainers who ensure the platform remains usable
- Developers who would like take part in the project (e.g. make new modules)

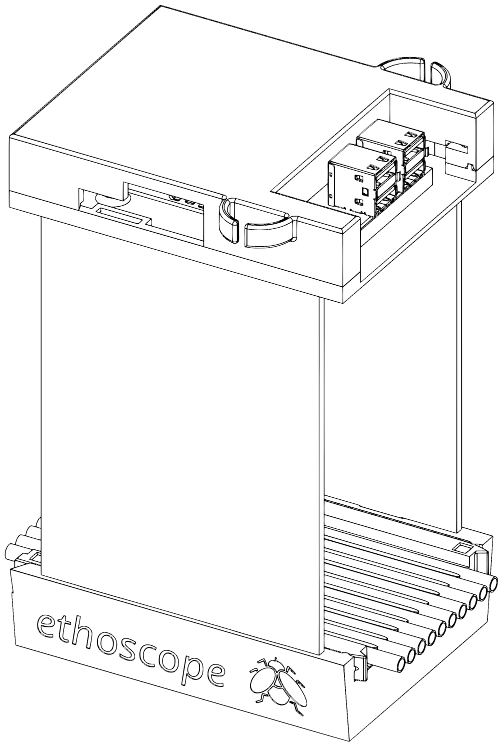

Links:

- Extra information about the ethoscope is available on **our website** http://gilestrolab.github.io/ethoscope
- The ethoscope platform is also published on biorxiv
- The **online version** of this manual can be accessed at https://qgeissmann.gitbooks.io/ethoscope-manual/
- A **printable pdf** version of this manual can be downloaded here

Building Ethoscopes

Ethoscopes are modular open source tools. As such, any one can build, modify or even sell them as long as you keep them open. For this reason, we spent considerable effort at the Gilestro lab to provide an up-to-date and exhaustive documentation for people who would like to build ethoscopes.

If you are setting up a platform from scratch, you will need three key components to get started.

1. At least, one ethoscope
2. A wireless network
3. A node

This diagram shows how these three components interact int the platform:

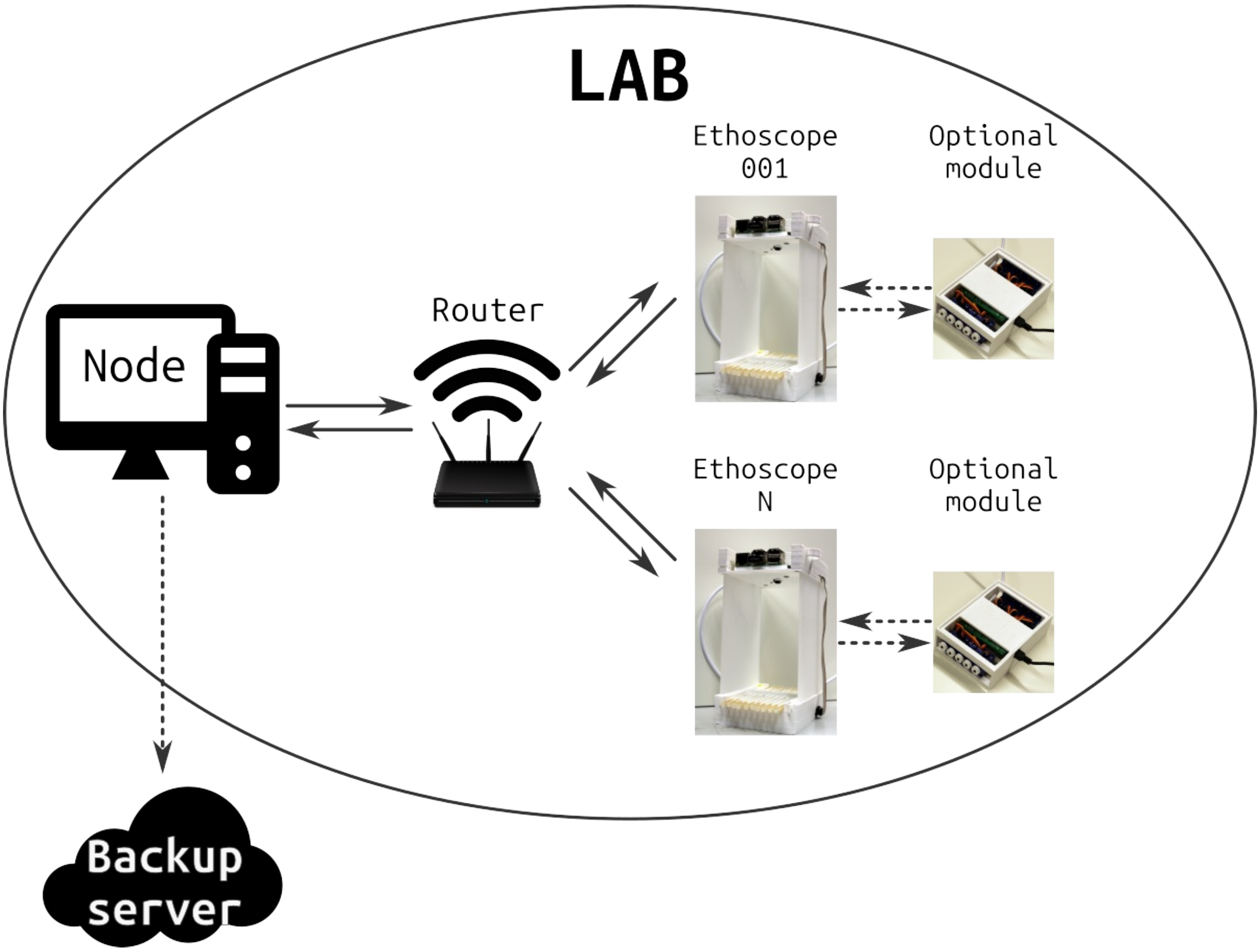

The strength of the ethoscopes is that you can use many of them in parallel. Therefore, the most time-consuming part will be to build these machines. Realistically, you will probably make a few to start with and test them one by one. Then, if you want to build many, it makes sense to have some sort of "production line".

All the files you need are contained in a vertioned zip bundle available at **https://zenodo.org/record/831153**

Building an Ethoscope

This section will detail the steps requiered to *build a functional Ethoscope* that can be used as part of the platform (along side a Node and a Router). A little bit of soldering is involved and, if you want to make your own parts, some 3d printing/laser cutting will be required too.

There are four main subsections:

- Introduction
- Getting the parts
- Burning the SD card
- Assembling it

Introduction

This part of the manual describes how to build the **core** of an Ethoscope. That is a device without an arena or extra modules. It is made of three main parts:

- The **head**, which contains the Raspberry Pi and its camera
- The **base**, which diffuses light through different types of arena (read about arenas here)
- The **side walls**, which simply connect the head to the base

An assembled core looks like this:

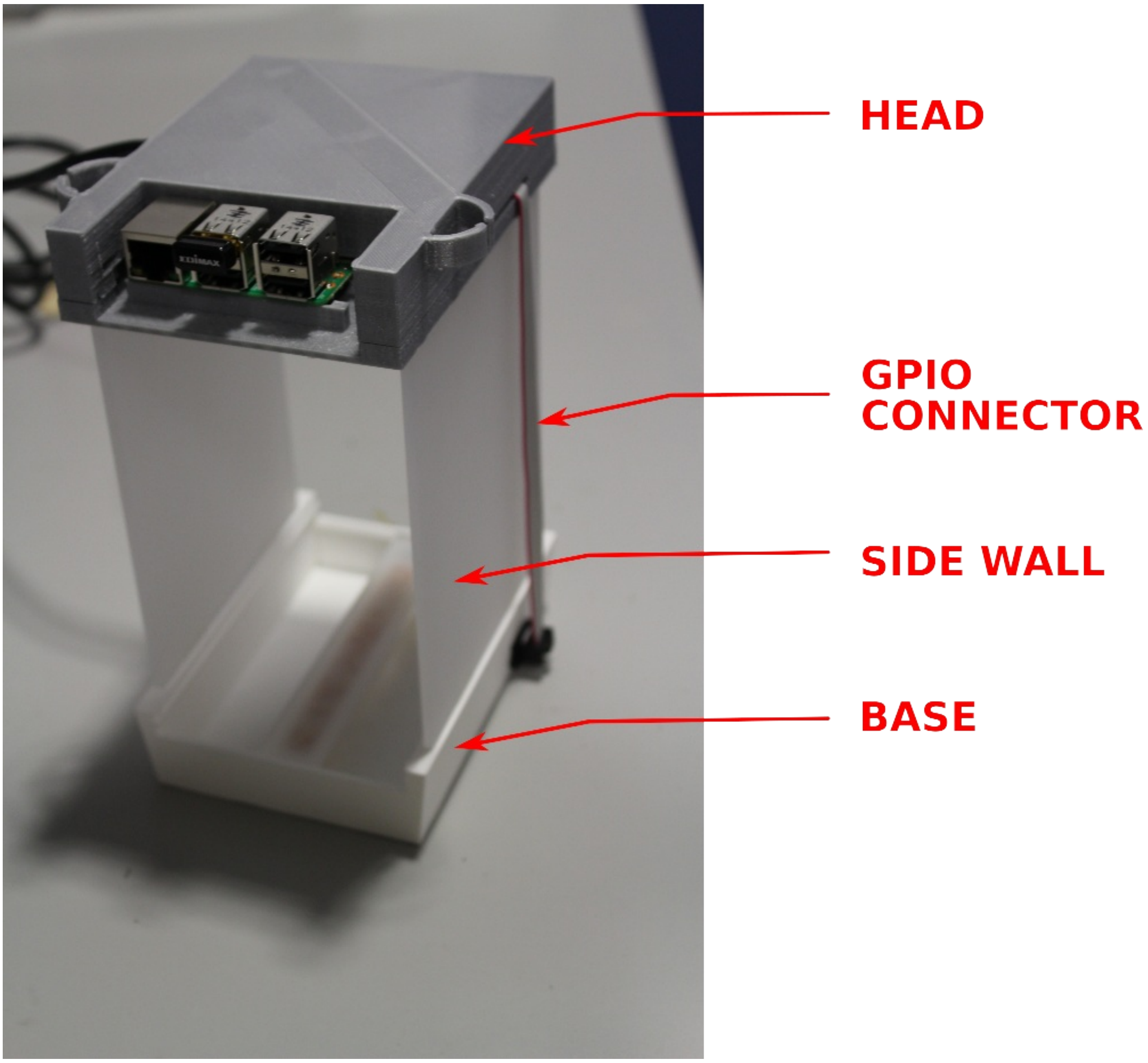

Getting the parts

Ensure you have dowloaded all the files you need from our zip bundle at https://zenodo.org/record/831153.

Before building ethoscopes, you need to acquire various parts, that are described in the following Build Of Material (BOM) table.

Piece	Part	Quantity	Supplier	Reference	Price (GBP)
Lower Pi case	Head	1	3d printed	onshape	1
Upper Pi case	Head	1	3d printed	onshape	1
Raspberry Pi v3	Head	1	CPC	onshape	20
PiNoir camera V2	Head	1	RS	RS 790-2811	15
32Gb SD card	Head	1	amazon	32G Samsung EVO	8
M3 12mm bolt	Head	6	RS	RS 232-8372	0
M3 nuts	Head	4	RS	RS 560-293	0
M2 nuts (nylon)	Head	2	RS	RS 528-126	0
M2 6mm bolts	Head	2	RS	RS 482-7685	0
Light box	Base	1	3d printed	onshape	0
Light support	Base	1	3d printed	onshape	0
Male 2x6 IDC socket	Base	1	RS	RS 832-3496	1
12V IR(850nm) LED strip	Base	3LED	Lightworld	Lightworld	1
5cm cable (2 wires)	Base	2	RS		0
Side wall V>=9	Side Wall	2	Laser cut	onshape	0
Ribbon cable (6 way 18cm)	GPIO connector	1	1	RS	1
Female 2x6 IDC socket	GPIO connector	1	RS	RS 832-3648	1
Female 2x40 IDC socket	GPIO connector	1	RS	RS 625-7303	1
USB A to micro cable	Power supply	1	RS	RS 888-8811	3
Anker 40w PSU	Power supply	1	Amazon		12

As you start building, you should have something like that:

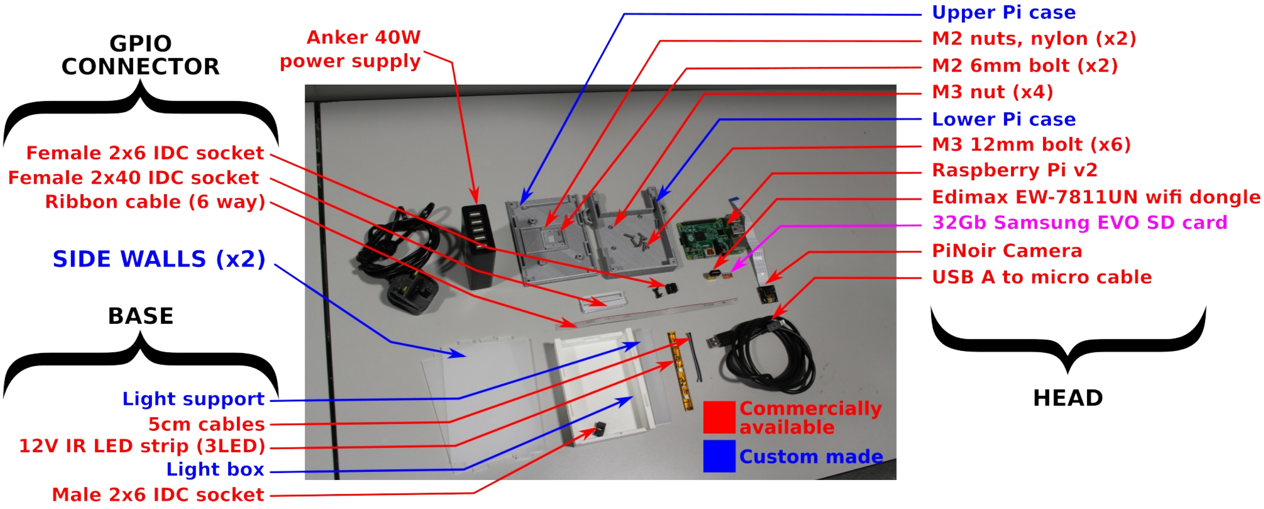

At this stage, it is important to **make a difference between the parts that you can purchase (red) and those that you have to build yourself (blue)**.

NOTE: Ethoscopes can be built indifferently with raspberry pis version 2 or 3. Unless you have a good reason, **use version 3**. If your work the version 2, you will need to also use a wifi dongle (version 3 has on board wifi).

If you need to 3d print and laser cut your parts, the simplest way is to directly get the files (STL and pdf) in from ZIP archive you already downloaded (see link above). In addition, those parts are available through a link (see reference column in BOM) to our onshape repository.

Since we run custom software on the Rasberry Pi, you will also have to build your SD card (as it runs contains the operating system).

Burning the SD card

When burning a card, there are two steps:

1. Formatting and copying the files
2. Naming the card

This subsection involves some familiarity with Linux/Unix command line, and **you probably want to work from a Linux or mac computer**. If you feel like building the SD card yourself (which involves much more work and background), have a look here instead.

Formating

The SD card in the pi is exactly like the hard drive of a regular computer.

Essentially, we want to install a custom ArchLinux so we will follow these instructions, but replace the latest stable ArchLinux by our custom OS snapshot which is also available **inside the zip archive** (it has a name like `ethoscope_os_YYYYMMDD.tar.gz`).

So, to summarise, **as root** (not sudo) do:

Get the OS:

```
cd /tmp/
wget https://zenodo.org/record/831154/files/ethoscope_core_files.zip
unzip ethoscope_core_files.zip -d ethoscope_core_files && rm ethoscope_core_files.zip
```

We make the partition table:

```
SD_CARD=/dev/sdi
echo "o
p
n
p
1

+100M
t
c
n
p
2


w"| fdisk $SD_CARD
```

Format partitions:

```
mkfs.vfat ${SD_CARD}1
mkdir boot
mount ${SD_CARD}1 boot
mkfs.ext4 ${SD_CARD}2
mkdir root
mount ${SD_CARD}2 root
```

Transfer OS to partitions (check that the date, in the file name, matches your file):

```
bsdtar -xpf ethoscope_core_files/20160721_ethoscope_root.tar.gz -C root
sync
mv root/boot/* boot
umount boot root
```

Naming

So that is it for building a generic card. However, when you build several machines, you will want to identify each of them in a unique and unambiguous manner.

You want to edit three files contain information that is unique to each device.

Then you want to change the ID of the machine on the card, so rewrite:

- `/etc/machine-id` . This is a hexadecimal unique name for the machine. You can put some random string *prefixed with a number*. For instance `001ae2f5cee1` ...
- `/etc/machine-name` . This is the human-friendly name of the machine. For instance ETHOSCOPE_001.
- `/etc/hostname` . This is the name of the machine on the network. For example, you could put e001.

TIP: Use a permanent marker to *physically* write its name on the card, so you can match the card to its device.

ANOTHER TIP: if you need to make many cards, I strongly advise to buy multiple SD card readers so you can write concurrently. Also, It is less error-prone to first burn all the cards (several at a time), and afterwards name them one by one.

Assembling it

First, you will need several standard tools and consumables such as:

- Allen keys
- soldering iron
- super glue
- heat shrink
- solder wire (lead-free).

GPIO connector

In order to power the infrared (IR) light, the General Purpose Input Output (GPIO) of the Raspberry Pi must be connected to the base by a ribbon cable. To do so, we can build a simple connector that looks like that:

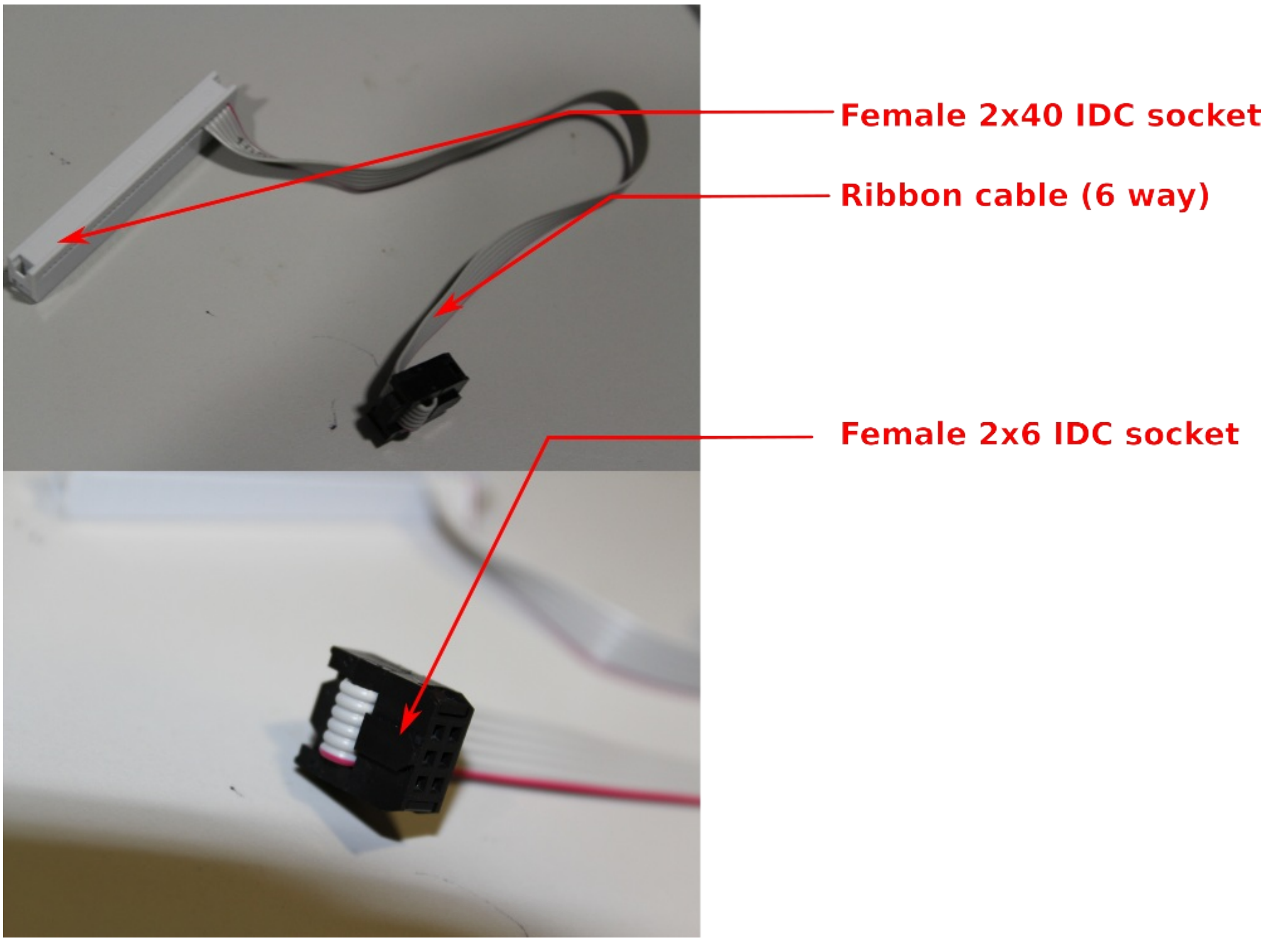

Here, we have used a **18cm long** 6 ways ribbon cable.

Base

The base acts as an IR light box. The idea is to reflect IR light upward so it can go through the arena. We will be using the raspberry pi pins 4 (5v) and 6 (GND) to supply power to the IR LED strip.

In order to put the light together, a little bit of basic soldering is required:

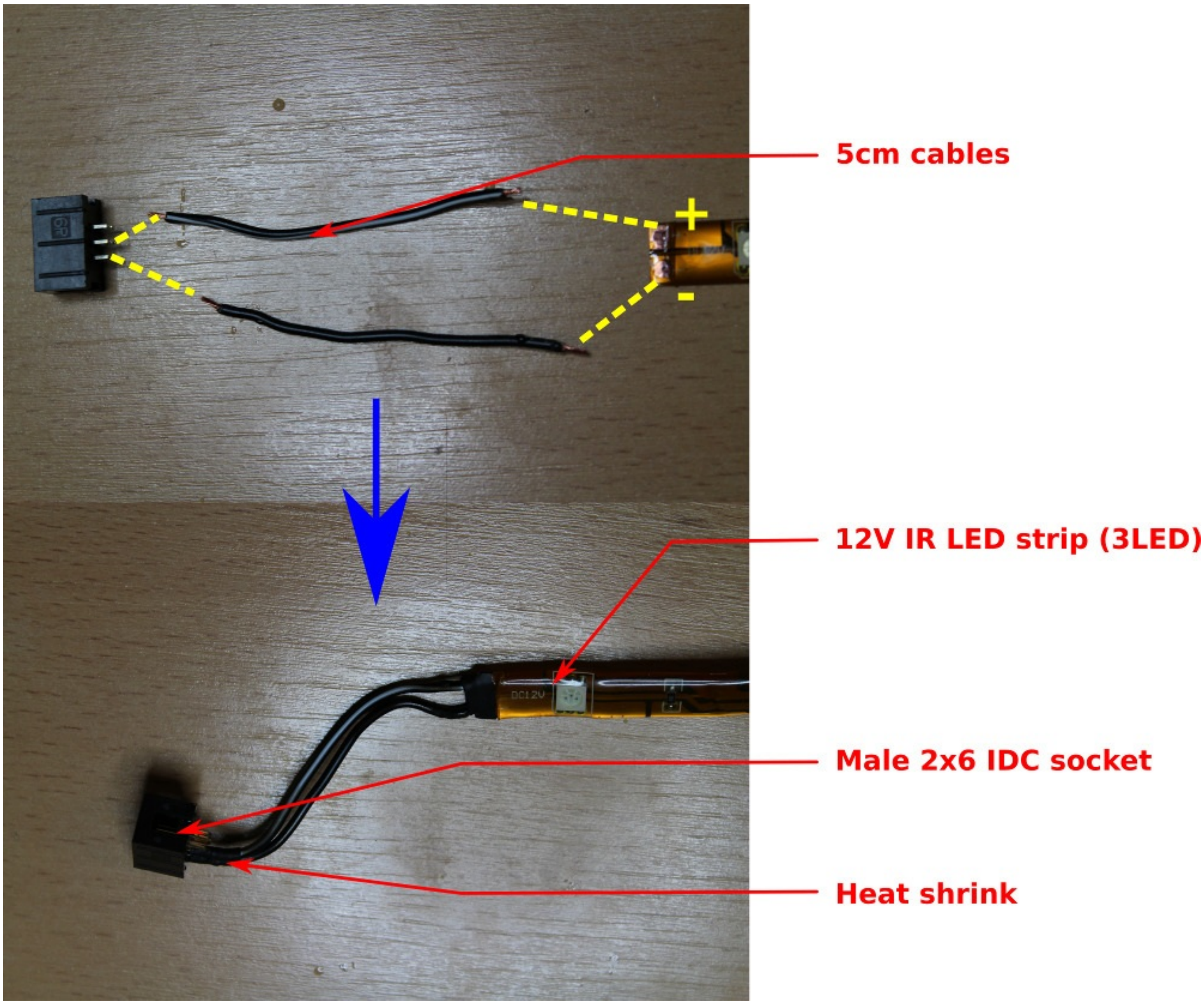

In the figure above, the yellow dashed lined indicate where we need to solder (Pins 4 and 6 of the 2x3 connector).

You can also notice that **heat shrink** have been put around the wires so that we ensure proper insulation.

After is has been soldered, the led strip must be passed **from the outside** to the inside of the light box by the connector hole. Then, we can stick the light to its support, and glue the support to the side of the base:

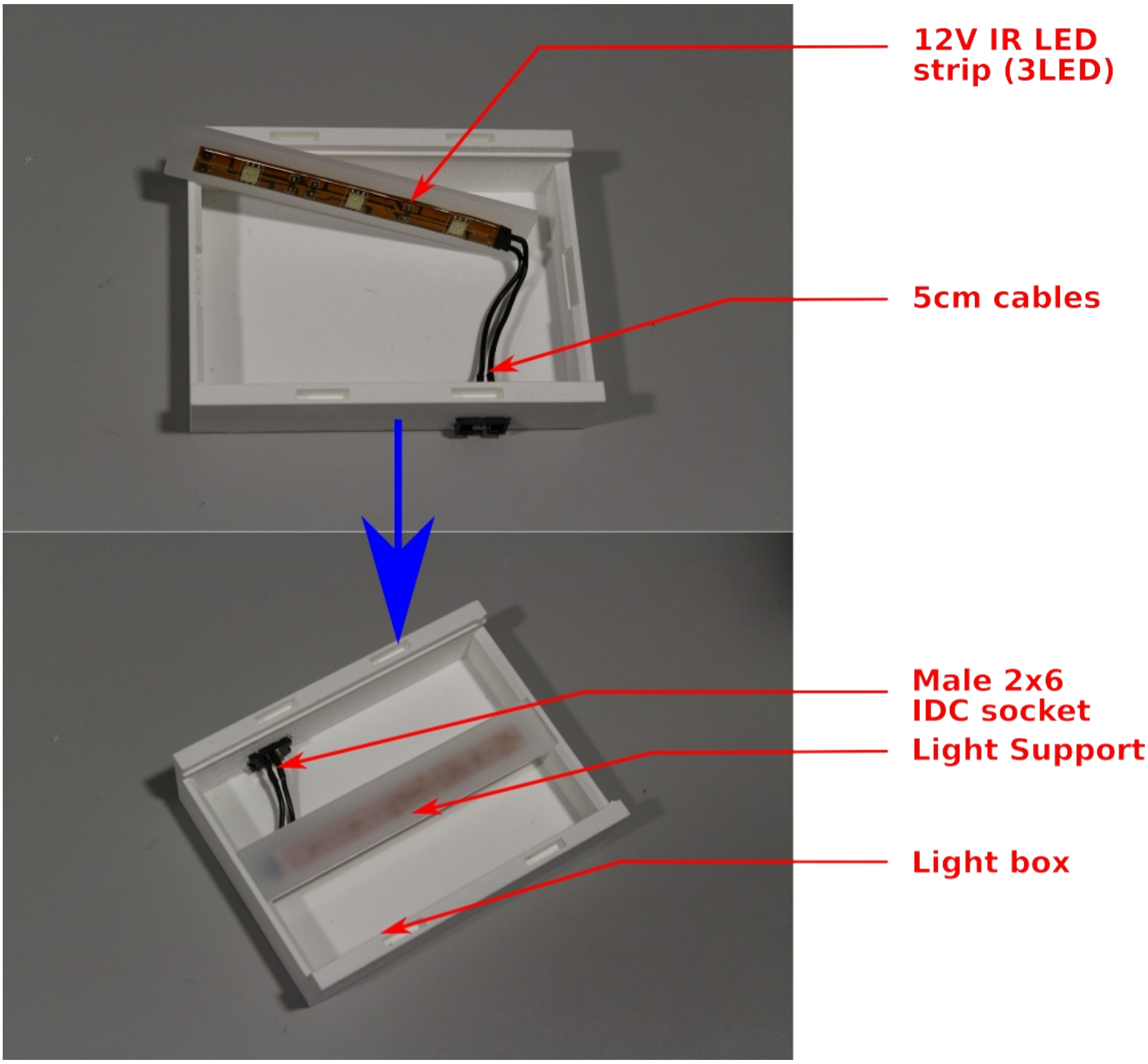

Head

The head is the single most important part of the device. It is made of a Raspberry Pi, and peripherals, enclosed by a pair (lower and upper) of 3d printed parts.

The steps to build the head are the following:

1. Generate a **SD card** (it contains the operating system and all the software used by the ethoscope).
2. Plug the SD card in the Pi.
3. Place the **camera** on the lower part, and screw from the outside using M2 bolts along with the nylon nuts (sub-picture A, below).
4. Position the **Raspberry Pi** in the lower part, above the camera, and screw it from the outside, with two M3 bolts (sub-picture B, below).
5. Connect the camera, the **GPIO connector** to the Pi.
6. In the upper part, there are four holes in the corners. They allow us to close the box with

screws. You want to **insert a nut in each slit** under the hole,

7. Now, we can **close the box** (sub-picture C, below) and screw the upper and lower parts together, using four M3 screws.
8. The last thing we want to do is plugging the micro-USB power supply in the Pi.

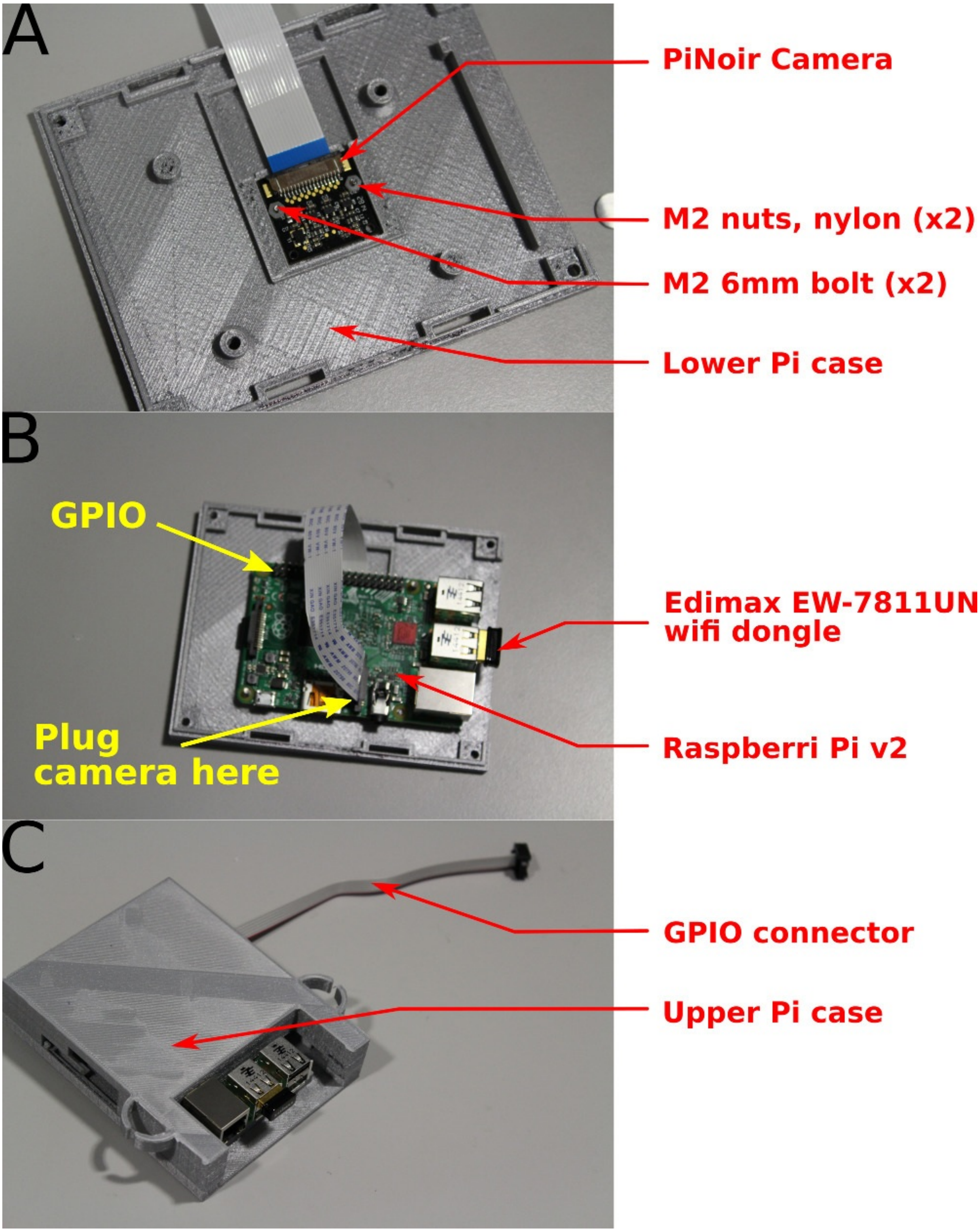

Last bits: Before you can start working, you will have to **change the focus of the camera**. I am afraid this has to be done manually.

For old camera versions (1.3) you can find an online video explanation, be careful since the lens is glued.

If you are using the second version of the camera (2.x) it is easier since the lenses are not glued. You should have received a tool with your camera. If not, you can order it online.

In case you use the pi v2, you will need a wireless dongle. It may blink with blue light to indicate that it transmits data. According to your experimental setup, you may want to blind the LED (e.g. with black tape or something like nail varnish) in order to prevent interference with the behaviour of your experimental animals.

Arenas

There are several types of behavioural arenas that can be used with the ethoscope. Most users will work with the regular arena, which is provided in the ZIP bundle (https://zenodo.org/record/831153).

It looks like this:

Very important: Arenas must have "targets". targets are 4mm-diameter black disks that should are stuck on the arena. **Targets are essential** for the software to define regions of interest. You will not be able to start tracking experiments without targets! A regular printer, a pair of scissors and some paper glue will do the job. The ZIP bundle also contains targets on an A4 page that you can print. You need to ensure the target are circular (cut them accurately), contracted and visible.

All our arenas are available on onshape.

Setting up Your Router

The idea is to connect several ethoscope devices to a unique node via a single wireless network. To do so, we can work with a router similar to the one you may have at home. If you have ever set-up your home router, this is very similar and does not require too much knowledge. According to your router, you may reach a limit in the number of machines you can connect to the same network, so you may want to use a more professional solution. In this scenario, we want to connect all ethoscopes through wifi, but the **node through an ethernet cable**. This is because the node will download periodically all the data from all the devices, so we avoid overusing the wireless network.

Configuration

Here, we give a default configuration that matches the rest of the rest of the instructions. If you know what you are doing, you can, of course, change these. This could be helpful if, say, you wanted to have several networks in the same room.

Wireless configuration

- The **name of the network** is `ETHOSCOPE_WIFI`
- The **password** is `ETHOSCOPE_1234`

DHCP

- **start ip** is `192.169.123.6`
- **end ip** is `192.169.123.250`
- **default gateway** is `192.169.123.254`

A **very important thing** is to reserve the ip `192.169.123.1` for the node. You will need the MAC ip its **wired interface** for that. **DO NOT PUT THE MAC IP OF THE WIRELESS (or any other) INTERFACE**

In order to get it, you can use `ip link` on the node. You will get something like:

```
$ ip link
1: lo: <LOOPBACK,UP,LOWER_UP> mtu 65536 qdisc noqueue state UNKNOWN mode DEFAULT group
 default
link/loopback 00:00:00:00:00:00 brd 00:00:00:00:00:00
2: enp3s0: <BROADCAST,MULTICAST,UP,LOWER_UP> mtu 1500 qdisc fq_codel state UP mode DEF
AULT group default qlen 1000
link/ether 10:bf:48:ba:d3:84 brd ff:ff:ff:ff:ff:ff
3: wlp5s1: <BROADCAST,MULTICAST,UP,LOWER_UP> mtu 1500 qdisc mq state UP mode DORMANT g
roup default qlen 1000
link/ether 00:26:5a:e6:47:f1 brd ff:ff:ff:ff:ff:ff
```

In this exanple, my wired interface is `enp3s0` (all wifi interfaces start with `wl`), then my MAC address is `10:bf:48:ba:d3:84`

Professional solutions

A classic home router will fail to connect more than around 30 devices. A simple solution that we have been using in the lab is to purchase and configure a professional access point like Ubiquiti's Unifi. This one is relatively inexpensive and simple to set up. In the lab, we use it to connect more than 75 devices at a time.

Setting up Your Node

This section describes the few modifications needed to set-up a node.
It requires, at least, some familiarity with Linux system.

The node is a regular computer, that is configured to:

- Run a web server in order to detect, start, stop,... devices (http://node:80)
- Run a web server to update devices (http://node:8888)
- Run a backup tool, that fetches data from all detected devices every 5min
- Run an NTP server (so it used as the central clock of the platform)
- Run a video backup tool, that fetches video chunks from recording devices (we can record instead of tracking)
- Ideally, it also backs-up periodically the data to a cloud service (so we avoid bad surprises)

The node simply orchestrates the platform, **it does not analyse any data**.
Indeed, tracking is performed, in real time, by each device.
Hence, if some devices are running and the node (or the network) shuts down, *tracking will not be interrupted*, and the data will be backed up on the node as soon as it is running again.

Here is a checklist of the task that you need to perfomr in order to get a running Node, detailed instructions are below.

1. Get a computer that has hardware compatibility with Linux, basically any PC will do. (check minimum recomended settings)
2. Install Linux, we recommend to use Antergos (or any othe Arch Linux based distribution)
3. Install packages that does not come preinstalled in the normal distribution.
4. Create a clone of the offical ethoscope repository with the property Bare. Reasons to do that explained below.
5. Create a clone from the repositiory created in step 4.
6. Install the Node software from repository created in step 5.
7. Set up the network connections, local ethernet and Internet ethernet.
8. Startup all the daemons that are neede to run the Node servers indefinitely.
9. Set the time to UTC and reboot the system.

Hardware

The hardware specification for the node can, therefore, be pretty standard, I would go with:

- CPU, anything made after 2014 should work (e.g. intel i3)
- RAM 4GB or more
- A decent hard-drive as you will backup GB of data on it
- A wireless adapter (or an extra ethernet port), to go on the internet (as the primary ethernet port will connect to our custom network)
- Also, check that the hardware can run Linux.

Installing a node

The OS

Once we have put our hands on some hardware, we need to install an operating system.
In our lab, we prefer Arch Linux based distributions such as Antergos. All the steps that follow only work in a Arch Linux based machine.
Antergos provides installation instructions.
Once you boot from the USB stick, the installation wizard will take you through.
I would go for a standard installation, with gnome as desktop manager.
As a user name write **node**.

Installing extra packages

The first thing we will do after installation is getting additional packages we need.

```
# update all packages
pacman -Syu --noconfirm

# tools for developers
pacman -S base-devel git gcc-fortran rsync wget fping --noconfirm --needed

# utilities
pacman -S ntp bash-completion openssh --noconfirm --needed

#so we can set up a dns
pacman -S dnsmasq --noconfirm --needed

# pre-installing dependencies will save compiling time on python packages
pacman -S python2-pip python2-numpy python2-bottle python2-pyserial mysql-python pytho
n2-netifaces python2-cherrypy python2-futures --noconfirm --needed

# mariadb
pacman -S mariadb --noconfirm --needed

# setup Wifi dongle
pacman -S wpa_supplicant --noconfirm --needed
pacman -S libev --noconfirm --needed
```

Creating a git bare repo

Here we setup a local git bar repo that should mirror https://github.com/gilestrolab/ethoscope. This repository will not contain data, but only tracks the changes, that is why it has the special Bare property. We do that in order to be able to separate the devices from the real world internet. Devices will see this bare repo as the master repository and update from it. The Node will be in charge of actually syncronize this bare repository with the real repositoty on Github. Indeed, the Node software is updated using this Bare repositoty and not a direct connection with github.

```
# a few variables
UPSTREAM_GIT_REPO=https://github.com/gilestrolab/ethoscope.git
LOCAL_BARE_PATH=/srv/git/ethoscope.git

mkdir -p /srv/git
git clone --bare $UPSTREAM_GIT_REPO $LOCAL_BARE_PATH
```

The node software

After that, we can clone ethoscope software and install the node part. With this step, we create a normal repository (with data) in the Node, and we can use the cloned repo to actually install all the custom made software.

```
# variables
TARGET_UPDATER_DIR=/opt/ethoscope_updater
TARGET_GIT_INSTALL=/opt/ethoscope-git

#Create a local working copy from the bare repo on node
echo 'Installing ethoscope package'
git clone $LOCAL_BARE_PATH $TARGET_GIT_INSTALL

cd $TARGET_GIT_INSTALL

# IMPORTANT this is if you want to work on the "dev" branch otherwise, you are using "
master"
git checkout -b dev

cd $TARGET_GIT_INSTALL/node_src
# we install with pip
pip2 install -e .
```

Network

The idea is to set up the network so that we can use, at the same time, a connection to the internet and the intranet (in house router).

We will use a DNS server to resolve the IP of the Node. To achive that, first, we create a dnsmasq configuration file. This will create a local DNS server in the node that will resolve names in the local network.

```
# see how to setup router
NODE_IP=192.169.123.1

#configuring dns server:
echo "interface=$WL_INTERFACE" >/etc/dnsmasq.conf
echo "dhcp-option = 6,$NODE_IP" >> /etc/dnsmasq.conf
echo "no-hosts" >> /etc/dnsmasq.conf
echo "addn-hosts=/etc/host.dnsmasq" >> /etc/dnsmasq.conf
# so that http://node can be our homepage
echo "$NODE_IP node" >> /etc/hosts.dnsmasq
```

In order to connect to the wireless interface, the simplest is to go in the network configuration interface.
Just click on the network icon on the top-right of the screen, select `ETHOSCOPE_WIFI` . The default password is `ETHOSCOPE_1234` (see network instruction).

System daemons

We need to enable some utilities though `systemd` :

```
# Enable networktime protocol
systemctl start ntpd.service
systemctl enable ntpd.service

# Setting up ssh server
systemctl enable sshd.service
systemctl start sshd.service

# to host the bare git repo
systemctl start git-daemon.socket
systemctl enable git-daemon.socket
```

Our own daemons

Now, we want to enable our own daemon/tools

```
# this is where our custom services are
cd $TARGET_GIT_INSTALL/scripts

cp ./ethoscope_node.service /etc/systemd/system/ethoscope_node.service
cp ./ethoscope_backup.service /etc/systemd/system/ethoscope_backup.service
cp ./ethoscope_video_backup.service /etc/systemd/system/ethoscope_video_backup.service

systemctl daemon-reload

systemctl enable ethoscope_node.service
systemctl enable ethoscope_backup.service
systemctl enable ethoscope_video_backup.service
```

In addition to the node services, we want to run the update daemon.
It is important that the update server is copied out of the git repository. This way, the update does *not* update itself.

```
UPDATER_LOCATION_IN_GIT=scripts/ethoscope_updater
cp $TARGET_GIT_INSTALL/$UPDATER_LOCATION_IN_GIT $TARGET_UPDATER_DIR -r
cd $TARGET_UPDATER_DIR
cp ethoscope_update_node.service /etc/systemd/system/ethoscope_update_node.service

systemctl daemon-reload
systemctl enable ethoscope_update_node.service
```

Time

It makes everything easier if you force time to be GMT regardless of your time zone and season:

```
timedatectl set-timezone GMT
```

The node will act as an NTP server for ethoscopes.
That is machines will use local network to sync time from the node.
However, by default, **the server (node) will not serve time if it fails to get it from internet**.
In order to allow the node to server time regardless, you can add these two lines to `/etc/ntp.conf` :

```
server 127.127.1.1
fudge 127.127.1.1 stratum 12
```

What is next

In order to check things:

- Reboot the computer
- Open firefox
- Test the local server at http://0.0.0.0
- Test the local server at http://192.169.123.1 (will fail until your network is configured)
- Test the update server http://192.169.123.1:8888
- Test the dns server (go at http://node)

Getting the data somewhere

The node will save that data in two places:

- `/ethoscope_results` , which is the root of all the tracking data ever recorded.
- `/ethoscope_videos` , the root of the video file chunks when recording video.

You probably want to synchronise these directories to a network drive, for instance using Rsync. If you use a script to sync `/ethoscope_results` , ensure you stop `ethoscope_backup.service` before, and restart it when the backup is completed.

Installing the Ethoscope Software from Scratch

Warning: this section of the manual is for **experts only**. Unless you know what you are doing, the best way to get a working ethoscope is to use our os snapshots as described here. That said, if you want to understand how the software runs on the ethoscope, this is the best way.

Getting the basic OS on the card

We use Archlinux as the main operating system for the ethoscope platform. The first part is to get Arch on the card. This procedure is explained here.

Customisation

Once you have the card ready. we can start putting the software we need and changing the configuration to have a working ethoscope.

Essential prerequisites

Before you start you will need:

- a wifi dongle in the pi2 (so we can access the node, pi3 has wifi on board)
- a working internet connection via ethernet cable (so we can download extra software)
- a node running at `192.169.123.1` see here
- a working `ETHOSCOPE_WIFI` network see here
- a screen and a keyboard plugged in the pi make things simpler

Extra packages

At this stage, you should have powered your pi and reach a login screen. The username *and* passwords are `root` .

We will install a few software we need (or may need in the future), using `pacman` , the package manager:

```
# update/upgrade
pacman -Syu --noconfirm
pacman -S base-devel git gcc-fortran rsync wget --noconfirm --needed
# video processing / image analysis tools
pacman -S opencv libcl eigen mplayer ffmpeg gstreamer gstreamer0.10-plugins mencoder -
-noconfirm --needed
# a desktop environment may be useful:
pacman -S xorg-server xorg-utils xorg-server-utils xorg-xinit xf86-video-fbdev lxde sl
im --noconfirm --needed
# utilities
pacman -S ntp bash-completion --noconfirm --needed
# check we have all the firmware
pacman -S raspberrypi-firmware{,-tools,-bootloader,-examples} --noconfirm --needed
# preinstalling dependencies will save compiling time on python packages
pacman -S ipython2 python2-pip python2-numpy python2-bottle python2-pyserial mysql-pyt
hon python2-cherrypy python2-scipy python2-pillow --noconfirm --needed
# mariadb (MySQL)
pacman -S mariadb --noconfirm --needed
# Emulate hardware clock in case ntp fails
pacman -S fake-hwclock --noconfirm --needed
#for setting up wireless/rooming
pacman -S wpa_supplicant ifplugd wpa_actiond --noconfirm --needed
pacman -S libev --noconfirm --needed
pacman -S watchdog macchanger --noconfirm --needed
# we use pip to get picamera (python API to PiNoir)
pip2 install 'picamera[array]'
```

Environment variables

Here we define a few variables used in the rest of the installation

```
# mysql credentials
USER_NAME=ethoscope
PASSWORD=ethoscope
DB_NAME=ethoscope_db
# where ethoscope saves temporary local files (e.g. videos)
DATA_DIR=/ethoscope_data
# where to install and find our software
TARGET_GIT_INSTALL=/opt/ethoscope-git
UPDATER_LOCATION_IN_GIT=scripts/ethoscope_updater
UPSTREAM_GIT_REPO=https://github.com/gilestrolab/ethoscope.git
TARGET_UPDATER_DIR=/opt/ethoscope_updater
BARE_GIT_NAME=ethoscope.git
# network stuff
NETWORK_SSID=ETHOSCOPE_WIFI
NETWORK_PASSWORD=ETHOSCOPE_1234
#ip addresses
NODE_SUBNET=192.169.123
NODE_IP=$NODE_SUBNET.1
```

Wireless connection

This operation creates a `/etc/netctl/wlan0` profile file. It is used for communication to the node, not the internet.

```
echo 'Description=ethoscope_wifi network' > /etc/netctl/wlan0
echo 'Interface=wlan0' >> /etc/netctl/wlan0
echo 'Connection=wireless' >> /etc/netctl/wlan0
echo 'Security=wpa' >> /etc/netctl/wlan0
echo 'IP=dhcp' >> /etc/netctl/wlan0
echo 'TimeoutDHCP=60' >> /etc/netctl/wlan0
echo "ESSID=$NETWORK_SSID" >> /etc/netctl/wlan0
echo "Key=$NETWORK_PASSWORD" >> /etc/netctl/wlan0
```

Wired connection

This writes another profile file: `/etc/netctl/eth0`. It can be used on the intranet, but also for the internet (e.g. if you want to update software).

```
echo 'Description=eth0 Network' > /etc/netctl/eth0
echo 'Interface=eth0' >> /etc/netctl/eth0
echo 'Connection=ethernet' >> /etc/netctl/eth0
echo 'IP=dhcp' >> /etc/netctl/eth0
```

NTP

The pi does not have a hardware clock, so time will be reset/stopped when it turns off. One way to have accurate time is to use Network Time Protocol. Since pis are likely to only have access to local intranet, we use the node and an NTP server. Let us assume that there could be several nodes with IP addresses from 1 to 5.

```
echo "server $NODE_SUBNET".1 > /etc/ntp.conf
echo "server $NODE_SUBNET".2 >> /etc/ntp.conf
echo "server $NODE_SUBNET".3 >> /etc/ntp.conf
echo "server $NODE_SUBNET".4 >> /etc/ntp.conf
echo "server $NODE_SUBNET".5 >> /etc/ntp.conf
echo 'server 127.127.1.0' >> /etc/ntp.conf
echo 'fudge 127.127.1.0 stratum 10' >> /etc/ntp.conf
echo 'restrict default kod limited nomodify nopeer noquery notrap' >> /etc/ntp.conf
echo 'restrict 127.0.0.1' >> /etc/ntp.conf
echo 'restrict ::1' >> /etc/ntp.conf
echo 'driftfile /var/lib/ntp/ntp.drift' >> /etc/ntp.conf
```

Network daemons

```
systemctl disable systemd-networkd
ip link set eth0 down
# Enable networktime protocol
systemctl start ntpd.service
systemctl enable ntpd.service
systemctl enable fake-hwclock fake-hwclock-save.timer
systemctl start fake-hwclock
# Setting up ssh server
systemctl enable sshd.service
systemctl start sshd.service
```

```
#setting up wifi
netctl-auto enable wlan0
netctl-auto start wlan0
systemctl enable netctl-auto@wlan0.service
systemctl start netctl-auto@wlan0.service
systemctl enable netctl-ifplugd@wlan0.service
systemctl start netctl-ifplugd@wlan0.service
netctl enable eth0
netctl start eth0
systemctl enable netctl@eth0.service
systemctl start netctl@eth0.service
systemctl enable netctl-ifplugd@eth0.service
systemctl start netctl-ifplugd@eth0.service
#device service
```

At this stage, you may want to reboot and check that you have both `eth0` and `wlan0` working (i.e. use `ip a`). If you do, **do not forget to redefine our environment variables!**

MySQL

Ethoscope saves real time tracking data to a MySQL database.

```
mysql_install_db --user=mysql --basedir=/usr --datadir=/var/lib/mysql
systemctl start mysqld.service
systemctl enable mysqld.service
mysql -u root -e "CREATE USER \"$USER_NAME\"@'localhost' IDENTIFIED BY \"$PASSWORD\""
mysql -u root -e "CREATE USER \"$USER_NAME\"@'%' IDENTIFIED BY \"$PASSWORD\""
mysql -u root -e "GRANT ALL PRIVILEGES ON *.* TO \"$USER_NAME\"@'localhost' WITH GRANT
 OPTION";
mysql -u root -e "GRANT ALL PRIVILEGES ON *.* TO \"$USER_NAME\"@'%' WITH GRANT OPTION"
;
```

We can speed up MySQL queries by adding, under the [mysqld] section `skip-name-resolve` in the MySQL configuration file.

```
nano /etc/mysql/my.cnf
```

In addition, you may want to increase the memory allocation:

```
innodb_buffer_pool_size = 128M
innodb_log_file_size = 32M
innodb_log_buffer_size = 50M
innodb_flush_log_at_trx_commit = 1
innodb_lock_wait_timeout = 50
innodb_file_per_table=1
```

Getting the ethoscope software

```
git clone git://$NODE_IP/$BARE_GIT_NAME $TARGET_GIT_INSTALL
cd $TARGET_GIT_INSTALL
# this allor to pull from other nodes
for i in $(seq 2 5); do git remote set-url origin --add git://$NODE_SUBNET.$i/$BARE_GI
T_NAME; done
git remote set-url origin --add $UPSTREAM_GIT_REPO
git remote get-url --all origin
cd $TARGET_GIT_INSTALL/src
```

If you are not going to use the master branch, you should check out to your default branch. For instance:

```
git checkout dev
```

Then, we install the package with `pip` (`[device]` means we install optional deps for the device):

```
pip2 install -e .[device]
```

We copy our own services to `systemd` service list:

```
cp $TARGET_GIT_INSTALL/scripts/ethoscope_device.service /etc/systemd/system/ethoscope_
device.service
```

We move the updater webserver out of the ethoscope, so the updated does not update (/break) itself:

```
cp $TARGET_GIT_INSTALL/$UPDATER_LOCATION_IN_GIT $TARGET_UPDATER_DIR -r
cd $TARGET_UPDATER_DIR
cp ethoscope_update.service /etc/systemd/system/ethoscope_update.service
```

Now we can enable all:

```
systemctl daemon-reload
systemctl enable ethoscope_device.service
systemctl enable ethoscope_update.service
```

Boot config file

We ensure boot config will allow us to work with the pi camera

```
echo 'start_file=start_x.elf' > /boot/config.txt
echo 'fixup_file=fixup_x.dat' >> /boot/config.txt
echo 'disable_camera_led=1' >> /boot/config.txt
echo 'gpu_mem=256' >> /boot/config.txt
echo 'cma_lwm=' >> /boot/config.txt
echo 'cma_hwm=' >> /boot/config.txt
echo 'cma_offline_start=' >> /boot/config.txt
echo 'Loading bcm2835 module'
echo "bcm2835-v4l2" > /etc/modules-load.d/picamera.conf
```

Failure tolerance

It is possible that power, SD card or any other hardware inexplicably fails whilst tracking. For this reason, we can set up the watchdog timer, so that the pi will restart itself in the case of freezes.

```
echo "bcm2708_wdog" | sudo tee /etc/modules-load.d/bcm2708_wdog.conf
sudo systemctl enable watchdog
```

A small disk write test can be created:

```
mkdir /etc/watchdog.d/
echo '#!/bin/bash' > /etc/watchdog.d/write_test.sh && chmod 755 /etc/watchdog.d/write_
test.sh
echo 'sleep 5 && touch /var/tmp/write_test' >> /etc/watchdog.d/write_test.sh && chmod
755 /etc/watchdog.d/write_test.sh
```

We can add a few things to the config file (`nano /etc/watchdog.conf`):

```
max-load-1 = 24
watchdog-device = /dev/watchdog
realtime = yes
priority = 1
```

A good documentation is provided here

Last touches

There is an issue with some wireless dongles that go to idle if unused, but then become really hard to reach. We can disable power management for these dongle:

```
echo 'options 8192cu rtw_power_mgnt=0' > /etc/modprobe.d/8192cu.conf
```

We can save some sdcard io by using a ramdisk for temporary storage:

```
echo 'tmpfs /tmp tmpfs defaults,noatime,mode=1777 0 0' >> /etc/fstab; cat /etc/fstab
```

Card identity

We want to change three files that give this card a unique identity:

- `machine-id` is a long chain of characters that should be unique for any machine in the world
- `machine-name` is a human-friendly name for the machine (e.g. `ETHOSCOPE_001`)
- `hostname` is the name of the machine on the network (for the router) (e.g. e001)

```
cd /etc
nano machine-id machine-name hostname
```

Snapshot of the OS

At this point, it makes sense to back-up your work by making a snapshot of the OS. So, put the sd card in a computer.

Let us assume you have your root partition in `/mnt/root/` and boot in `/mnt/boot/` . Your resulting snapshot will live be `/tmp/ethoscope_os_yyymmdd.tar.gz` .

```
cd /mnt/root/
cp -r ../boot/* ./boot/
sudo tar -zcvpf /tmp/ethoscope_os_yyyymmdd.tar.gz *
rm ./boot/* -r
```

Modules

The ethoscope comes along side a series of modules that allow experimentalists to perform feedback-loops. This section documents modules from the point of view of the "builder".

Optomotor Module

The optomotor is an ethoscope module that allows users to deliver both mechanical (tube rotation) and optical (LED) stimuli to fruit flies hosted in individual glass tubes.

The original onshape repository for the optomotors lives here.

The assembled module will look like this:

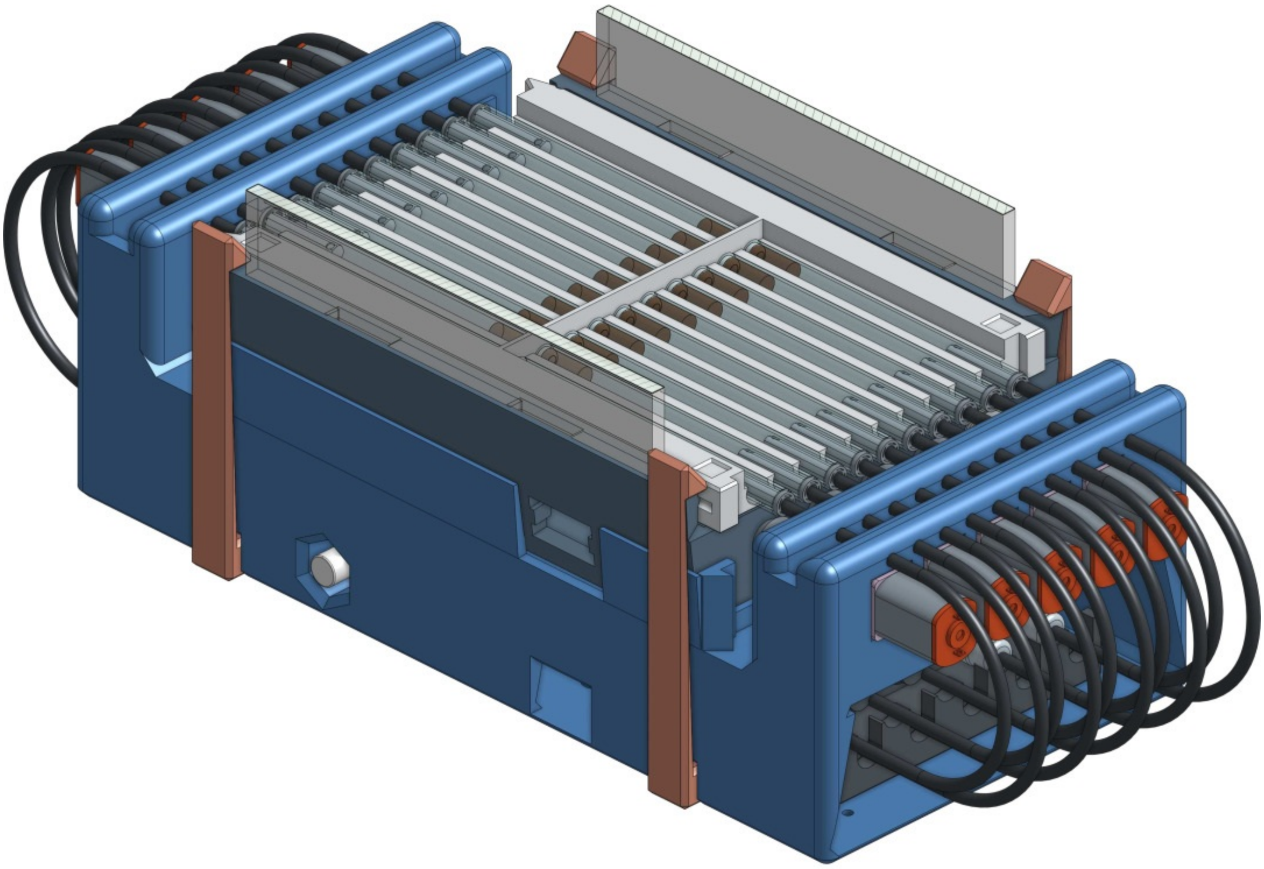

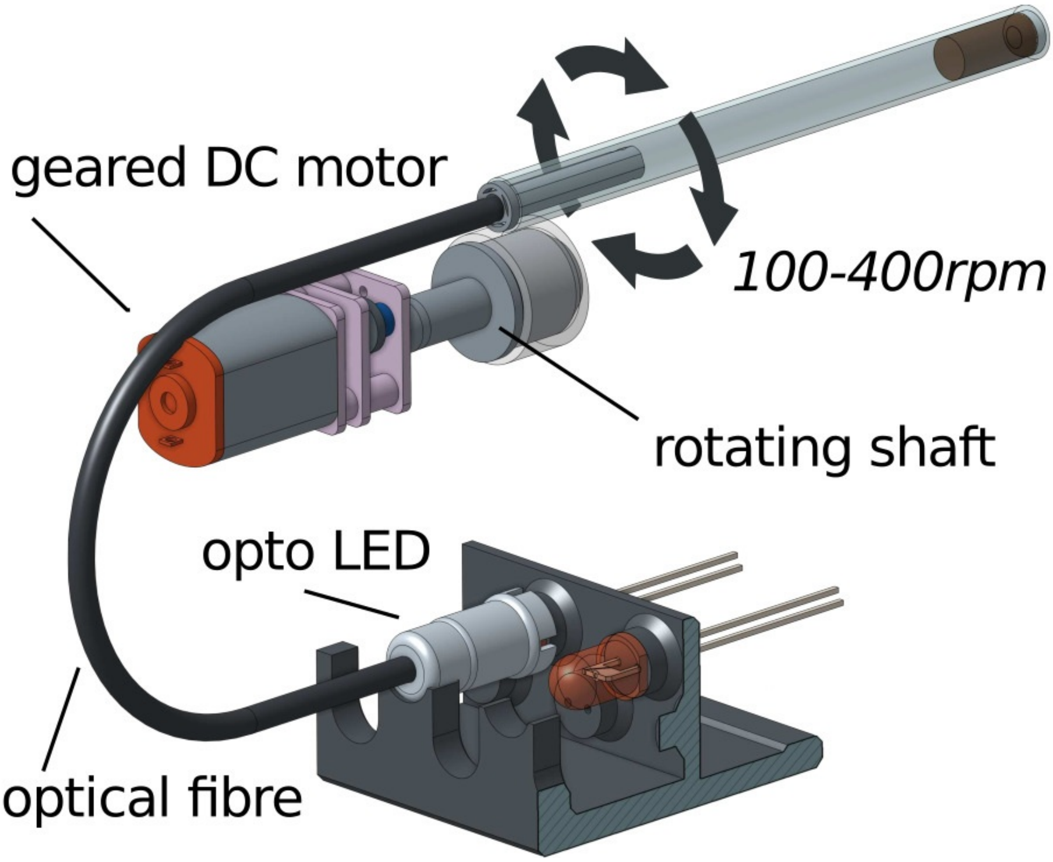

Conceptually, the module contains three distinct parts:

- "main", the mandatory components
- "opto", only needed for optical stimulus delivery
- "motor", only needed to rotate tubes

In this document, we describe how to build both opto and motor parts.

List of components (BOM):

Piece	Part	Quantity	Supplier	Reference	Price (GBP)
Main box	main	1	3d printed	onshape	0
Box clips	main	2	3d printed	onshape	0
Shafts	main	10	3d printed	onshape	0
LED Panels	main	2	3d printed	onshape	0
USB A to micro cable (50cm)	main	1	Farnell	Farnell 2506103	5
USB A (Power supply)	main	1	RS	RS 888-8811	5
Screws (M2.6 x 8mm)	main	2	RS	RS 664-4956	0
Screws (M1.2 x 6mm)	main	2	RS	RS 418-7218	0
Arduino micro	circuitry	1	RS	RS 779-8864	15
TLC5947 PWM Driver	circuitry	1	Adafruit	Adafruit 1429	15
Push button	circuitry	1	RS	RS 133-6502	2.5
PCB Headers (2 x 3)	circuitry	8	RS	RS 251-8137	0.2
Right angle 6 way socket	circuitry	3	RS	RS 543-7750	0.3
PCB Header (1 x 6)	circuitry	3	RS	RS 681-2994	0.4
Female 2 pins crimps	circuitry	20	Farnell	Farnell 1593506	0.24

Crimps contact	circuitry	40	Farnell	Farnell 1593529	0.06
Ribbon cable	circuitry		RS	RS	0
Resistor (1.15k)	circuitry	2	RS	RS 754-8691	0.25
N20 gear motor	motor	10	???	various	4
Silicon tubing 6x9 mm	motor	1	VWR	various	2
Filter Box	opto	1	3d printed	onshape	0
IR filters	opto	1	TODO	onshape	0
Light Guides (8in)	opto	5	RS	RS 890-5782	2.5
LED Fibre Optic Couplers	opto	10	Mouser	Mouser THR_5_22	0.6
630 nm LEDs	opto	10	RS	Farnell 1593506	0.15

Main

The idea is to prepare two electronic boards : Arduino micro and TLC5943, screw them to the bottom of the main box and connect them to each other. Also, we add a push button for testing purposes.

Trimming the main box

The main box is a large print with a lot of support, and it is easier to trim it before putting anything sensitive inside. Importantly, if you intend to add motors, **ensure you remove all plastic from the cavity that will hold them**. Indeed, tiny bits of plastic could end up inside the gear box.

Arduino board

This is the "master" board that processes input from the computer and coordinate the stimulus delivery.

1. Solder a 4-pin header (cut a 6-pin header two, 4+2) between pins 3 and 6 (see picture below)
2. Solder a 2-pin header between pins 11 and 12

3. Screw the board in place using four 1.2mm diameter screws.

4. Plug the USB micro cable to the board. Then compile and upload the firmware (you need some degree of familiarity with the Arduino build process for that). The code for this firmware lives here.

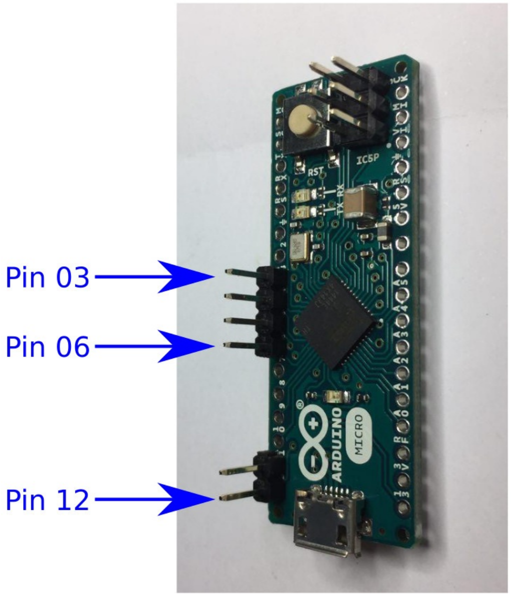

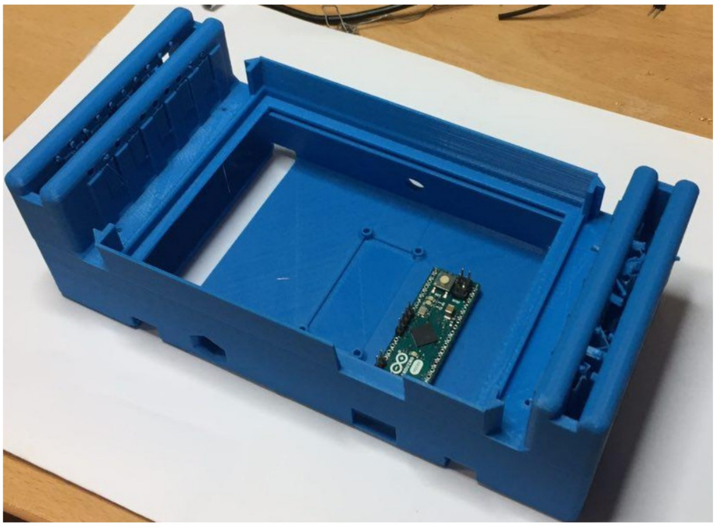

TLC5943

This board is the "slave". It is ultimately responsible of turning on/off components (LED/motors).

1. Solder all (i.e. 8) the 6x2 headers (see picture below)

2. Solder the 2 6x1 headers on both extremities of the board

3. Solder the 1.15kΩ resistor

4. Screw the board in place -- using the 2.6mm diameter screws

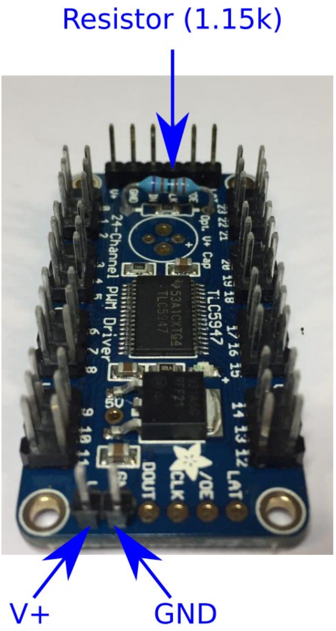

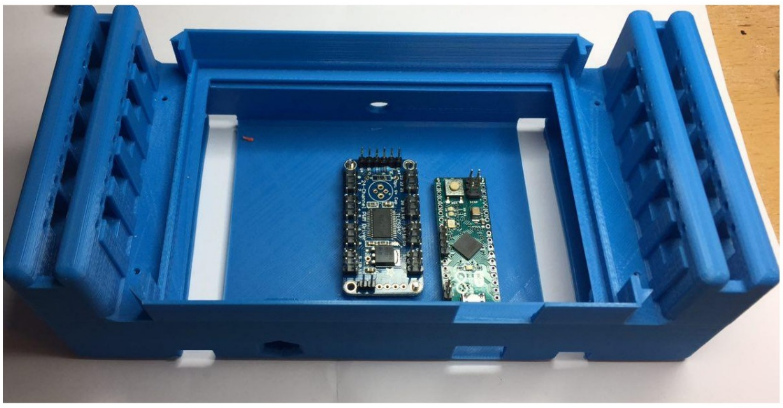

Board connector

The connections between the two above boards has to be very robust at all time.

To build the connector (picture below), we use **80mm** of **6-way** ribbon cable. Four of the wires connect the two boards, whilst two bring external power to the TLC5943.

1. Strip all six wires on one end only.
2. Squeeze each wire on a different slot of the 6-way female connector
3. Solder all six wires (this is a bit of a hack, but it works well if you don't have the tool for these connectors)
4. Cut the two wires on the right so that the are only **20mm** long
5. Strip and solder the four remaining wires to the second female connector (the wire must obviously have the same order and position on the second connector -- e.g. in the picture, yellow is on the left of both connectors)
6. Strip the two remaining short wires and add a 2-pin female socket (using two crimps connectors).

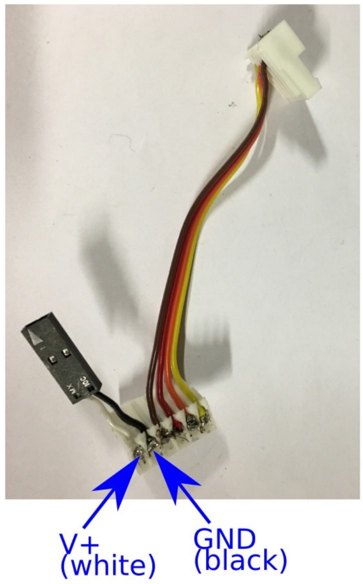

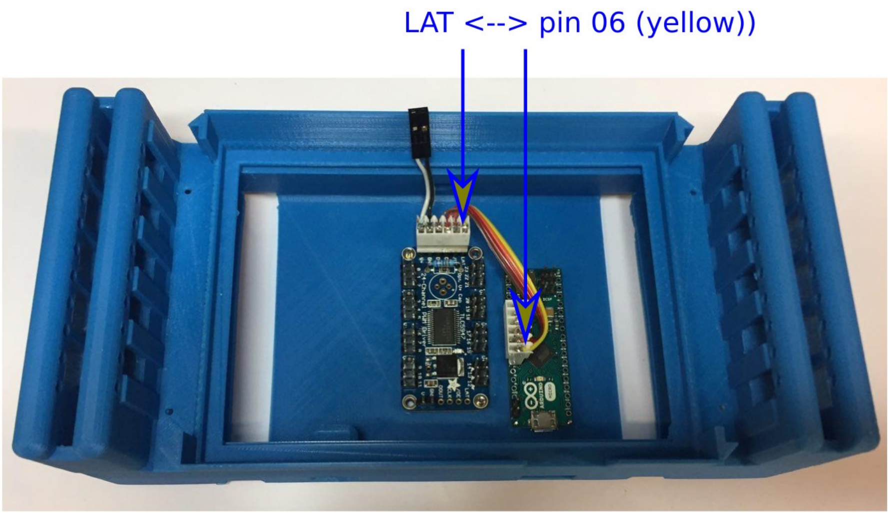

Push button

To assemble the push button:

1. Cut a **150mm**, **3-way** (green, black, white) ribbon cable (picture below)
2. Strip the three wires on one side only, and **preload some heat shrink** before you solder
3. Solder the V+ (white) cable directly to a pin of the push button (it doesn't matter which one)
4. Solder the digital out (green) pin directly to the other pin
5. Solder the ground (black) to the same pin as the digital out, but **through the 1.15kΩ resistor**
6. Retract the heat shrink
7. Cut the ground and V+ wires to **80mm**
8. Use crimps connectors to put ground and V+ in the same 2-pin female socket
9. Use crimps connector to put the digital out wire on a 2-pin female connector (one of the slot will be empty, but do put a crimps connector inside anyway as this makes a better mating between connectors)
10. Bundle all the wires/resistor on the push button end with some larger heat shrink or electrical tape

11. Pass the assembled push button **from the inside of the box to the outside** through the small hexagonal cavity. Then lock it using the hexagonal screw from the outside. Admittedly, it is not very easy to spin the push button at this stage, but it will work with some determination!

12. Connect the **V+ and ground pins** to the corresponding pins on the **TLC5943** board

13. Connect the **digital out** wire on **pin 12** of the arduino

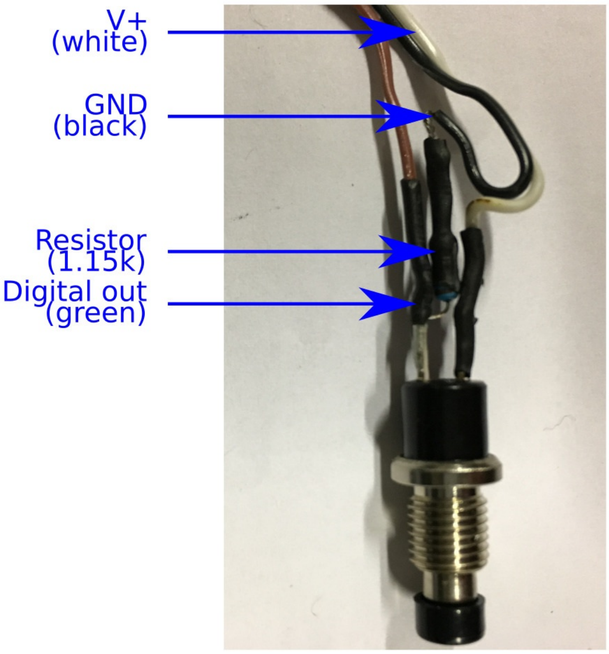

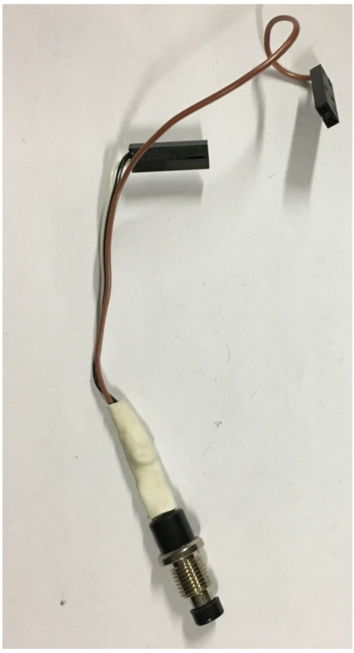

Power cable

1. Cut the small end of the strip the long USB cable
2. Strip red(V+) and black(GND) cables, cut the others
3. Put some heat shrink around both cables
4. Solder each to a pin of a 2x1 male header
5. Process the heat shrink
6. pass the assembled cable through the circular hole in the back of the box
7. Plug it in the corresponding pins, in the board connector
8. Put a cable tight inside and the outside the box, around the power cable, to prevent stress/disconnection

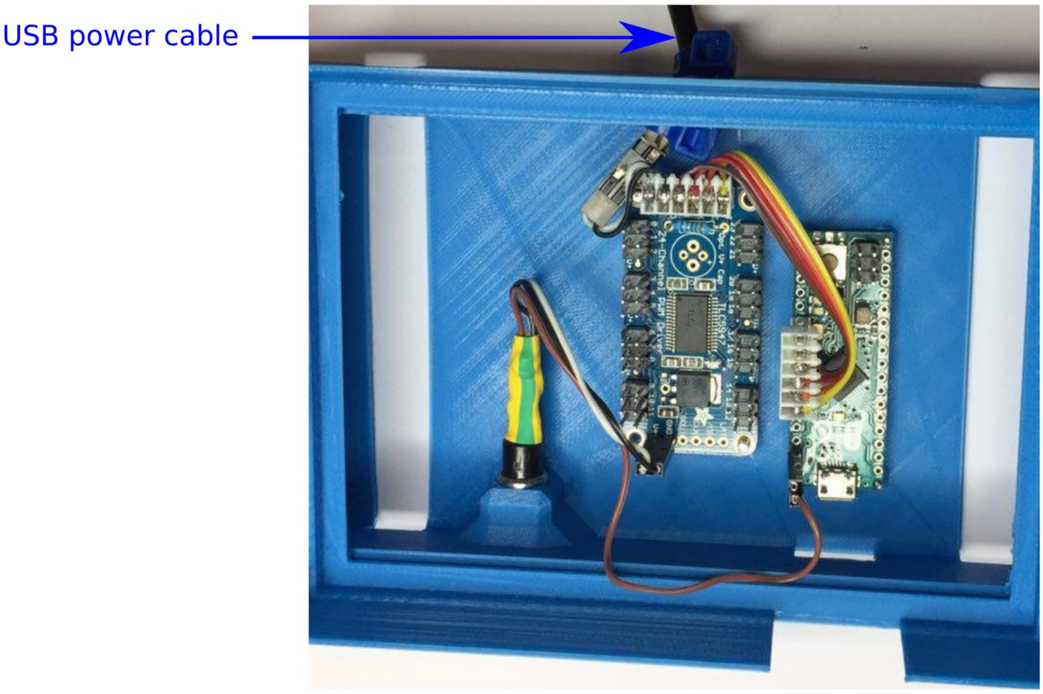

Opto

In order to deliver light to specific tubes, we will make an array of LEDs and connect the LED, with optic fibres, to individual tubes.

Each LED is fitted inside a panel, their are two panels (left and right), each holding five LEDs per machine. The panels are held in position by the "clips"

1. Trim the two panels, so they fit without forcing in the clips. Also remove any support structure on the LED panels that could have been added by your 3d printer.
2. Set the LEDs so that the **+ side is on the left** (see picture)
3. Cut all the LED legs to 3mm or so
4. Prepare (strip bot ends) as many (10) **75mm**, **2-way** ribbon cables
5. Put a 2-way female socket on one end (using the crimps connectors)
6. Put some heat shrink (5mm should do) on both wires on the other side of the cable.
7. Solder the LEDs. In this document, by **convention**, we use **violet for -** and **light grey for +**
8. Process the heat shrinks

Motor

Each motor has its own individual slot, so we can just make 10 motors and fit them on the box. **Ensure you remove all plastic from the motor slots, so that gears don't get stalled**

1. Prepare (strip bot ends) ten **150mm**, **2-way** ribbon cables
2. Put a 2-way female socket on one end (using the crimps connectors)
3. Put some heat shrink (5mm should do) on both wires on the other side of the cable.
4. Solder the motors. In this document, by **convention**, we use **green for -** and **blue for +**. Each motor will have a small "+" written by the corresponding pole.
5. Pass each motor through its slot in the main box
6. Put its shaft (3d printed small orange part) to each motor
7. Add a ring of silicon (i.e. a section of tube around **5mm long**) to each shaft -- this ensure maximal friction

Putting things together

Plugging the components to the TLC board.

By convention, **motors are always on even numbers** and **LED, on odd ones**. In addition, to avoid wires crossing, we map the components the pin facing them, which does not match the numerical order.

So our topology is:

```
MOTOR 01 -> PIN 00 | PIN 23 <- LED 06
LED 01 -> PIN 01 | PIN 22 <- MOTOR 06
MOTOR 02 -> PIN 02 | PIN 21 <- LED 07
LED 02 -> PIN 03 | PIN 20 <- MOTOR 07
MOTOR 03 -> PIN 04 | PIN 19 <- LED 08
LED 03 -> PIN 05 | PIN 18 <- MOTOR 08
MOTOR 04 -> PIN 06 | PIN 17 <- LED 09
LED 04 -> PIN 07 | PIN 16 <- MOTOR 09
MOTOR 05 -> PIN 08 | PIN 15 <- LED 10
LED 05 -> PIN 09 | PIN 14 <- MOTOR 10
```

Finishing touches

1. Trim the two clips and fit them on the bottom of the box
2. Lock the clips by sliding the LED panel by the side of the box (if you are not using LEDs,

you can simply put panels with no LEDs)

3. Plug both power supply for the TLC and serial connection for the Arduino
4. Press the push button for 5s and release. Check that every component is being activated, in the right order
5. After you have tested the machine, you can decide to mask the various LEDs on both boards if you perform experiments where you don't want any external light

Using Ethoscopes, What You Need to Know

What is an Ethoscope

Ethoscopes are small devices based on Raspberry pi microcomputers. They look like this:

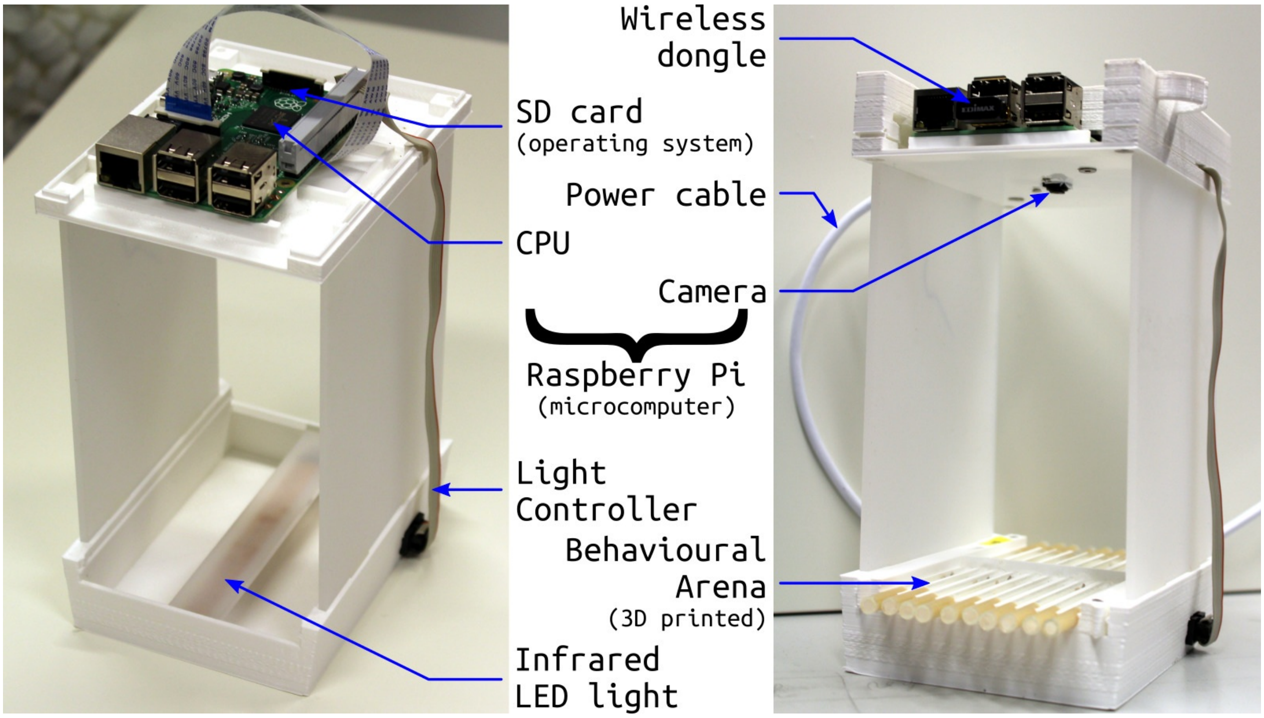

Each device is effectively a small computer. It is important to understand that they will **perform the video tracking in real time and on their own**.

Arenas

The arena is the container where experimental animals will be held. **It defines your experimental paradigm more than anything**, so you are quite likely to use different arenas to ask particular questions. This is an example of an arena that can hold 20 fruit flies in 3x5x65 mm tubes:

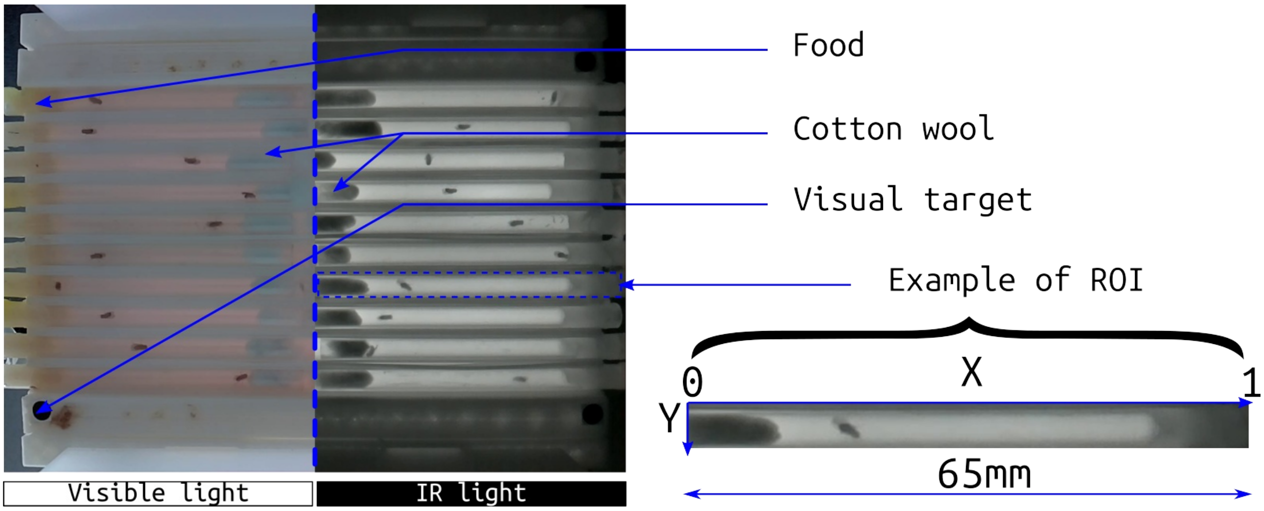

I split the image to show day (left) and night (right) vision. A few important things about arenas:

- An arena contains **Regions Of Interest** (ROIs).
- Targets (black circles) are used to automatically define ROIs. Therefore, they need to be **always visible and sharp**.
- ROI definition is performed only when tracking starts, so **you cannot move your arena during an experiment**.
- The **top-left corner** of the image is always the corner with **no target**.

The Ethoscope Platform

The whole point of ethoscopes is that you can have many running independently. However, you need a way to start/stop/orchestrate them all. The platform has a central computer, that we call **The Node** dedicated to this task. Here is a simple diagram showing how the platform works:

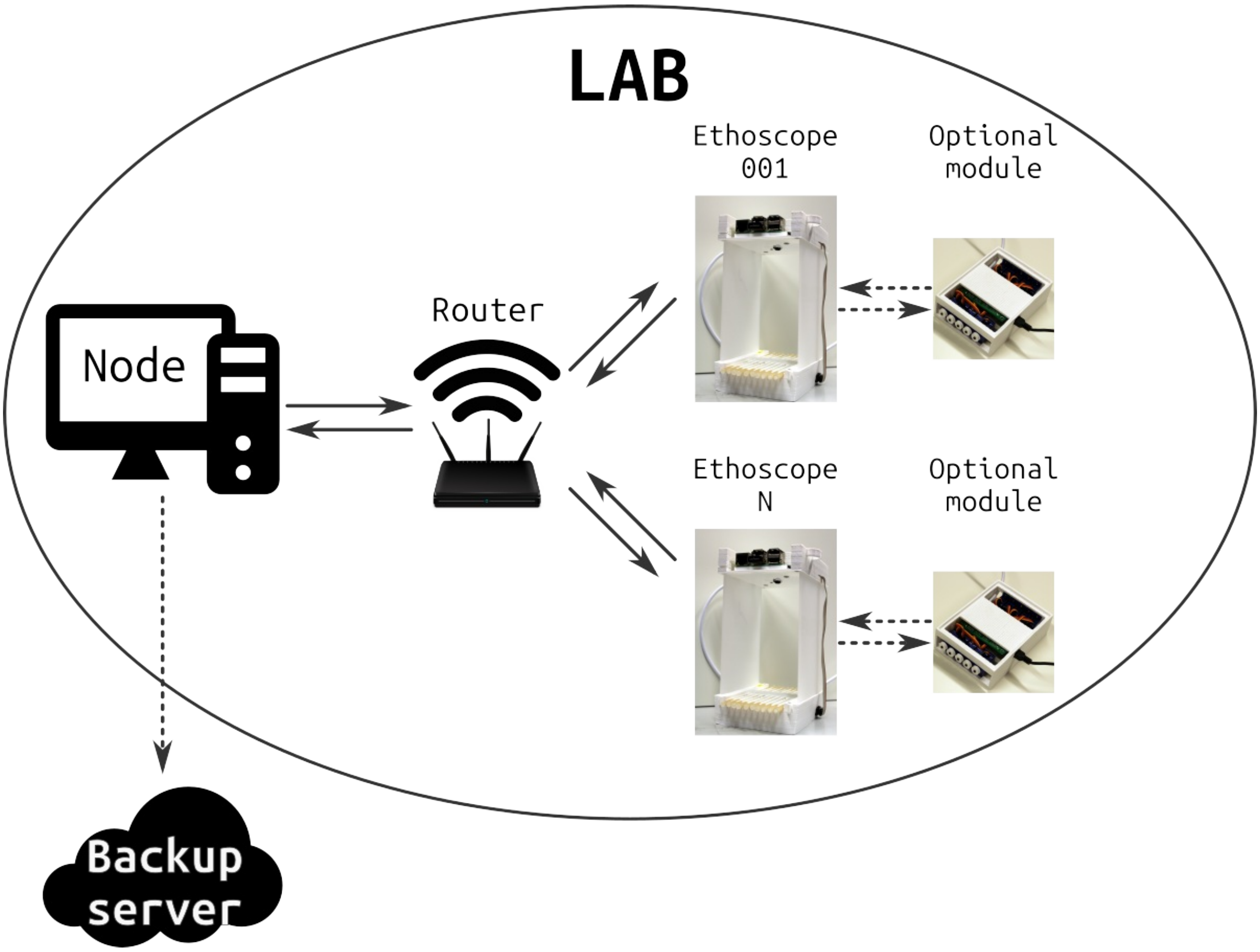

It provides a **web interface** that:

- **Lists** all detected devices
- **Controls** a specific device (Select/Preview/Start/Stop)
- Silently **synchronises** and centralises the data from each device

The node runs all of these through a website, so you can simply control devices from your web browser as long as you connect to the same network and visit the node's address ("http://node").

Running a Tracking Experiment

As you open the node home page (http://node) you see an overview of the devices/platform. Here is an example in our lab:

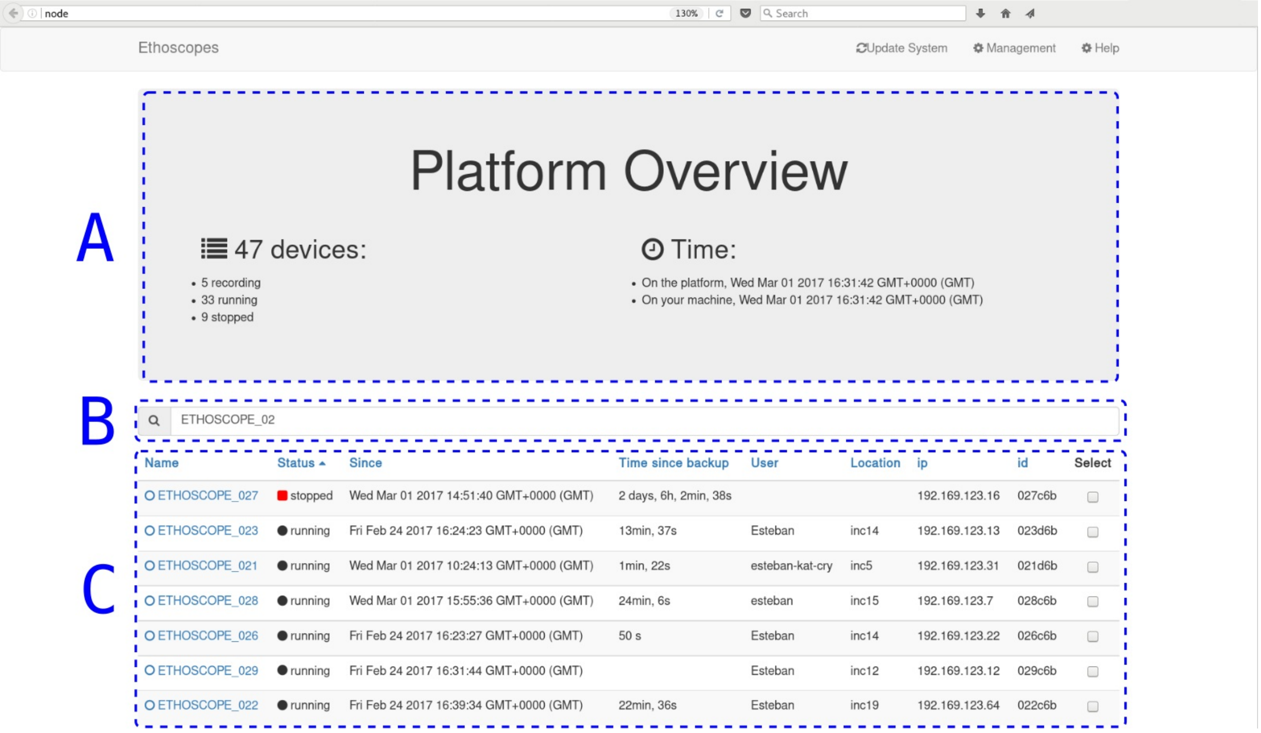

I have annotated the three important regions:

A. Gives general information about the platform. Specifically, how many devices are detected, and what they are doing. In addition, it shows the time. The time on the platform (i.e. the node) and the time on the machine you are accessing the node from (e.g. a tablet/phone) may be different see the time section.

B. A search box to filter devices in C. It filters all fields. Here, I wrote `"ETHOSCOPE_02"`, so it lists names starting with `"ETHOSCOPE_02"` (that is devices number [20-29]). You could also write `"stopped"` and list all `"stopped"` devices or filter by user... It saves time and prevents errors when you have many devices.

C. a table listing all devices. It shows how long each device has been in its status ("stopped"/"running"/...), when their data was backed up last, who is the current user and where it is (this is defined manually when starting)... `ip` and `id` fields are technical details mostly for the maintainers.

As you **click on a device name**, you enter the control page for this machine. Here, you can, amongst other things, **start tracking**, **record video** and **power off** the machine.

Starting the Experiment

Before you start you experiment, you want to check:

- That **the machine is plugged and detected** by the network (it should appear as "stopped" on the home page)
- That **the arena is in place**, well illuminated, and **the targets are not occluded** by anything (e.g. wires)

When you click on start tracking, a "form" should appear:

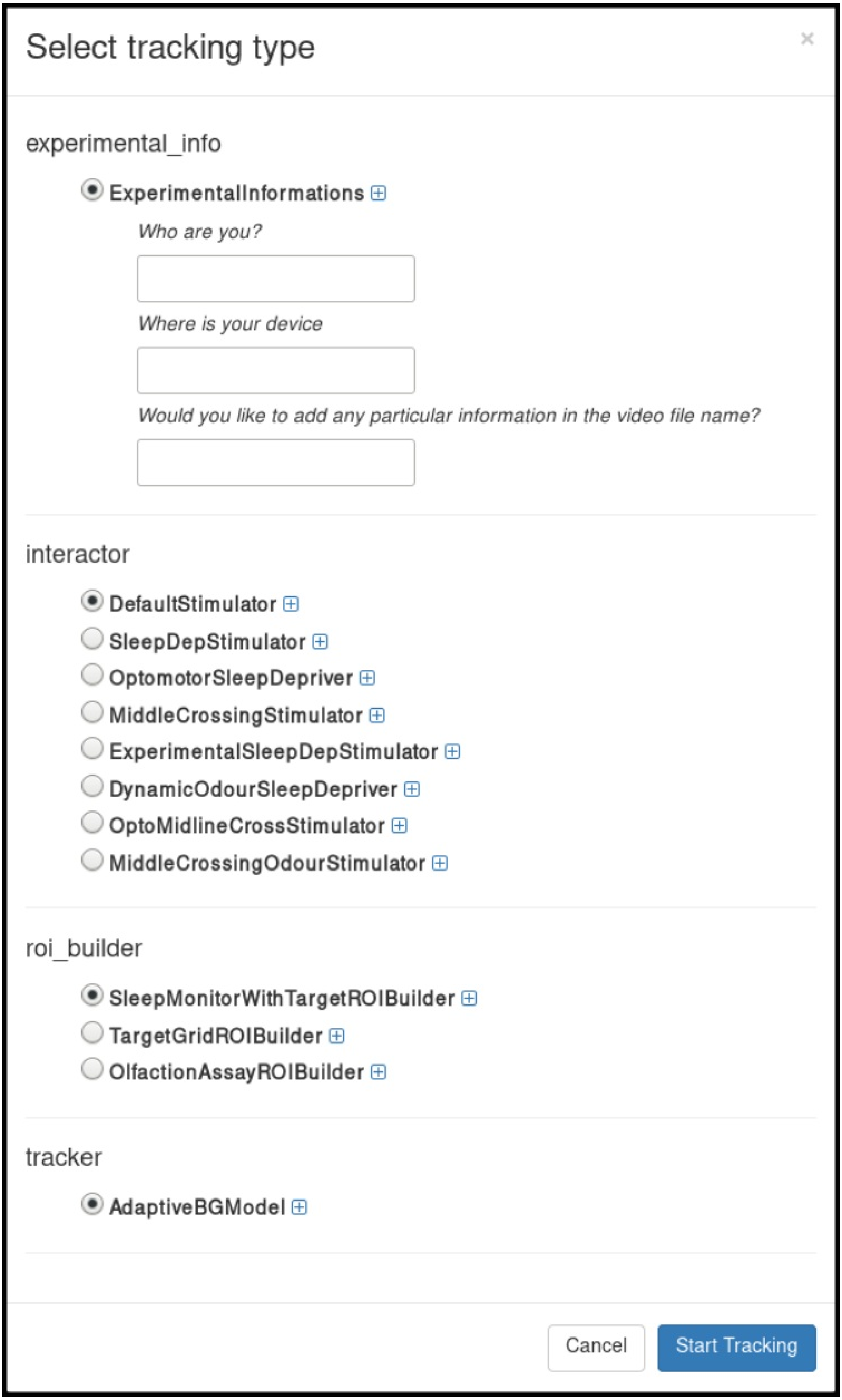
Select tracking type
×
experimental_info
ExperimentalInformations
Who are you?
Where is your device
Would you like to add any particular information in the video file name?
interactor
DefaultStimulator
SleepDepStimulator
OptomotorSleepDepriver
MiddleCrossingStimulator
ExperimentalSleepDepStimulator
DynamicOdourSleepDepriver
OptoMidlineCrossStimulator
MiddleCrossingOdourStimulator
roi_builder
SleepMonitorWithTargetROIBuilder
TargetGridROIBuilder
OlfactionAssayROIBuilder
tracker
AdaptiveBGModel
Cancel
Start Tracking

It has four parts:

- **Experimental information** -- The are used as metadata. It is important to fill them if you want to keep track of who is using devices, and where they are.
- **Interactor**/stimulator -- This field is only relevant when you use **feed back loop** modules. If you perform just tracking (no stimulus delivery), select the `DefaultStimulator` (which does nothing). The others rely on specific hardware modules.
- **ROI builder** -- This decides how to build the ROIs from the targets.
- **Tracker** -- which algorithm to use for tracking (only one available as of 01/03/2017)

In general, you can click on the " `+` " to display the description of an option/module.

Time in the Platform

Time is the most important variable when recording behaviour. It is therefore crucial that devices are on-time, and that we do not have "time-bugs".

How ethoscopes get the time

Since:

- Raspberry pis do not have a clock
- Even if they had a clock, it would drift over the months

We use Network Time Protocol this protocol to:

1. Synchronise the time from reference atomic clocks to the node
2. Synchronise all devices to the time of the node

Why we use UTC

The whole platform **always runs on UTC/GMT timezone**. When you analyse your data, or use a scheduler, you will therefore always refer to UTC instead of your local time. At first, this seems a bit counterintuitive, but it will save a **lot** of trouble. Otherwise, for instance, you could run through big issues if time changed from summer to winter time in the middle of an experiment (and this is only one example)! Therefore, when planning an experiment, you need to keep in mind that "ethoscope time" is UTC.

Error messages

If you start many machines, you will be confronted, sooner or later, with some errors messages. Most of the time, the user interface will report the error and try to help you to fix it.

This is an example of a reported error that was raised as tracking started:

Sorry there has been an error: Traceback (most recent call last): File "/opt/ethoscope-git/src/ethoscope/web_utils/control_thread.py", line 405, in run cam, rw, rois, TrackerClass, tracker_kwargs, hardware_connection, StimulatorClass, stimulator_kwargs = self._set_tracking_from_scratch() File "/opt/ethoscope-git/src/ethoscope/web_utils/control_thread.py", line 357, in _set_tracking_from_scratch raise e EthoscopeException: 'There should be three targets. Only 2 objects have been found'

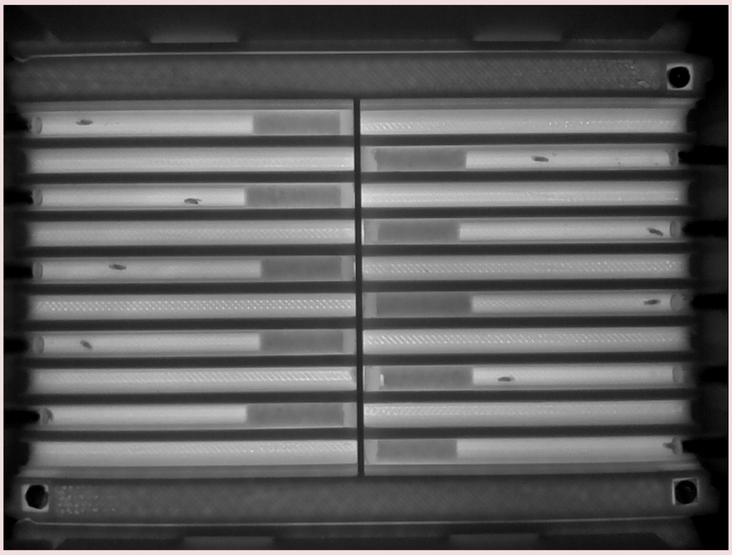

The error is reported alongside an image. **Take the time to read the error message**, especially the last statement. It says "There should be three **targets**. Only two objects have been found". So it is related to the detection of the black dots (targets) on the edge of the arena. When you take the time to look at the image, you will see that **the targets are not very circular** (they were not cut very well). As a result, they cannot be detected properly. In this case, you will need to put new, properly cut, targets.

The common type of errors are:

- Something went wrong because of the targets not being detected properly.
- You entered unexpected values in some of the forms (e.g. letters instead of numbers, fancy characters, a scheduler that is supposed to stop in the past...)
- The ethoscope cannot communicate with a module (see next section). This could mean the module is not powered or not connected to the ethoscope.
- More rarely, you will have a more obscure error for which you may need help from your

maintainer. When appropriate, rebooting the device can also help.

Modules

Sometimes, you will want to work with modules that can deliver stimuli in real-time feedback loops. Many things depend a lot on what module you will be using, and you should be aware of the specific requirements of your device. However, there are also general points:

- **Warm-up**, most modules will perform a "warm-up" round as you start tracking. This generally sends dummy stimuli in a set order. This allows you to check that stimuli delivery is working -- and do so in **the right order** (e.g. you have not mismapped ROIs).
- **Scheduler**, all modules will allow you to define a time window when to perform the dynamic stimulus delivery. For instance, often you will want a baseline of several hours or days, and then perform your feedback loop experiment. There is a user interface calendar allowing you to define time windows, but you can also write advanced time window in a simple format. See the scheduler documentation

Using schedulers to define when modules will perform their tasks.

When planning an experiment with ethoscope modules, you will often want to decide **when** to run a module. Schedulers allow you to specify this in a flexible manner. Importantly, this only controls optional hardware modules, not tracking. Tracking is only stopped when clicking on the "stop" button.

Default

When no value is entered, the default is to start the module immediately and stop it when the tracking is stopped.

General time range format

When doing something else than the default, you will need to enter text in the scheduler box (when starting from the web interface). A simple time range has the following structure:

```
DATE1 > DATE2
```

Where `DATE1` and `DATE2` are two dates that are formatted as `YYYY-MM-DD hh:mm:ss`. So, for instance, a valid time range could be `2016-04-01 21:00:00 > 2016-04-02 09:00:00`, which is 12h overnight.

Just like the for rest of the platform, **you want to use UTC/GMT, NOT local time** (see the time section)

Special cases

Simply entering one date:

```
DATE1
```

means "start at this date, and stop when tracking stops".

If the date is preceded by `>`:

```
> DATE2
```

it means "start now and stop at `DATE2` "

Advances uses

Sometimes, you will want to specify several valid time intervals when to apply your module. For instance, overnight interaction for two consecutive night (but nothing during the day).

You can set several time ranges by separating them with a `,`. For instance:

```
DATE1 > DATE2, DATE3 > DATE4
```

This means "apply the module between date 1 and date 2 and between date 3 and date 4 exclusively". This implies that the **date ranges do not overlap**.

This way, you can chain as many intervals as you want.

Maintaining the Platform

This part of the documentation provides a bit more technical background for users who would like to understand and administrate the platform. Lets start with a schematic:

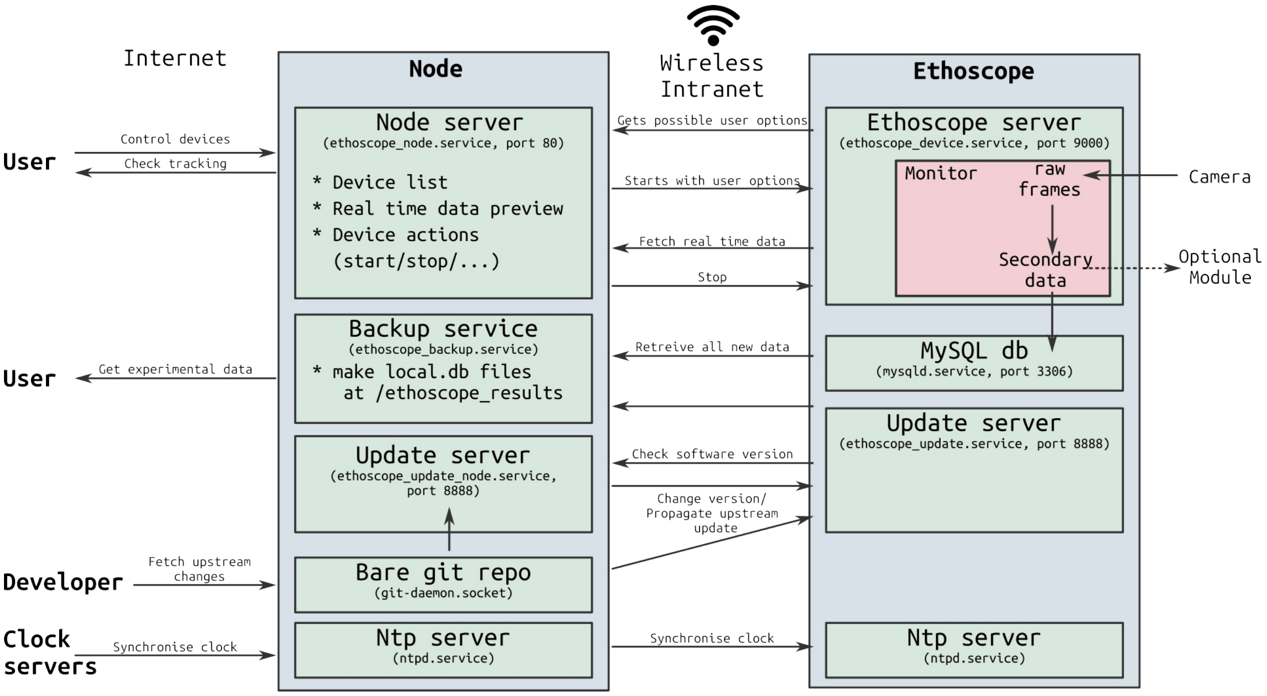

The three components, **node**, **wireless router** and **ethoscope** are of the system are represented here. Node and ethoscope run various "services" (custom or not), which are individually represented by green rectangles. Services are daemons) controlled by systemd.

As such, you can:

- Monitor them using `systemctl status <name-of-the-service>`.
- Stop/start/restart/enable/disable them `systemctl <instruction> <name-of-the-service>`.
- Examine their log files with `journalctl -u <name-of-the-service>`

Node

The node typically exists on the network with the ip `169.192.168.1`. It runs several services:

- A web server, `ethoscope_node.service`, which provides a user interface to detect, start, stop... ethoscopes, on port 80.
- A data synchronisation tool, `ethoscope_backup.service`, which saves locally data from all Ethoscopes. Ethoscope `.db` files are saved on `/ethoscope_results` periodically (typically every 5min).

- A video synchronisation tool, `ethoscope_video_backup.service` (not shown). It saves video chunks that were acquire
- An update tool `ethoscope_update_node.service` which allows users to update the software of the Node and of the Ethoscopes, on port 8888.
- A git server to mirror upstream updates from GitHub.
- An NTP server to serve time to all ethoscopes.

Ethoscopes

Each ethoscope runs their own:

- Webserver, `ethoscope_device`, on port 9000. It exposes status, tracking data, snapshot... This server is normally only accessed indirectly through the node server. The server can be queried to start/stop video tracking. Importantly, they analyse raw video themselves.
- `MySQL` database where all secondary data (e.g. shape and position of individual animals) resulting from the ongoing experiment are stored.

Router

The duty of the router is to:

- Allocate Dynamic IP addresses to detected Ethoscopes.
- Allocate a reserved IP for the Node.

Updating Ethoscopes

Regular update

Both node and ethoscopes run a custom python package that is maintained on a git repository: https://github.com/gilestrolab/ethoscope.

We anticipate that some issue will arise and that you will need to update ethoscopes. Instead or reburning every single card, we offer a tool to update the node and the ethoscopes from our upstream github repository.

1. Ensure your node can reach the internet
2. Open the update server (on port `8888`, e.g. `http://node:8888`)
3. Check you fetched the last version from github (the date of the last update will be displayed). you may need to reload the page.
4. Select the devices to be updated. Note that devices that are running cannot be updated (an error will show up if you try)!
5. Run "update"
6. Reload the page after a few seconds

Importantly, the update system does not update itself. It runs as an independent component.

Changing branch

We use a branching system to develop the ethoscope. This allows us to prototype new features. You can switch a device to a given upstream branch. This should facilitate colaboration and prototyping. Indeed, this allows us to have a specific experiment version of the software in the lab without compromising the stability of the main (`master`) version.

Restarting services

In the node

Important services are names in the administration section section.

You can use `systemctl` command line to check the status of a service on the node. For instance:

```
systemctl status ethoscope_node.service
```

To restart a service:

```
systemctl restart ethoscope_node.service
```

You may want/need to interupt a service in order to restart it later. For instance, if you want to tranfer the result files, you will need to interupt the ongoing backup, and restart it after all data is transfered:

```
systemctl stop ethoscope_backup.service
# do something ...
systemctl start ethoscope_backup.service
```

In a device

Ethoscope devices also have services. In order to access a device, you will need to open an ssh connection (unless you put a screen and a keyboard onto the raspberry pi). To do so, you will need to retreive the ip address. It should be listed on the ethoscope homepage.

Then you can run:

```
ssh alarm@192.169.123.XXX
```

Where `XXX` depends on your device (it is not the number of the device, but an IP address allocated randomly/DHCP). The pasword is `alarm` . If you need it, you can enter a super user shell using `su` . Now you are "inside" the pi, you can view/restart/... services the same

way. If your device is running, you need to know what you are doing as you could crash it and lose experimental data.

Getting the Data Out

Do Backups!

First of all, it is your responsibility to store your experimental data in several places. Ideally, you want the data to be in several computers that are in different buildings. You also want to have archives (snapshots) of the results that cannot easily be modified by anyone (e.g. cloud backup solutions).

File System

The Ethoscope system saves by default the data in two custom directories:

- `/ethoscope_results` -- the results of tracking experiments
- `/ethoscope_videos` -- raw video files (when recording)

Both directory have the same substructure:

`<machine_id>/<machine_name>/<datetime>/<file(s)>`

Where:

- `<machine_id>` is a unique identifier for the machine (e.g. `0001eeee10184bb39b0754e75cef7900`)
- `<machine_name>` is a human friendly name (e.g. `ETHOSCOPE_001`)
- `<datetime>` is a date and time formated as `YYYY-MM-DD_HH-MM-SS` (e.g. `2016-03-21_20-55-10`)

Then, each file inside the directory will be uniquely and unambiguously named:

For instance, we could have a file named `2016-03-21_20-55-10_0001eeee10184bb39b0754e75cef7900.db`. This redundancy means that if this file is sent to a collaborator, it preserves its uniqueness (plus we have some metadata in the file).

Example of Backups Script

This is the sort of scripts we use in the lab. You are welcome to take it and modify it. You will have to change things according to what sort of remote computer you save your data on (in the lab, we have our own NAS).

```
#!/bin/bash
# we ensure the backup service is stopped during backup, so we do not keep modifying f
iles
# during backup
backup_service=ethoscope_backup
result_dir=ethoscope_results/
target_prefix=/mnt/nas/auto_generated_data/
(
## we use flock to ensure no concurent backups
# see http://stackoverflow.com/questions/169964/how-to-prevent-a-script-from-running-s
imultaneously
# Wait for lock on /var/lock/.myscript.exclusivelock (fd 200) for 10 seconds
flock -x -w 10 200 || exit 1
systemctl stop $backup_service &&
sleep 30 &&
python2 /root/incremental_size_upload.py -s /$result_dir/ -d $target_prefix/$result_di
r/
Rscript /root/make_db_index.R
cp /$result_dir/index.txt $target_prefix/$result_dir/index.txt
sync
systemctl start $backup_service

) 200>/var/lock/.$backup_service.exclusivelock
```

Complementary scripts (cron table, fstab) are available on our labscript repository.

Fixing Devices

When you are using many microcomputers simultaneously, sometimes in constraining environment (e.g. humid conditions), for several weeks at a time, it is likely that some devices fail.

There are several sensitive parts to consider:

- wireless connection
- SD card
- MySQL database
- Ethoscope software
- Camera (e.g. disconnected or broken)

The procedure to diagnose most issue is to:

1. Restart the device
2. Try to innitiate tracking
3. Try to establish an SSH connection
4. Try to plug the device into a screen (in case the internet connection is broken)
5. Try to replace the SD card with a new one (it is good to have SD cards that are preformated for this purpose)

Ethoscope API

The ethoscope software is a python package that can run on arbitrary computers (not only raspberry pis). This can be used tracking animals from videos or for prototyping (e.g. developing new modules). The API is documented on readthedoc: docs latest docs dev

Rethomics

In order to process, summairsed and perform statistics on your tracking data, after your experiment is finished. We provide rethomics. As rethomics can also analyse tracking data from another platform, it has its own documentation.
