# Supplementary material for: Ethoscopes: An open platform for high-throughput ethomics
Source: PLoS Biol. 2017 Oct 19;15(10):e2003026. doi: 10.1371/journal.pbio.2003026 (PMC5648103; doi:10.1371/journal.pbio.2003026)
Supplement: S4 Text — (PDF) [file pbio.2003026.s005.pdf]

Real-time video tracking specifications

Overview

The position and angle of the camera were observed to be slightly different between devices. For this reason, prior to tracking, the first five frames of the video stream are stacked and used for automatic determination of the position of the Regions of Interest (ROIs). Briefly, the three black circles ('targets', shown in Fig. 3A of the main article) on the edges of the arena are detected using a recursive thresholding algorithm for blob detection (similar to [1]). Their position is then used to scale and rotate a hard-coded grid defining the ROIs.

In every subsequent frame, the sub-image defined by each ROI is processed separately in several steps (listed in figure 1). In summary, minimal pre-processing (mainly denoising) is first applied to the sub-image. Then, the sub-image is compared to the background model to identify candidate foreground pixels. Afterwards, connected components in the foreground are extracted as individual objects. If more than one object is detected, a statistical model of the foreground is used to keep only the most likely object. Foreground model is updated using the features of the – presumably valid – detected object. The background model is updated selectively. In addition, its learning rate is dynamically updated according to the ambiguousness of the result. Altogether, this procedure leads to detection of the sub-pixel position of an animal and allows computation of secondary variables such as velocity.

Since the computing power of the target devices (Raspberry Pis) is limiting, frame rate fluctuates according to the processing load and other unpredictable factors. In addition, several versions of the device (e.g. rPi2 *vs.* rPi3), may run at different speed. For these reasons, velocity was corrected in order to remain consistent at heterogeneous frame rates.

Preprocessing

First, the sub-image is converted to greyscale. Then, Gaussian blur with $\sigma = 1.2$ is applied for denoising. The intensity histogram of the original image src is scaled so that the resulting image dst has its mean $\overline{dst} = 128$:

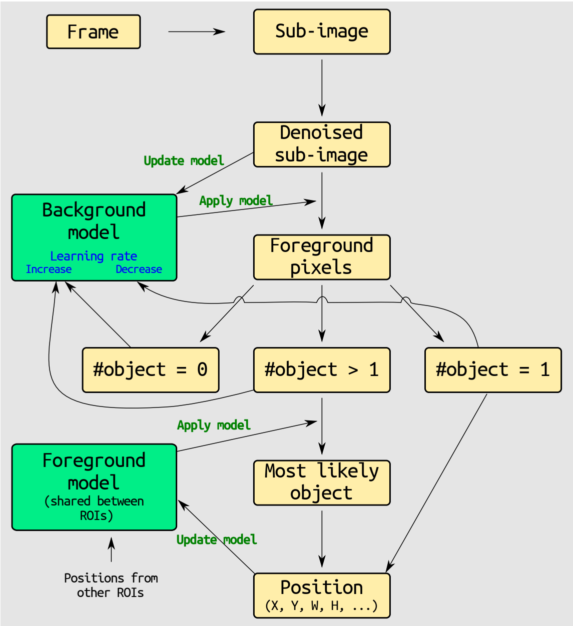

Figure 1: *Flowchart of the video tracking algorithm.* In each consecutive frame, ROIs are automatically defined and used to extract as many sub-images. They are then all processed separately. Firstly, simple denoising is applied. A background model is then used to extract foreground pixels. The preprocessed image is also used to update the background model. Objects (connected components) are detected in the foreground. When no object was detected, tracking is aborted. If more than one object is found, the foreground model is used to keep only the 'most valid' (*i.e.* likely) object. If only one object is found, it is considered to be valid. Features of the remaining valid object are computed and used to update the foreground model. The learning rate of the background model is decreased only when exactly one object was found (*i.e.* unambiguous match), and increased otherwise. The background is not updated in the neighbourhood of the foreground object (not shown in this figure).

$$dst_{x,y} = src_{x,y} \cdot \frac{128}{\overline{src}} \tag{1}$$

Background and foreground model

Background model

Background model is based on a classical weighted running average with a mask:

$$dst_{x,y} = \begin{cases} (1-\alpha) \cdot dst_{x,y} + \alpha \cdot src_{x,y} & \text{, if } mask_{x,y} > 0. \\ dst_{x,y} & \text{, otherwise.} \end{cases} \tag{2}$$

It was observed that the frame rate of devices was heterogeneous, depending on system load. Therefore, instead of implicitly assuming constant frame rate (*i.e.* α is constant), our background model accounts explicitly for the delay δt between two consecutive frames:

$$\alpha = 1 - exp(-\frac{2}{t_{1/2}} \cdot \delta t) \tag{3}$$

$t_{1/2}$ corresponds to the 'half-life' of the information in a pixel. The larger $t_{1/2}$, the longer it

takes for the model to learn. Importantly, the learning rate is dynamic $t_{1/2} \in [1, 100]s$ (see sub-section).

Foreground features

At each frame, the preprocessed image (src) is subtracted from the background model (bg) and thresholded with $thr = 20$:

$$dst_{x,y} = \begin{cases} 1 & \text{, if } bg_{x,y} - src_{x,y} > thr \\ 0 & \text{, otherwise.} \end{cases} \tag{4}$$

Then, connected components are located. If none are present, tracking is simply aborted. If more than one object is found, the foreground model (see next sub-section) is used to select only the most likely object. When exactly one object is detected, it is considered as 'unambiguous' foreground and used immediately.

Several primary features such as XY position, orientation, width, height, area and average pixel intensity are computed on the single resulting foreground object. In order to obtain an accurate measurement of velocity, subpixel XY position was computed using greyscale image moments as opposed to simply using binary moments.

Foreground model

A vector of three features – $log_{10}(area)$, height and average pixel intensity – describing the detected foreground object is stored in a feature matrix M. The likelihood $\mathcal{L}$ of candidate foreground objects, with a vector of feature X, is then computed based only on marginals, assuming a Gaussian distribution for the three features. For every column j, we have,

$$L_j = \frac{1}{\sigma_j \sqrt{2\pi}} e^{-\frac{(X_j - \mu_j)^2}{2\sigma^2}} \quad , \tag{5}$$

where μ_j and σ_j are the average and the standard deviation of the feature j, respectively.

Then, we have

$$\mathcal{L} = \prod_j L_j \ .$$

Importantly, the foreground model is shared between all ROIs, which assumes relative feature homogeneity between foreground objects. When tracking visually similar animals (*e.g.* fruit flies of approximately the same size), this assumption is expected to hold.

Updating background and foreground models

The background model is systematically updated for each frame, but its half-life is set dynamically such that it decreases when foreground identification was ambiguous (several objects), or aborted (no objects).

$$t'_{1/2} = \begin{cases} 1.2 \times t_{1/2} & \text{, if unambiguous foreground} \\ 1.2^{-1} \times t_{1/2} & \text{, otherwise.} \end{cases} \tag{6}$$

In addition, the background is selectively updated using the last valid foreground object as a mask [2, 3].

The foreground model is updated with the features from any detected foreground object. Features are stored, in a rolling buffer, up to a maximum of 10^3 observations.

FPS-dependent velocity correction

It was noticed that resulting velocity computation depended on the frame rate of the processed video. It is difficult to predict analytically the relationship between measured velocity and Frame Per Second (FPS) since it depends both on the structure of the background noise and the types of foreground movements. Therefore, we decided to proceed empirically (Fig. 2).

We downsampled the original validation video (144h at 25 FPS, see Fig. 4A of the main article) to eight different new ones with a frame rate between 1 and 5 FPS. Then, we run the tracking

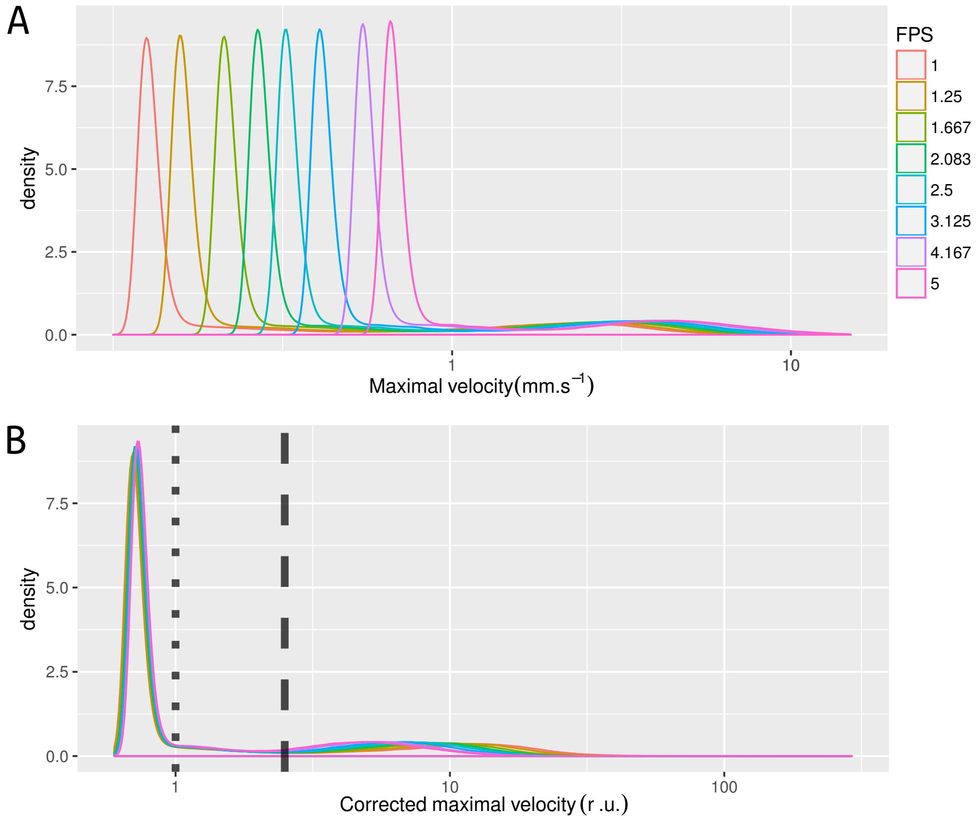

Figure 2: *Distribution of maximal velocity before and after correction* Maximal velocities were computed within each consecutive 10s epoch, from a 144h video with twenty animals, resampled between 1 and 5 FPS. Both figures show the distribution (density) for all frame rates (FPS). **A**, Raw velocities (no correction). Calculation of maximal velocity greatly depends on FPS. Higher FPS artificially results in higher measured velocities. **B**, Corrected velocity. After linear correction, densities of maximal velocity coincide for all FPS. Expressed in relative units. The dotted ($x = 1.0$) and dashed ($x = 2.5$) lines indicate thresholds above which an animal is considered moving and walking, respectively.

algorithm described above on each of them independently and computed all instantaneous velocities (Fig. 2A). The maximal velocity (variable chosen for behavioural scoring, see figure 4B of the main article) in each consecutive 10s epoch was computed in all cases. Ground truth data was used to perform ROC curves and, for each FPS, define the threshold that lead to a False Positive Rate (FPR) of movement equals to 0.5% ($T_{FPS=0.005}$). The relationship between FPS and max velocity was then modeled with linear regression:

$$T_{FPS=0.005} = a \times FPS + b \tag{7}$$

Fitting gave $a = 0.003$ and b was not statistically significantly different from zero, so we used $b = 0$

Therefore, velocity (V_{corr}) calculation were corrected with:

$$V_{corr} = \frac{V}{a \times FPS} \tag{8}$$

This way, $max(V_{corr}) > 1$ indicates the foreground is moving, with with a specificity of 99.5%

To further ensure effectiveness of this method, the density of all instantaneous velocity was re-computed after correction (Fig. 2B). Visual inspection confirmed the performance of our empirical correction.

Software implementation and `R` package

Tracking algorithm was implemented using OpenCV[4] (`python` bindings) in combination with `numpy`[5] and `scipy`[6] libraries.

Acquisition of several days of behavioural data from multiple animals generates a fairly large amount of data (*e.g.*. $25MB \cdot week^{-1} \cdot animal^{-1}$). In order to simplify the behavioural data visualisation and analysis `rethomics`, a novel package for `R` statistical software[7], was developed as part of this project (`https://github.com/gilestrolab/rethomics`).

References

[1] Q. Geissmann, "OpenCFU, a New Free and Open-Source Software to Count Cell Colonies and Other Circular Objects," *PLoS ONE*, vol. 8, p. e54072, Feb. 2013. 00001.

[2] D. Koller, J. Weber, T. Huang, J. Malik, G. Ogasawara, B. Rao, and S. Russell, "Towards robust automatic traffic scene analysis in real-time," in *, Proceedings of the 12th IAPR International Conference on Pattern Recognition, 1994. Vol. 1 - Conference A: Computer Vision amp; Image Processing*, vol. 1, pp. 126–131 vol.1, Oct. 1994. 00518.

[3] M. Piccardi, "Background subtraction techniques: a review," in *2004 IEEE International Conference on Systems, Man and Cybernetics*, vol. 4, pp. 3099–3104 vol.4, Oct. 2004. 01533.

[4] G. Bradski, "The OpenCV Library," *Dr. Dobbs Journal of Software Tools*, 2000. 03691.

[5] S. v. d. Walt, S. C. Colbert, and G. Varoquaux, "The NumPy Array: A Structure for Efficient Numerical Computation," *Computing in Science & Engineering*, vol. 13, pp. 22–30, Mar. 2011. 00066.

[6] E. Jones, T. Oliphant, P. Peterson, and others, *SciPy: Open source scientific tools for Python.* 2001. 00816 [Online; accessed 2015-06-29].

[7] R Core Team, *R: A Language and Environment for Statistical Computing.* Vienna, Austria: R Foundation for Statistical Computing, 2017.
